# A Switch in Iron Delivery Is Critical for Postnatal Kidney Development

**DOI:** 10.34067/KID.0000001064

**Published:** 2026-02-17

**Authors:** Andong Qiu, Melanie Viltard, Rong Deng, Jacob Stauber, Aryan Ghotra, Max Werth, Christian Hinze, Andrew Beenken, Tian H. Shen, Atlas Khan, Katherine Xu, Abraham Levitman, Yue Yu, Neal Paragas, Andrew Yaeh, Beatriz Desanti de Oliveira, Roger W. Boles, Efrat Bruck, Kaitlyn Corbin, Kristen McNierney, Lai Kuan Dionne, Christian Rosenberger, Kai Schmidt-Ott, Thomas Carroll, Moe R. Mahjoub, Rosemary Sampogna, Jonathan Barasch

**Affiliations:** 1 Division of Nephrology, Department of Medicine, Columbia University, New York, New York; 2 School of Life Sciences and Biotechnology, Advanced Institute for Translational Medicine, Tongji University, Shanghai, China; 3 Fondation pour la Recherche en Physiologie, Brussels, Belgium; 4 Albert Einstein College of Medicine and Montefiore Medical Center, Bronx, New York; 5 Klinik für Nieren- und Hochdruckerkrankungen, Zentrum Innere Medizin, Medizinische Hochschule Hannover, Hanover, Germany; 6 Department of Radiology, University of Washington, Seattle, Washington; 7 Division of General Medicine, Department of Medicine, Columbia University, New York, New York; 8 Division of Nephrology, Department of Medicine, Washington University, St Louis, Missouri; 9 KfH Dialysis Center, Erfurt, Germany; 10 Departments of Molecular Biology and Internal Medicine, UT Southwestern Medical Center, Dallas, Texas

**Keywords:** epithelial, kidney development, kidney failure, malnutrition, mineral metabolism, nutrition

## Abstract

**Key Points:**

- Transferrin receptor 1 is critical for perinatal nephron growth and maturation; its deletion results in widespread cystic hypodysplasia.
- Nontransferrin bound iron traffic is a physiologic source of iron in the midgestation embryo and complements transferrin receptor 1.
- Cystic hypodysplasia can be rescued by exogenous iron or by the activation of systemic iron traffic with hypoxia inducible factor activators.

**Background:**

Periconceptual maternal iron deficiency (FeD) is a worldwide cause of premature births and low birth weights. Yet, it is unknown whether FeD affects all developing tissues equally or rather target-*s*pecific lineages. In addition, since FeD restricts both transferrin bound and nontransferrin bound iron species, their unique contributions to organogenesis are indeterminant.

**Methods:**

To address questions of iron traffic and kidney development, we examined the deletion of the singular transferrin receptor (*TfR1^−/−^*), created green flourescent protein-labeled *TfR1^−/−^* embryonic stem cells for inoculation into wild blastocysts, and created *TfR1*-floxed mice to generate cell autonomous deletions of *TfR1* in mesenchymal, ureteric, and stromal derivatives. Finally, we created a model of global FeD with iron poor diets, for comparison with cell autonomous *TfR1* deletions.

**Results:**

Transferrin receptor deletions only modestly suppressed tubulogenesis, had little, if any effect on the growth of the ureteric bud and no gross effects on kidney stroma at mid gestation. By contrast, nutritional FeD nearly abolished kidney development, highlighting the limited phenotypes induced by transferrin receptor deletion. Yet, in the second postnatal week, the critical function of *TfR1* became evident by the growth of residual *TfR1^+^* cells that had escaped Cre-mediated deletion and by tubular segment-specific polycystic transformation. Timed treatment with iron or systemic activators of iron trafficking prevented both cystic dysplasia and the terminal loss of kidney function, reversing extensive malformations of the kidney.

**Conclusions:**

*TfR1* is the critical iron species targeting postnatal tubulogenesis, but in the embryo, *TfR1* must be complemented by alternative iron species called nontransferrin bound iron. Iron-deficient kidney disease is reversible postnatally.

## Visual Abstract

**Figure:**
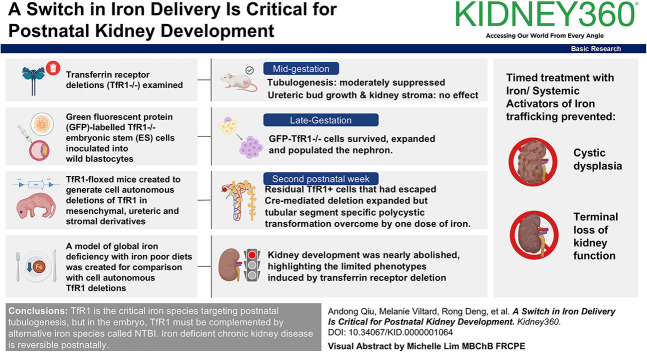

## Introduction

Embryonic growth and development generally depend on water-soluble nutrients such as glucose and amino acids. By contrast, iron (Fe^3+^) is insoluble in water (Ksp <10^−9^ M) and phosphate containing buffers (Ksp <10^−12^ M).^1,2^ In addition, iron is tightly bound to food phytates.^3,4^ The limited bioavailability of iron is the root cause of iron deficiency (FeD), complicating pregnancies with preterm deliveries, low birth weights, and elevated mortality.^5–7^

The delivery of iron to cells is critical for organogenesis. This is because iron serves at the catalytic site in many proteins, including cytochromes,^8^ enzymes which synthesize DNA,^9^ and regulatory transcription factors.^10,11^ Hence, it is not surprising that maternal FeD causes organ hypoplasia with sequelae long after birth, including postnatal cognitive disturbances,^12,13^ cardiovascular defects,^14^ and postnatal hypertension due to kidney hypoplasia.^15^ FeD causes embryonic lethality.^16–18^ In sum, a failure to obtain iron generates quantifiable defects in organogenesis and limits postnatal function.

Specialized carriers are required to deliver Fe^3+^. In adults, iron transport is dominated by transferrin (Tf) and its receptor, *TfR1* (*TfR1* or *TfRC*), but transferrin is synthesized in the embryo only after midgestation,^19–21^ questioning whether *TfR1* serves global role in iron delivery. Extracellular Fe^3+^ is also transported in the blood by nontransferrin proteins (*e.g*., albumin, ferritin, lipocalin2), organic (*e.g*., citrate, ascorbate, heme, catechol) and inorganic molecules (*e.g*., phosphate, soluble Fe^2+^), collectively nontransferrin bound iron (NTBI),^22–24^ found in adult and fetal serum, amniotic, and coelomic fluids,^22,24–27^ but its physiologic role in organogenesis is unknown. Given that iron is closely linked to growth, analysis of iron trafficking pathways may be relevant to the approximately ten-fold variation in nephron number in humans.^28–30^

Here, we examine *TfR1*-mediated iron transport in kidney organogenesis. The developing kidney expresses well-known iron transporters,^31^ and its growth is dependent on the supply of iron.^32,33^ In addition, the sequential stages of kidney development are well characterized,^34,35^ providing metrics to identify the effect of different forms of FeD. We created a series of cell autonomous, lineage-restricted *TfR1* deletions (transferrin receptor deletion [TfD]), and a cell nonautonomous model of periconceptual nutritional iron starvation (FeD). TfD generated modest defects at midgestation compared with FeD. By contrast, after birth, TfD produced polycystic tubules, severe renal failure, and systemic abnormalities of iron transport, each rescued by iron. These data implicate stage and cell-specific roles of transferrin (after midgestation) and NTBI (before midgestation).

## Methods

Supplemental Methods and Supplemental Table 1 contain detailed descriptions.

### Animals

Our study adheres Columbia-Institutional Animal Care and Use Committee and to the National Institutes of Health (NIH) guide for laboratory animals. Mice were maintained in an Association for Assessment and Accreditation of Laboratory Animal Care-accredited facility in 12-hour light-dark cycle.^36^ Mice were fed iron sufficient (220 ppm iron, PicoLab 5053; FeS group) or a synthetic iron-deficient (2–6 ppm iron, TD.80396, Harlan; FeD group) diet for 3 weeks before mating and throughout pregnancy. We chose this schedule because: (*1*) inadequate iron consumption is responsible for the majority of the iron deficient pregnancies^37,38^ and (*2*) iron-deficient diets are effective when initiated 2–3 weeks before conception.^36,39^ Plug date was designated as E0.5. Male offspring and female offspring were used in equal proportion. No mice were excluded from the study.

### Deletion of *TfR1*

The transmembrane domain of *TfR1* (exon 3–4) was floxed using C57BL/6 bacterial artificial chromosome (BAC) DNA clone (RP23159G11; https://bacpacresources.org, Oakland, CA) and standard BAC recombineering techniques. *TfR1*-targeted BAC DNA was electroporated into KV1 embryonic stem (ES) cells (HICCC Transgenic Shared Resource, Columbia) and the integration validated by PCR and Southern Blotting (Supplemental Figure 3). *TfR1*^f/+^ females×EIIa-Cre males (Jackson Laboratory) removed Neo^r^. Mating with *Foxd1-Cre*,^40^ *Six2-CreEGFP,*^41^ and *Hoxb7-Cre*^42^ produced tissue-specific deletions. Cre activity was confirmed by mating with Rosa-Tomato-green flourescent protein mice.^43^

*Six2CreEGFP*; *TfR1*^f/f^ were mated with *HIF*^*f/f*^ mice (www.jax.org/strain/007561). hypoxia inducible factor (*Hif1alpha*) deletion was confirmed by Sanger sequencing. *Rosa26-Gfp*-*TfR1*^*−/−*^ ES were generated, as described.^22^

*Six2CreEGFP*; *TfR1*^f/f^ mice were rescued by iron sucrose (1 mg, Venofer intraperitoneal at P8) or FG-4592 (2 mg/kg; Roxadustat; Cayman Chemical in 1% DMSO; intraperitoneal at P8, P9, P10).

### Quantitative Measurements

#### Hematocrit

I-STAT EC-8+ (Abbott Point-of Care). Nonheme Iron: wet washing procedure^44^ using chromogen bathophenanthroline.

#### Quantitative RT-PCR

Total RNA was reverse-transcribed using the High-Capacity cDNA Reverse Transcription Kit (Applied Biosystems), followed by real-time PCR (Applied Biosystems). Primers: Supplemental Table 1.

#### RNA-Seq

Total RNA was isolated using Ambion TRIzol Reagent (Life Technologies, Carlsbad, CA).

Poly-A pull-down enriched mRNAs (approximately 400 ng per sample, sample RNA integrity number was >6.0) and libraries were prepared using Illumina TruSeq RNA kits and sequenced with Illumina HiSeq2000 (Columbia Genome Center) and data processed with consensus assessment of sequence and variation (v 1.8.2), quality control (https://www.bioinformatics.babraham.ac.uk/projects/fastqc/), trimming and automatic,^45^ TOPHAT v2.0.11,^46^ high-throughput sequencing,^47^ differential expression sequences,^48^ database for integrating human functional interaction networks.^49^ Data were deposited in gene expression omnibus (GEO) https://www.ncbi.nlm.nih.gov/geo/query/acc.cgi?acc=GSE100254.

### Immunofluorescence, Immunoblots, and Three-Dimensional Analysis

Hypoxia inducible factor (HIF) staining by protocol.^50,51^ 5-ethynyl-2′-deoxyuridine (EDU; 50 mg/g) was detected using Click-iT EDU Alexa Fluor (ThermoFischer Scientific). Terminal Deoxynucleotidyl Transferase–mediated deoxyuridine triphosphate nick end labeling (Roche Diagnostics).

Thick cryo-sections (150 *μ*m) or whole permeabilized and cleared kidneys (Focus Clear; Cedarlane Laboratories) were used for three-dimensional analysis. ImageJ Image Layering Toolkit,^52^ Neurolucida^53,54^ quantified number, shape, thickness and volume of Glomeruli, and proximal tubules (PTs) and ureteric bud (UB) branch generation and (*1*) stalk thickness, (*2*) stalk length, (*3*) branch angle, (*4*) branching nodes, (*5*) end points, and (*6*) volume. Cystic index was defined in hematoxylin and eosin sections as the sum of the areas of individual cysts >50 *µ*m in diameter (*i.e*., area approximately 2000 *µ*m^2^) compared with total kidney area.

### Analysis of Cilia

Mahjoub and colleagues^55^ analyzed cilia. Images were collected with Nikon Eclipse Ti-E inverted confocal microscope. Digital optical sections (Z-stacks) were reconstructed as three-dimensional images using Nikon Elements Advanced Research 4.20 and Photoshop software and ciliary length measured using integrated image analysis software. Arl13b fluorescence/unit length cilium was measured with anti-Arl13b and anti–Acetylated-tubulin.

### Statistical Analysis

Figure legends included sample size and *P*, q-values. Two groups were tested by Welch two-tailed test (parametric data) or by Mann–Whitney *U t* test (nonparametric data). Multiple experimental groups with one independent variable were tested using Kruskal–Wallis ANOVA, followed by the Dunn *post hoc* test. Two-way ANOVA was used for comparison of two independent variables on one outcome corrected with either Bonferroni or a two-stage false discovery rate with Benjamini-Krieger-Yukutiel correction for multiple testing. Metrics of tubular growth were normalized to their own control mean values identified in littermates. All measurements were considered continuous variables. Power Analysis: Assuming an independent sample of five knockouts and five controls, we have 80% power to detect at least a two-fold difference in group means at *α*=0.05. This large effect size is expected^56^ due to the crucial role of iron and the success of *TfR1* deletion. Statistics were calculated with Graph Pad 10.0.3.

## Results

### Transferrin Receptor Is Dispensable for Early Metanephric Development

**Figure 1:**
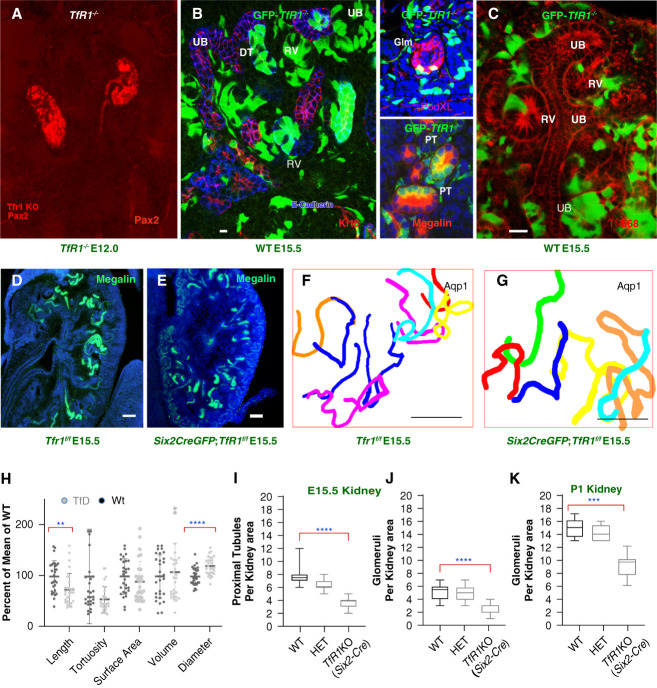
**Initiation of tubulogenesis in *TfR1***^***−/−***^ **mice.** (A) Kidney development in *TfR1*^*−/−*^ embryos (TfD, E12). Pax2^+^ marks mesenchyme and UBs. (B and C) Innoculated *GFP*-*TfR1*^*−/−*^ ES cells populated WT Krt8^+^ E-cadherin^+^ UB, E-cadherin^+^ RVs and DTs, as well as PodXL^+^ glomeruli Glm and megalin^+^ PTs. (C) *GFP*-*TfR1*^*−/−*^ cells cannot capture Holo-Transferrin-Alexa568 (20 *μ*g/ml; 2 hours). (D and E) Megalin^+^ and (F and G) Aqp1^+^ PT immunostaining and 3D tracings (E15). (H) Suppressed linear growth (25.8% shorter; *P*adj = 0.007) in *Six2CreGFP*, *TfR1*^*f/f*^ PT, but volume, surface area, and tortuosity were unchanged. Circumferential growth (increased 20.5%; *P*adj < 0.001). Twenty-nine TfD (seven mice) and 31 WT (eight mice) tubules depicted as % of normalized WT means±SD. Two tailed Mann–Whitney *U* test, Bonferroni correction. (I–K) *Six2Cre*;*TfR1* suppressed the production of nephrons approximately 50%, at E15.5 (megalin^+^ PT and PodXL^+^ glomeruli). However, despite TfD, PodXL^+^ glomeruli (per 20× high power field) increased three-fold between E15.5 and P1. At E15.5 WT PT: 7.71±1.27 versus TfD PT: 3.68±0.72 and E15.5 WT glomeruli 5.32±1.12 versus TfD glomeruli 2.3±0.66; No.=110 sections; eight mice; *P*adj < 0.001. At P1: WT glomeruli: 14.8±1.48 versus TfD glomeruli: 10.6±0.89; No.=29 sections; six mice; *P*adj < 0.001. Kruskal–Wallis; Dunn correction. (B–E) bars=10 *µ*m; (F and G) bars=100 *µ*m; **P < 0.01; ***P < 0.001; ****P < 0.0001. DT, distal tubule; ES, embryonic stem; GFP, green flourescent protein; HET, heterozygote; PT, proximal tubule; RV, renal vesicle; TfD, transferrin receptor deletion; *TfR1*KO, *TfR1* knockout; UB, ureteric bud; WT, wild type.

To evaluate the contribution of transferrin to the embryonic kidney, we first documented the expression of its receptor, *TfR1*. *TfR1* was expressed throughout the metanephric mesenchyme and UB, particularly in the cortex (embryonic day, E12.5–15.5; Supplemental Figures 1 and 2). Yet, metanephric and ureteric development persisted in *TfR1*^*−/−*^ knockouts at E11-E12, depicted by Pax2 staining^6,22^ (Figure 1A). In fact, green flourescent protein-*TfR1*^*−/−*^ ES cells, introduced into wild-type blastocysts, differentiated into embryonic renal vesicles and glomerular and proximal tubular epithelia^22^ (E15.5–E16; Figure 1B) in register with surrounding *TfR1*^*+*^cells (Figure 1C).

The survival of *TfR1*^*−/−*^ cells and kidneys indicated that *TfR1* was not required in E11 kidney. To resolve whether transferrin was required in a stage-specific or tissue-specific manner, we created *TfR1*^*f/f*^ mice by floxing exons 3–4 (Supplemental Figure 3) and deleted *TfR1* in different kidney lineages. The conditional approach (TfD) circumvented both the lethality of *TfR1*-null mice after E12^6^ and the contributions of *TfR1* to placental physiology.^27^ *TfR1* deletion in nephron progenitors by *Six2CreEGFP*^41^ did not grossly affect their cortical positioning adjacent to the UB nor did TfD abolish conversion of nephron progenitors into renal vesicles, glomeruli, or PTs (Figure 1, D and E and Supplemental Figure 1). Three-dimensional reconstructions of the PT (Figure 1, F and G) demonstrated limited decrease in length (−25.8%; *P*adj = 0.007), normal volume (*P*adj = 4.26), surface area (*P*adj = 0.68), tortuosity (*P*adj = 0.086), and increased diameter (+20.5%; *P*adj < 0.001) of tubules (Figure 1H). In fact, despite reduction of glomeruli (PodXL^+^) and PTs (megalin^+^) by 50% (E15.5; No.=8 mice; *P*adj < 0.001), TfD nephrons were generated throughout gestation (P1; No.=6 mice; *P*adj < 0.001; Figure 1, I–K).

*TfR1* was also highly expressed UB cells, but deletion of *TfR1* by *Hoxb7Cre* did not abolish ureteric development (Supplemental Figure 2) nor grossly distort kidney structure. At P8, 18/20 kidneys were grossly normal, 1/20 kidneys demonstrated hypoplasia, and 1/20 demonstrated hydronephrosis (No.=10 litters; Supplemental Figure 4A).

*TfR1* was not expressed in stroma^22^ nor did we detect a phenotype upon deletion of *TfR1* with stromal *FoxD1Cre* (Supplemental Figure 4B).

In summary, TfD reduced nephron number and produced subtle tubular hypoplasia. Yet, PTs developed in *Six2CreEGFP*; *Tfr1*^*f/f*^ kidneys and gross defects in mesenchymal conversion and tubular morphogenesis were less severe than predicted by the ubiquity of *TfR1* and its dominant role in the adult.

### Transferrin Receptor Is Required for the Growth of Nephron Segments (P8–P13)

**Figure 2:**
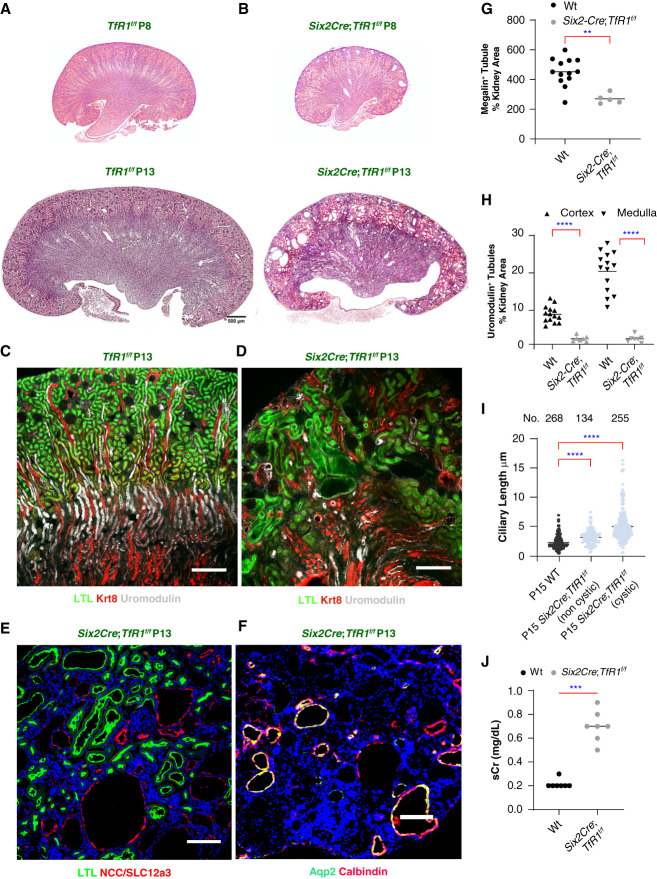
**Deletion of *TfR1* in the nephron resulted in cystic hypodysplasia after birth**. (A and B) *Six2CreEGFP*; *TfR1*^f/f^ kidneys demonstrated cortical cystic transformation (P8–P13). (C and D) There was a 40%±7% decrease in megalin^+^ PT/kidney area (*P* = 0.003; WT No.=13, TfD No.=5) and 81%±9% decrease in cortical and 92%±5% decrease in medullary uromodulin^+^ TALH/kidney area (*P* < 0.001; WT No.=14; KO No.=7 mice). (E–H) *Six2Cre*EGFP; *TfR1*^f/f^ produced fewer, shorter, dilated LTL^+^ PT and almost no uromodulin^+^ TALH. Tubule diameter increased 68%±94% (*P* = 0.021). Cysts expressed markers of PT: megalin^+^ or LTL^+^; TALH: uromodulin^+^; DCT: NCC/Slc12a3^+^; and connecting segments: note overlap of Calbindin^+^; Aqp2^+^ (producing yellow color). (I) Cilia were nearly twice as long P15 TfD cystic tubules (*P* < 0.001), but even noncystic tubules demonstrated longer cilia (*P* < 0.001). (J) sCr 0.7±0.13 *Six2Cre*; *TfR1^f/f^* No.=7 mice versus sCr 0.21±0.035 *TfR1^f/f^* No.=9 mice; *P* < 0.001. (G and H) Mann–Whitney *U* test with H Bonferroni correction. Mean±SD. **P < 0.01; ***P < 0.001; ****P < 0.0001. (A and B) Bars=500 *μ*m; (C–F) bars=200 *μ*m. DCT, distal convoluted tubule; KO, knockout; LTL, *Lotus tetragonolobus* lectin; NCC, sodium-chloride cotransporter; sCr, serum creatinine; TALH, thick ascending limbs of Henle.

**Figure 3:**
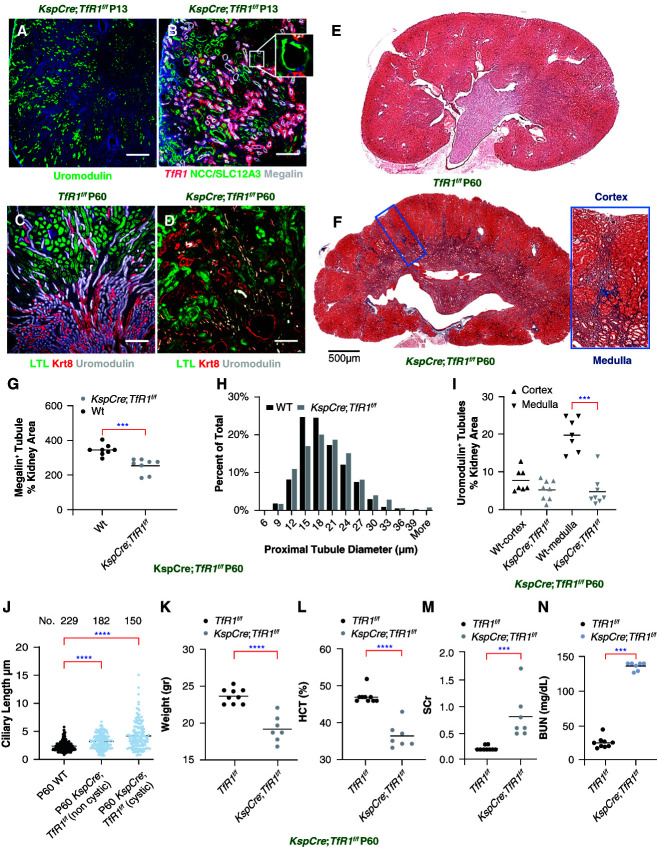
**Deletion of *TfR1* in the distal nephron by *KspCre*; *TfR1***^**f/f**^. (A and B) P13: disorganized, fragmented uromodulin^+^ TALH (compare Figure 2C) and cystic NCC/SLC12A3^+^ DCT, yet spared *TfR1^+^* megalin^+^ cortical PT. (C and D) P60: further loss of uromodulin^+^ TALH and cystic Krt8^+^ UB, yet spared PT (LTL^+^). (E and F) Masson-Trichrome^+^ bands extended from cortico-medullary junction to dimple the cortex. Inset (outlined). (G) P60: limited PT hypoplasia (26%±12% decrease in megalin^+^ tubule counts; *P*adj < 0.001; No. =8) and (H) similar PT tubular diameter (*n*=8). (I) Limited cortical hypoplasia, yet medullary TALH tubules demonstrated 76%±21% loss (*P*adj = 0.001; No. =7–8 each). (J) Ciliary length was prominently longer in P60 TfD: cystic *P*adj < 0.001, noncystic tubules *P*adj < 0.001. (K–N) Mice suffered weight loss (22.6%; No. =7–9), anemia (23.5%; *n*=7–9) *P* < 0.001, and kidney failure: four-fold elevated sCr (No.=7), 4.9-fold elevated BUN (No.=7); *P* < 0.001. (G, I, and K–N) Mann–Whitney two-tailed test, (I and K–N) Bonferroni correction for multiple testing; Mean±SD. (J) Kruskal–Wallis corrected by Dunn test. No.= 6–10 mice were used for microscopic analysis. Mean±SD. ***P < 0.001; ****P < 0.0001. (A–D) Bars=200 *µ*m; (E and F) bars=500 *µ*m.

We studied *TfR1* in later stages of kidney development. *Six2CreEGFP*; *TfR1*^f/f^ kidneys were morphologically normal at birth, but by the end of the second postnatal week, proliferative failure was apparent in both cortical megalin^+^ PTs (64% reduction: 53.2±7.56 versus 19.2±2.58 EDU^+^ cells/area 630×630 *μ*m^2^ *P*adj < 0.001; No.=5 each) and medullary uromodulin^+^ Loops of Henle (71% reduction: 92±27 versus 27.4±27 EDU^+^ cells/area 630×630 *μ*m^2^ *P*adj < 0.001; No. =5 each). In addition, extensive apoptosis at the corticomedullary junction was found at P13–15 (Supplemental Figure 5). RNA-seq analysis of *Six2CreEGFP*; *TfR1*^f/f^ kidneys reflected the proliferative failure, identifying 441 downregulated genes (≥2.0-fold; *P*adj < 0.05) that were enriched in cell cycle pathways and membrane transport pathways, which probably reflected tubular hypoplasia (No. =3; *P*adj < 0.001; Figures 2 and 3 and Supplemental Figures 6 and 8).

Although organized kidney architecture was evident before birth (Figure 1), TfD-induced widespread morphogenic disruption during the second postnatal week. A few scattered tubular cysts were present at P8–P9, but by P13–P15 numerous large cortical and cortico-medullary cysts disorganized the architecture of the cortex and cortico-medullary junction (Figure 2, A and B). Segment-specific markers depicted cortical PT dilation and cysts (*Lotus tetragonolobus* lectin^+^ and megalin^+^; Figure 2, C and D and Supplement Figure 7A), thick ascending limbs of Henle (TALH; uromodulin^+^) and cortical distal convoluted tubule dilation, and cysts (sodium-chloride cotransporter/Slc12A3^+^; Figure 2, C–E and Supplemental Figures 7, B and C, and 8) and junctional cysts (AQP^+^, Calbindin^+^) apparently where nephron and cortical collecting ducts fused (Figure 2F and Supplemental Figures 7D and 8). The largest cysts did not express segment-specific markers. Quantitative measurements demonstrated stunted PT and even the near absence of TALH tubules (Figure 2, G and H). Because disruption of ciliary function results in cystic-dysplastic kidney diseases,^57,58^ we measured a number of attributes of ciliary structure (see Supplemental Figure 13) and found ciliary elongation in both cystic tubules and noncystic neighbors, suggesting that ciliary elongation preceded cystogenesis (Figure 2I). Eventually, TfD caused lethal kidney failure (Figure 2J; serum creatinine rose from 0.21±0.035 to 0.7±0.13, No. =7, 9; *P* < 0.001); mortality neared 100% by P15.

To test whether the proliferative and morphogenic failure was directly attributable to the knockout of *TfR1*, we used a second driver, *Ksp1.3Cre,* to delete *TfR1* in a different segment.^59^ Consistent with the distribution of this driver, the knockout nearly abolished cortical and medullary TALH uromodulin^+^ tubules at P13 (Figure 3, A and B), reducing EDU^+^ labeling by 84% (80±16.7 versus 13.25±4.06 EDU^+^ cells/area 630× 630 *μ*m^2^; *P*adj < 0.001; *n*=8 each) in remnants of the loop. By contrast, the PT was little affected by *KspCre* mediated *TfR1* deletion (54±5.97 versus 50.6±4.39 EDU^+^ cells/area 630×630 *μ*m^2^; *P*adj = NS; No. =5 each). Small cysts and dilated tubules were found in the distal convoluted tubule (sodium-chloride cotransporter/Slc12A3^+^) and collecting ducts (Kertain8^+^; Krt8^+^) in 44% of kidneys by P13, but most kidneys were affected by P60 (No. =5 litters, Figure 3, B–D). Ultimately, bands of Masson-Trichrome^+^ fibrosis surrounded the cysts and dimpled the surface where fibrotic bands reached from the cortico-medullary junction to the kidney capsule (Figure 3, E and F). These data contrast with cortical PT at P60: in the cortex, megalin expression, tubule morphology, and *Lotus tetragonolobus* lectin^+^ tubules were little affected at P60 (26%±12%; *P* < 0.001; Figure 3D and G and H), whereas cortico-medullary TALH were depleted (76%±21%; *P*adj < 0.001; Figure 3I); ciliary elongation in cystic tubules and noncystic neighbors was found in remnant tubules (Figure 3J). Weight loss (23.2±1.1 to 18.5±1.8 g; No. =9, 7; *P* < 0.001), anemia (46.1±1.9 to 36.3±3.5 %Hct, No. =9, 7; *P* < 0.001), and kidney failure (sCr=0.2±0.04 to 0.814±0.43 mg/dl; BUN 27.1±7.1 to 136.14±5.7 mg/dl; *P*adj < 0.001; No. =7–9) supervened due to medullary defects (Figure 3, K–N). In summary, *Tfr1* is indispensable for postnatal kidney development both in cortical and in cortico-medullary tubules.

### Postnatal Nephron Growth Is Linked to *TfR1,* But Midgestation Growth Is a Composite of Tf and NTBI

**Figure 4:**
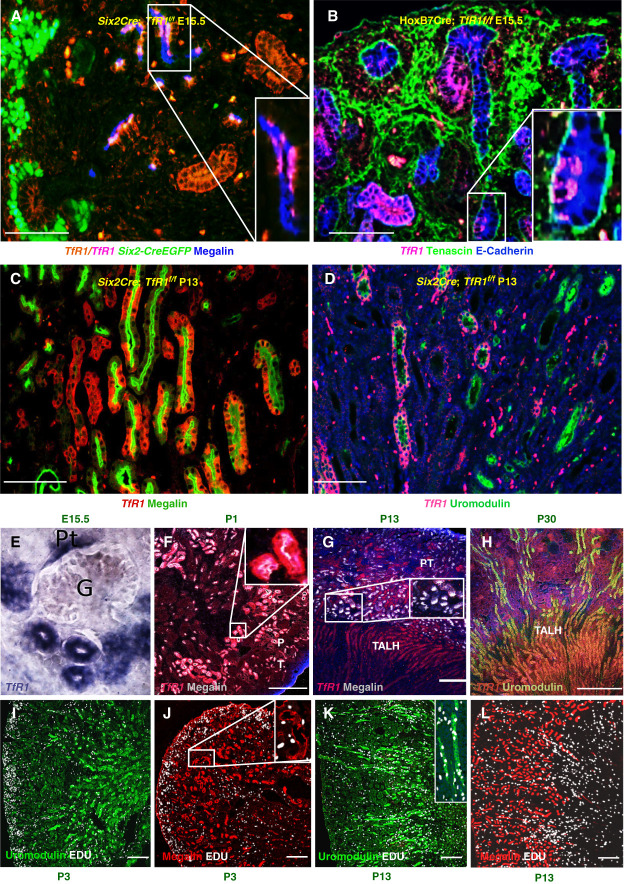
**The deletion of *TfR1* does not limit tubulogenesis, but *TfR1***^***+***^ **cells have a growth advantage.** (A) Residual *TfR1*^+^ cells populate *Six2Cre*; *TfR1*^*f/f*^ megalin^+^ tubules by E15.5. (B) Residual *TfR1*^+^ cells populate *Hoxb7Cre*; *TfR1*^*f/f*^ E-cadherin^+^ UBs by E15.5. (C and D) Residual *TfR1*^+^ cells occupy the majority of *TfR1*^−^ megalin^+^ PT and surviving uromodulin^+^ TALH by P13. Bars=100 *µ*m. (E–L) *TfR1* expression correlated with proliferative zones. (E–H) *TfR1* message was expressed by megalin^+^ PT at E15.5 and P1, but its expression faded by P13. In contrast, *TfR1* was expressed by uromodulin^+^ megalin^+^ TALH tubules at P13 and P30. (I–L) Cell proliferation followed a similar pattern. Cortical mesenchyme and megalin^+^ uromodulin^−^ PT captured EDU at P3, but subsequently, megalin^−^ uromodulin^+^ TALH captured EDU. (F–L) Bars=200 *µ*m. EDU, 5-ethynyl-2′-deoxyuridine.

An unexpected clue to *TfR1* function resulted from its incomplete deletion by *Six2Cre*EGFP.^41,60^ *Six2Cre*EGFP is active in all tubule progenitors, but a few scattered *TfR1*^+^ cells that apparently had escaped Cre-mediated deletion were visible days later in developing PTs at E15.5. Similarly, *TfR1*^+^ cells that had escaped *Hoxb7Cre*-mediated deletion were visible in collecting ducts at midgestation^61^ (Figure 4, A and B). Three weeks later, by P13, *TfR1*^+^ cells populated the nephron: 58%±8% of megalin^+^ PTs contained *TfR1*^+^ rogue cells, side by side with *TfR1*^−^ cells (Figure 4C) and approximately 90% of surviving uromodulin^+^ TALH tubules were composed entirely of *TfR1*^+^ cells (Figure 4D). The emergence of *TfR1*^+^ cells toward birth and their colonization of the nephron implied a growth advantage conferred by *Tfr1* expression compared with neighboring *TfR1*-null cells.

The presumptive growth advantage of *TfR1^+^* cell led us to consider the timing of both *TfR1* expression and proliferation in postnatal kidneys. A shifting pattern in *TfR1* expression emerged from the proximal to distal nephron which paralleled the spatial distribution of proliferation (Figure 4, E–L). For example, *TfR1* was expressed in cortical PTs at P1, but expression faded in the second postnatal week (Figure 4, E–H). Similarly, EDU labeling was observed in cortical PTs at P3 but was subsequently restricted (Figure 4, J and L). In the distal nephron, *TfR1* expression and EDU labeling were also observed in parallel, but after the second postnatal week (P13–P30; Figure 4, G, H, K, and L). In summary, the growth advantage of *TfR1^+^* cells, the redistribution of *TfR1* expression with cellular proliferation, and the pattern of growth failure in *TfR1* knockouts all demonstrate that *TfR1* is responsible for postnatal kidney proliferation and tubulogenesis.

**Figure 5:**
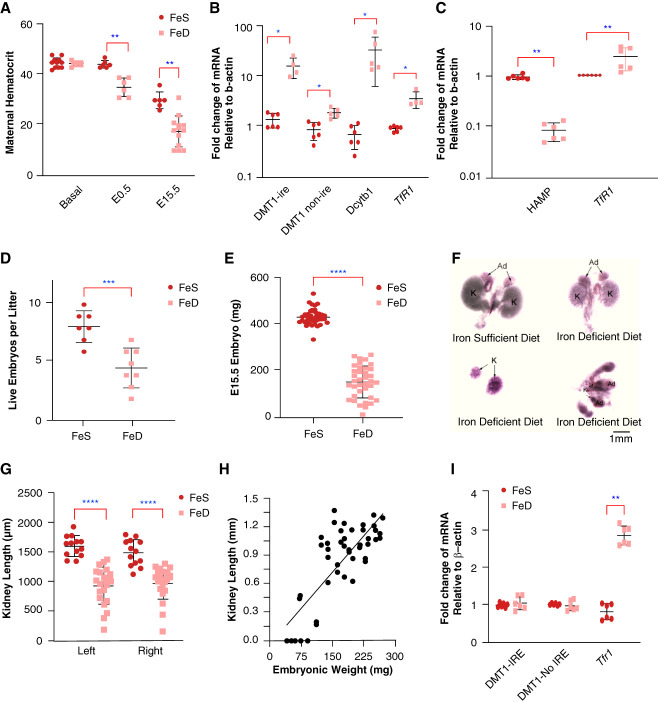
**Maternal and embryonic response to FeD.** (A) Independent cohorts of adult female mice fed low iron diets for 3 weeks and then mated (8 weeks old, FeS No.=11, FeD No.=9; *P* = 0.83). Maternal hematocrit by E0.5 days (FeS and FeD No.=6; *P* = 0.00216) and by E15.5 days of gestation (FeS No.=6 versus FeD No.=13; *P* = 0.0014). (B) By E15.5, FeD upregulated maternal duodenal *DMT1-ire* 12-fold (*P*adj = 0.038), *DMT1-non-ire* two-fold (*P*adj = 0.018), *Dcytb1* 50-fold (*P*adj = 0.017) and *TfR1* four-fold (*P*adj = 0.04; No.=4–6 mice each). (C) By E15.5, FeD upregulated maternal hepatic *TfR1* (2.4-fold; *P*adj = 0.004) and downregulated *Hamp* (11.6-fold; *P*adj = 0.005) No.=6 each; *P < 0.05; **P < 0.01; ***P < 0.001; ****P < 0.0001. (A) Two tailed Mann–Whitney *U* test (B and C) with Bonferroni correction for multiple comparisons. (B) Log_10_ scale for visualization. Data represent mean± SD. (D) FeD reduced litter size (43% decrease, *P* < 0.001, No.=7–8 litters at mid gestation). (E) FeD produced variable degrees of embryonic hypoplasia (65% smaller, *P* < 0.001; FeS No.=41; FeD No.=37), that correlated with (F) variable kidney hypoplasia, (G) kidney length was reduced by 42% left and 34% right by FeD (FeD No.=22 and FeS No.=13 kidneys; left: *P*adj < 0.001; right: *P*adj < 0.001). (H) Embryonic weight correlated with kidney size (R^2^=0.71). Kidneys could not be identified in embryos <75 mg. (I) FeD regulated *TfR1* but not *DMT1-ire* or *DMT-non-ire* in mid gestation kidneys (*P*adj = 0.006; No.=6 each). (D and E) Welch two tailed *t* test, normality testing. (G and I) Two tailed Mann–Whitney *U* test, Bonferroni correction. (H) Least squares regression. Mean± SD. **P < 0.01; ****P < 0.001; ****P < 0.0001. Ad, adrenal; FeD, iron deficiency; FeS, iron sufficient; HAMP, hepcidin antimicrobial peptide.

**Figure 6:**
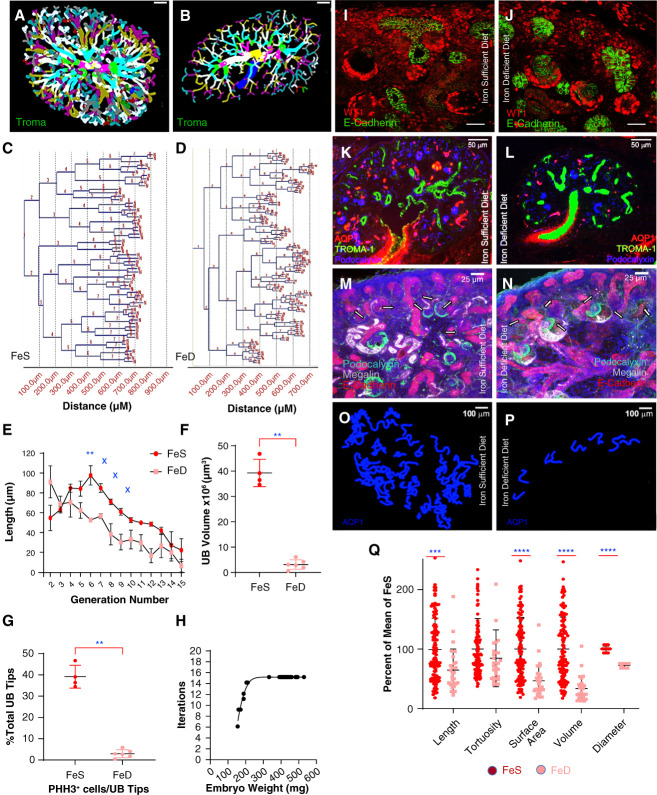
**Maternal FeD inhibited linear growth.** (A–D) Irregular branch points in FeD UB. Troma-1=KRT8. (C–E) Generations six, seven, eight, nine, were shortened particularly six (individual six: *P* = 0.002, q=0.02; seven: *P* = 0.04, q=0.09; eight: *P* = 0.02, q=0.07; nine: *P* = 0.03, q=0.07; No.=5 kidneys). *P < 0.05, q<0.05; x*P* < 0.05, q>0.05; (F) reducing tree volume by 92% (FeS: *P* = 0.01; No.=4 FeS and No.=6 FeD kidneys) and (G) phosphohistone3^+^ labeling per UB tip 40% (No.=239 FeS and No.=135 FeD tips in No.=4 mice each). (H) Nonetheless, total number of branch iterations were similar in FeD and FeS embryos >190 mg (No.=5 mice each; *P* = 0.15). (I and J) Equivalent induction of WT1^+^ cap mesenchyme in both FeS and FeD (E15.5). (K and L) Yet 65% fewer podocalyxin^+^ glomeruli (No.=5 mice, *P* = 0.028) and 92% fewer AQP1^+^ PT (No.=6,4 mice, *P* = 0.009) in FeD than in FeS kidneys. (M–P) Few, short megalin^+^ Aqp1^+^ FeD PT. (Q) Summary= % decrease: linear growth (36%; *P* < 0.001), circumferential growth (28%; *P* < 0.001), volume (66%; *P* < 0.001), and surface area (53%; *P* < 0.001) of Aqp1^+^ PT. No.=118 WT, 29 TfD PT, in five mice each. Data=% control. Means±SD. (E) Two-way ANOVA (Benjamini-Krieger-Yekutieli). (F and Q) Two-tailed Mann–Whitney *U* test; (Q) corrected with Bonferroni. **P < 0.01; ***P <0.001; ****P < 0.0001. Bars (A, B, O, and P) 100 *µ*m; (K and L) 50 *µ*m; (I, J, M, and N) 25 *µ*m.

While *TfR1*, tubular growth, and morphogenesis were linked in postnatal kidneys, the more limited phenotypes in embryonic knockouts implied compensation by NTBI. If both Tf and NTBI contributed to growth in the embryonic kidney, we reasoned that global FeD should produce more severe growth failure than TfD alone. We fed 2–6 ppm iron diet (FeD) to mice for 3 weeks before mating and continued the diet until midgestation. We confirmed that the FeD diet produced classical FeD metrics in the mother^16,27^; reducing hematocrit, upregulating duodenal iron transporters *Dmt1-IRE*, *Dcytb1,* and *TfR1*; and decreasing hepatic hepcidin/*Hamp* (Figure 5, A–C) in comparison with iron sufficient diets (approximately 220 ppm iron; FeS). The FeD model produced severe embryonic hypoplasia (Figure 5, D and E) correlating with defects in kidney growth^37,62,63^ (R^2^=0.62; Supplemental Figure 5, F–H) as well as 44% fetal loss at midgestation (*e.g*., FeD: 4±1.4 versus FeS: 0±0 aborted pups/litter; *P* < 0.001) and 75% loss of the litter by birth. Immunolabeling^64^ and three-dimensional reconstructions at midgestation demonstrated that unlike TfD, FeD severely suppressed the linear growth of both ureteric and mesenchymal tubules (Figure 6). Collecting duct hypoplasia (Figure 6, A–D) was particularly evident in the sixth to eighth branch generation (Figure 6E), producing severely suppressed duct volumes (92%; Figure 6F) coupled with reduced labeling by proliferative marker phosphohistone3^+^ (Figure 6G) despite preserved branch iterations. Variable disruption of 2n bifurcating events^34,53^ and failed iterative branching (<190 mg embryos: FeD=8±1.7 iterations versus >190 mg: FeD=12.8±1.5 iterations, *P* = 0.019, No.=7; FeS=15±1 iterations, *n*=5; Figure 6H) occurred only in hypoplastic embryos <190 mg, not in preserved embryos.

Nephron tubules were also severely hypoplastic in the FeD model (Figure 6, I–N).^34,65–68^ PT numbers were reduced 92%, shortened 36% (*P* < 0.001), and limited in diameter (28%; *P* < 0.001; Supplemental Figure 6, O and P). Phosphohistone3^+^ labeling of nuclei was reduced 36% (FeD No.=24, FeS No.=28; *P* < 0.001) compared with FeS kidneys. In sum, the severe effects of FeD (Supplemental Figure 6Q) contrast with the relative preservation of kidney structures in the TfD model (Figure 1H, comparison: Supplemental Figure 9) consistent with the notion that prenatal development is compensated by NTBI mediated iron transport. NTBI transporters (ZIP8 and ZIP14)^69^ are candidates for compensation since they were upregulated in response to TfD (Supplemental Figure 10).

### Treatment with Iron Rescues Cystic *Six2Cre*EGFP; *TfR1*^f/f^ Mice

**Figure 7:**
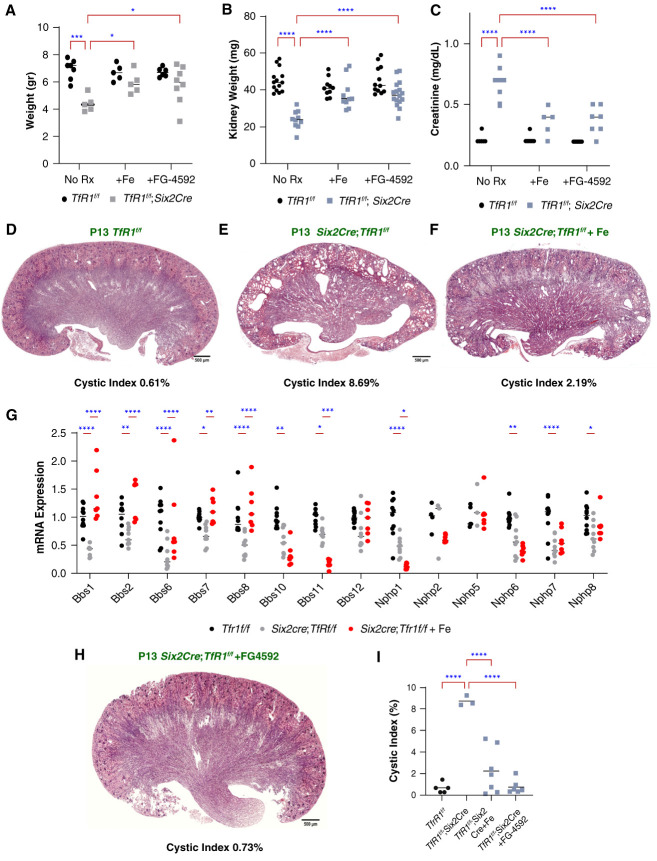
**Correction of cystic hypodysplasia by iron and activation of HIF.** (A) *Six2Cre*EGFP; *TfR1*^f/f^ reduced body weight (*P*indv < 0.001; q<0.001), (B) kidney weight (*P*indv < 0.001; q<0.001), and (C) raised serum creatinine (*P*indv < 0.001; q<0.001). However, a single dose of iron-sucrose (1 mg) at P8 or PHD inhibitor (FG-4592 0.4 mg/kg) at P8, P9, and P10 rescued TfD phenotypes. (A) Treatments normalized TfD body weight from 4.41±0.61 to 5.93±0.81 g with iron (*P*indv < 0.001; q=0.018) or to 5.78±1.37 gr with FG-4592 (*P*indv < 0.001; q=0.018), similar to *TfR1*^f/f^ controls (6.92±0.738 g); No.=5–8 per group. (B) Treatments normalized TfD kidney weight from 23.94±4.94 to 37.72±8.18 mg with iron (*P*indv < 0.001, q<0.001) or to 37.68±6.72 mg with FG-4592 (*P*indv < 0.001, q<0.001), similar to control *TfR1*^f/f^ (45.15±6.25 mg); No.=14, 10, 10, 16 kidneys. (C) Treatments normalized TfD sCr from 0.70±0.12 to 0.36±0.11 mg/dl with iron (*P*indv < 0.001, q<0.001) or 0.37±0.11 mg/dl with FG-4592 (*P*indv < 0.001, q<0.001) similar to controls *TfR1*^f/f^ (0.21±0.035 mg/dl); No.=8, 5, 7 pairs. (A-C) Two-way ANOVA corrected by FDR two stage Benjamini-Krieger-Yukutieli. Mean± SD. *q<0.05; ***q<0.001; ****q<0.0001. (D–I) *Six2Cre*EGFP; *TfR1*^f/f^ induced cysts, but a single dose of iron-sucrose (1 mg) at P8 or PHD inhibitor (FG-4592 0.4 mg/kg) at P8, P9, P10 suppressed cystic growth. (F) Treatment with iron normalized TfD cystic index from 8.69%±0.5% to 2.19%±2.1% (*P* < 0.001). (G) Treatment with iron at P8 (red) normalized BB1, two, six, seven, eight expression (*e.g*., Six2Cre; TfR^f/f^ suppressed BB1 approximately 60%, but the addition of iron normalized expression; *P ≤* 0.001; qRT-PCR), but iron was ineffective for other BBSome and Nphp genes. Two-way ANOVA, corrected with Bonferroni No.= 6–10 mice. *q<0.05; **q<0.001; ***q<0.001; ****q<0.0001. (H). Treatment with FG-4592 normalized TfD cystic index to 0.733%±0.66% (*P* < 0.001) similar to control (0.61±0.48). No.=5, 3, 7, 6 mice. (I) Summary: cystic index=cystic area (*µ*m^2^)/kidney area (*µ*m^2^). Two-way ANOVA with Bonferroni correction for multiple testing. Mean±SD. ****P < 0.0001. FDR, false discovery rate; Nphp, nephronophthisis; PHD, prolyl hydroxylase domain; qRT-PCR, quantitative reverse transcription PCR.

Deletion of *TfR1* implicated FeD as the key mechanism of proliferative and morphogenic failure of the postnatal kidney. To test this hypothesis, we attempted to rescue defects found in *TfR1* deleted kidneys with iron. Administering ferric sucrose (1 mg) at P8 restored body weight, kidney weight, serum Cr (Figure 7, A–C), and even blocked cystic transformation. The cystic index (cystic area *µ*m^2^/total kidney area *µ*m^2^) fell from 8.69%±0.5% to 2.2%±2.1% (*P*indv < 0.001; q<0.001; No.=3 no treatment, No.=7 treatment; Figure 7, D–F), similar to control cyst burden (0.61±0.48). Iron may directly affect cystic epithelia since ciliary cargo adaptors, the BBSomes were suppressed in Six2Cre; *Six2Cre; *TfR1*^f/f^* kidneys but restored with iron (BB1, 2, 6, 7, 8 [Figure 7G]). Hence, hypoplasia and cystic dysplasia could be suppressed by pharmacologic doses of iron, as late as 1 week after birth.

Past studies have linked cell autonomous *HIF1alpha* activation in kidney cells with cystic growth.^70–72^ Given that FeD prevents the degradation of HIF proteins,^50,73^ TfD might lead to HIF activation and subsequent cystogenesis. In fact, postnatal P13–15 RNA-seq identified 353 (in *Six2CreEGFP*; *TfR1*^f/f^) and 693 (in *KspCre*; *TfR1*^f/f^) upregulated genes (≥2.0-fold; *P*adj < 0.05, No.=3 for each model) enriched in the *HIF1alpha* pathway (*P*adj < 0.001; Supplemental Figure 6), confirming a direct linkage between HIF pathways and TfD. However, cysts were still present when both *TfR1* and *Hif1α* were simultaneously deleted with *Six2Cre*EGFP (*Six2Cre*EGFP; *TfR1*^f/f^; *Hif1α*^f/f^, genotype confirmed by PCR and sequencing; Supplemental Figure 11).

To further examine the linkage between FeD, HIF activation, and cystic hypodysplasia, we treated TfD mice, just before widespread cystogenesis at P8, with FG-4592 (Roxadustat), an US Food and Drug Administration-approved HIF prolyl-hydroxylase inhibitor that stabilize HIF protein.^74^ Roxadustat rescued the *Six2CreEGFP*; *TfR1*^f/f^ kidney phenotype (No.=7) and normalized postnatal growth. Roxadustat restored body and kidney weight, reduced serum creatinine (Figure 7, A–C), and reversed the cystic index, which fell to 0.73%±0.66% (*P* < 0.001, q<0.001) similar to *TfR1*^f/f^ controls (0.61±0.48; Figure 7, A–C, H, and I).

**Figure 8:**
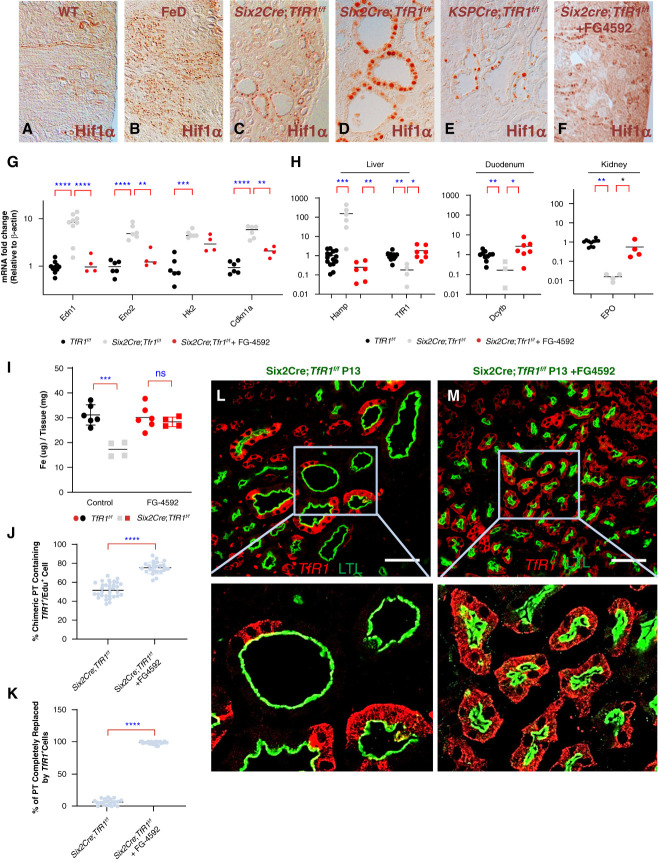
**HIF activation rescues kidney dysgenesis.** (A–E) Upregulated HIF1alpha protein in FeD, *Six2CreEGFP*; *TfR1*^f/f^ and *KspCre*; *TfR1*^f/f^ models. Note the nuclear localization of HIF1*α* in cystic epithelia. (F) HIF1*α* protein expression was upregulated 1 day after treatment with FG-4592. (G) HIF1*α* targeted genes were enriched in *Six2Cre*; *Tfr1*^*f/f*^ mice (*Edn1*, *P*adj < 0.001; *Eno2*, *P*adj < 0.001; *HK2*, *P*adj < 0.001; *Cdkn1a*, *P*adj < 0.001); No.=6–9 versus No.=10–6 WT mice. Treatment with FG-4592 suppressed some of the genes (*e.g*., *Edn1*, *P*adj < 0.001; *Eno2*, *P*adj = 0.0016; *HK2*, *P*adj = 0.3; *Cdkn1a*, *P*adj = 0.008); No.=7. Untreated, age matched *TfR1*^*f/f*^ littermates (WT) served as controls, normalized to one. Kidneys were harvested at P13. *P < 0.05; **P < 0.01; ***P < 0.001. Mean± SD. Two-way ANOVA with Bonferroni correction for multiple testing. (H) *Six2Cre*; *Tfr1*^*f/f*^ reduced expression of Liver *TfR1* (*P*adj = 0.0088), duodenal *Dcytb* (*P*adj = 0.008), kidney *Epo* (*P*adj = 0.003) compared to WT littermates; No.=4 *Six2Cre*; *Tfr1*^*f/f*^, No.=10–12 WT. Treatment with FG-4592 increased *Tfr1* (*P*adj = 0.024), *Dcytb* (*P* = 0.024) and *Epo* (*P* = 0.057); No.= 4–7. Conversely, elevated *Hamp* (*P*adj < 0.001; No.=6, 15) returned to control values (*P*adj = 0.009); No.=6. Mann–Whitney *U* test with Bonferroni corrections. (I). Deletion of *TfR1* reduced nonheme iron (from 31.15±4.09 to 17.25±3.01 mg Fe/mg tissue; No.=6, four mice, respectively; *P*adj < 0.001). FG-4592 normalized iron level (+FG-4592: 28.4±1.9 mg Fe/mg tissue; No.=6, four mice). Two-way ANOVA with Bonferroni correction for multiple testing. (J–M) FG-4592 expanded the population of *TfR1^+^* cells in TfD PT (LTL^+^). (J) FG-4592 increased *TfR1^+^* EDU^+^ cells in chimeric TfD tubules (from 52.0%±7.6% to 75.3%±6.4%; No.=525 and 510 *Tfr1*^+^ cells in 55 sections, five, four mice, respectively; *P* < 0.001). (K) Complete replacement of *TfR1*^−^ by *TfR1^+^* cells in 77.2%±17.7% of PT after treatment with FG-4592 compared to 7.46%±4.61% tubules in untreated mice (No.=1850, No.=684 tubules in 56 sections, four, five mice respectively; *P* < 0.001). *TfR1^+^* was detected in 98.09%±1.88% wild type *TfR1^f/f^* tubules (No.=1099 tubules, 20 sections, five mice). (J and K) Mann–Whitney two-tailed test. ****P < 0.0001. (L and M) Bars=100 *µ*m. HIF, hypoxia inducible factor.

To determine the locus of Roxadustat activity, we first studied HIF expression in the kidney and identified nuclear located HIF in cysts in TfD (consistent with RNAseq, Supplemental Figure 6) and in kidney tubules of Roxadustat treated mice (Figure 8, A–F). However, while TfD elevated downstream HIF target genes in the kidney, Roxadustat suppressed HIF target genes (Figure 8G). While initially counterintuitive given that Roxadustat enhances HIF activation, we considered that the effect of treatment may be due to upregulation of systemic iron transport. Indeed, Roxadustat normalized major iron regulatory genes including liver *TfR1*^75^*,* duodenal *Dcytb*,^76^ kidney erythropoietin (EPO)^77^ and liver hepcidin *Hamp*^78^ (Figure 8H). Perhaps these mechanisms converge to effectively increase kidney iron (*P* < 0.001, Figure 8I) and reduce kidney HIF activation. Hence, it is plausible that Roxadustat stimulated systemic HIF activation which increased iron capture. Consistent with this idea, supplementation with exogenous iron suppressed HIF stimulated gene expression (Supplemental Figure 12).

To further investigate how Roxadustat rescued kidney morphology, we examined cellular proliferation using EDU labeling. We found that Roxadustat stimulated EDU^+^ proliferation of *TfR1*^+^ rogue cells in *Six2CreEGFP*; *TfR1*^f/f^ kidneys (Figure 8J). Immunostaining revealed near complete repopulation of *TfR1-*PT with *TfR1*^+^cells (Figure 8, K–M). These data are in accordance with the notion that iron acquisition through *TfR1* is critical for the postnatal growth of the kidney.

## Discussion

This study depicts the effect of FeD in the developing kidney. The models illustrate growth failure, varying in phenotype according to (*1*) the deficient form of iron transport, (*2*) the response of different kidney segments, and (*3*) the stage of development. Because segment-specific deletions of *TfR1* produced segment-specific cystic-hypodysplasia after birth and because iron therapy reversed the phenotype, we can confirm a direct relationship between FeD, cell proliferation, nephron development, and kidney architecture. Together, the phenotypes may be considered a new entity called iron-deficient kidney disease.

NTBI has been measured in a wide range of iron overload disorders such as hemochromatosis,^79^ thalassemia,^80–82^ blood transfusions,^44^ and inflammatory disorders^83–85^ and is implicated in tissue damage,^86,87^ rather than serving a physiologic function. We inferred a physiologic role for NTBI in kidney growth, because of the development of approximately E11–E12 kidneys in *TfR1*^*−/−*^ mice, the development of *TfR1*^*−/−*^ ES cells in wild-type kidney chimeras, and the development of mesenchymal-epithelial, ureteric, and stromal compartments at midgestation (E15.5), despite *TfR1* deletions by *Six2Cre*, *Hoxb7Cre*, and *FoxD1Cre*, respectively. Past studies have also identified partial organogenesis in *TfR1* deleted mouse embryos,^6^ in hypotransferrinemic mice (*Hpx*)^88^ and fetal humans,^89,90^ and in conditional *TfR1* knockouts,^91–93^ which demonstrated perinatal rather than strong embryonic phenotypes in heart and gastrointestinal system. Together, these data suggest that NTBI iron donation must complement *TfR1* in the early embryo. In fact, two candidate NTBI transporters,^69^ Zip8 and Zip14, were upregulated by TfD (Supplemental Figure 10). Additional NTBI transporters include NRAMP1, NRAMP2,^94–96^ TRPML1 (lysosomal iron transporter),^97^ L-Type, and T-Type calcium channel^98,99^ expressed in S-shaped nephrons.^69^ Hence, our data suggest that multiple iron capture mechanisms are active in early embryo. In this light, FeD produced a more severe phenotype than TfD because it depleted both NTBI and Tf bound iron.

The mild embryonic TfD phenotype might have been mollified not only by NTBI but by residual *TfR1^+^* rogue cells. However, these cells were undetectable in *Six2-Cre* *EGFP*^+^ the progenitor pool, and only scattered *TfR1^+^* cells were found at midgestation in established tubules (Figure 4, A and B). This pattern is consistent with the limited importance of *TfR1* mediated growth in early gestation (Figure 1, A and B).

Although NTBI may have a physiologic role in early gestation, several lines of evidence demonstrate that transferrin becomes the dominant iron carrier after midgestation: (*1*) endogenous *TfR1*^+^ cells replaced *TfR1*^−^ cells in late gestation (Figure 4), (*2*) tubule cell proliferation coincided with *Tfr1* expression (Figure 4), and (*3*) cystic hypodysplasia was apparent >P8 in TfD kidneys (Figures 2 and 3). Perhaps increasing concentrations of circulating apo-transferrin deplete NTBI due to its higher affinity for iron,^100^ explaining the dominance of transferrin emerging in the perinatal period. This shift is likely critical for organ development because transferrin-iron is stable in the oxygenated^24,25^ kidney cortex.^101^ In addition, transferrin can undergo multiple rounds of iron delivery, while NTBI carriers are metabolized (*e.g*., citrate to bicarbonate). Hence, affinity, stability, and the efficiency of transferrin-*TfR1* impart growth advantage.

Murine kidney development continues for several days after birth.^102,103^ At the end of the second postnatal week, components of cell cycle and growth pathways (*e.g*., *TfR1*; Figure 4) are downregulated, while transport genes typical of mature epithelia are upregulated.^104^ Many of these genes were modulated in both of our models, FeD (283 genes *P*adj < 0.001) and TfD (75 genes *P*adj < 0.001) at P13–15. As an example, *Ndrg1* is upregulated by FeD^105^ (1.56-fold in FeD and 2.11-fold in TfD models, *P* < 0.05), particularly in PTs.^106^ *NDRG1* opposes proliferative pathways, implying that it contributes to the transition at P13–15. In this light, transferrin iron–mediated proliferation is required for the timing and success of subsequent tubule maturation. By contrast, the deletion of *TfR1* with *Megalin-CreERT*^43^ 2 weeks after birth does not disrupt kidney morphogenesis, and it failed to generate cystic dysplasia.

The morphogenic failure at the transition point (Figure 2) was reminiscent of the time dependence of polycystic kidney disease and whole ciliary knockouts.^104,107,108^ In both TfD and a few surviving FeD kidneys, the cysts contained longer cilia at P15 and P60 with lower density of ARL13b staining (Supplemental Figure 13), which is inversely proportional to ciliary volume. Moreover, cystic tubules and noncystic neighbors demonstrated elongated cilia, suggesting that ciliary elongation may precede cystogenesis and provide a marker of cellular disruption due to FeD.^109–112^ In fact, suppression of many of the ciliary trafficking adapter proteins, the BBSomes and the nephronophthisis (*Nphp*) genes were common to both *Six2Cre* and KspCre-mediated *TfR1* deletion. Bardet-Biedl syndrome genes were re-expressed on iron replacement (Supplemental Figure 14). Together, these data suggest the possibility that pathways linked to cilia modulate both iron-mediated growth and epithelial morphogenesis.^113^

In contrast to our findings, FeD and TfD did not modulate classical cyst-associated genes, including *Nphp*, *Hnf1b*, *Jbt*, *Pkd1/2,* or *Tsc-vHL* genes. Hence, we examined a second model of developmental failure of medullary and pelvic development, the *Wnt7b* deletion.^114^ However, we did not observe analogous changes in *BBSome* or *Nphp* genes, most likely because *Wnt7b* appears to be signaling to the stroma rather than the epithelia. *Wnt9b* disruption, on the other hand, generated striking images of cystogenesis^115^ similar to TfD, in both appearance and suppression of the same *BBSomes* and *Nphp* genes (*e.g*., *Bbs1/2/6/7/8; Nphp6/7/8*; Supplemental Figure 14). Indeed, analysis of previously published microarray data from P1, *Wnt9b* hypomorphic mutants showed significantly reduced expression of *TfR1* in mutants (fold change −1.53), indicating that Wnt drives *TfR1.* Further analysis with reciprocal knockouts is needed to determine the relationship of iron-deficient kidney disease, Wnt signaling, and many other forms of cystic kidney disease.

Caspase-dependent apoptosis is directly required for lumenal cavitation^116^ and caspase inhibition suppresses cyst formation and disease progression in polycystic kidney disease models.^117,118^ Mechanistically, apoptosis weakens entire segments of the tubular wall, leaving surviving epithelia unable to maintain normal architecture, promoting lumen dilation and cyst initiation.^117^ Suppressed proliferation may enhance the phenotype by limiting repair. Hence, ciliary dysfunction, hypoplasia, and apoptosis contribute to cystic TfD.

The dramatic morphogenic failure at P13–15 suggested that a variety of amplifying loops are at play. For example, the liver is the major site of fetal EPO production, but this function is transferred to the kidney in the first 2 postnatal weeks, coinciding with widespread cystic dysplasia induced by TfD. Perhaps, the suppression kidney EPO^119–122^ (Figure 8) enhanced the P13–15 failure.^123,124^ EPO stimulates the expression of erythroferrone, which suppresses hepcidin antimicrobial peptide (HAMP). Consequently, EPO failure increases HAMP, which in turn suppresses duodenal iron transporters,^125–127^ amplifying TfD FeD.

To test the proposed systemic amplification loop, we treated kidney TfD mice with Roxadustat, an agent that activates HIF1*α* and HIF2*α*, which drive iron trafficking genes.^78^ Roxadustat stimulated EPO production^128^ suppressed HAMP^129,130^ and rescued duodenal *Dcytb.^131^* Roxadustat also induced *TfR1* expression in liver.^75,132^ In turn, Roxadustat normalized kidney iron content and repopulated *Six2Cre*; *TfR1*^*−/−*^ tubules with *TfR1*^+^ rogue cells (Figure 8). Hence, we suspect that kidney TfD disrupts both kidney (through suppressed kidney EPO) and systemic iron delivery (through amplified liver HAMP), amplifying the morphogenic failure at P13–15.

The most prevalent threat to embryonic development is malnutrition.^133,134^ Gestational malnutrition can program long lasting metabolic consequences^135^ including the selection of thrifty genes (Barker Hypothesis) that may explain kidney phenotypes decades after forced starvation (*e.g*., Dutch Hunger Winter Study,^136^ China's Great Famine^137^). These challenges are critical because even mild hypoplasia may presage hypertension and CKD (Brenner hypothesis).^138^ Hence, periconceptual FeD could explain the variation in human nephron numbers,^28–30^ an unstudied mechanism of CKD.

While much is known about kidney development, its regulation by micronutrients is less explored. Perhaps an explanation for iron deficient kidney disease is found in Short *et al.*, Sampogna *et al.*, and Hannezo *et al.*^34,53,139^ who defined sequential modules of mesenchymal, nephron, and ureteric growth and demonstrated that these modules were targets of nutrient-specific deficiencies. The modules include gestational windows between E15.5 and 19.5, which is marked by the rapid elongation of PTs and cortico-medullary collecting ducts, particularly branching generations five through eight (when rapid 2*n* bifurcating events transition into a stochastic pattern of branching morphogenesis).^115,140,141^ The growth modules also include the medullary extension of the Loop of Henle (approximately second postnatal week).^142–144^ Finally, a transition-point between P12 and P14 signals an end of these critical growth windows.^104^ These data are striking because the modules of growth coincide with the timed waves of *TfR1* expression. Hence, transferrin iron determines not only the growth of nephrons but apparently is required for their transition to the mature phenotype (*e.g*., Supplemental Figure 6).

Our data also demonstrate the difficulties of iron repletion because FeD in the embryo and in the neonate may self-amplify because of placental defects and/or HAMP expression, respectively. Although these findings might seem counterintuitive, this mechanism may protect the placenta^27^ or even littermates by reducing iron transfer to nonviable embryos.

It is challenging to translate our data to humans.^145^ Human kidney development terminates *in utero*, making stage-specific iron supplementation much more challenging than in mice. Nonetheless, the dramatic normalization of kidney structure after a single dose of iron suggests the utility of iron administration timed to the most sensitive windows of iron demand. Perhaps enhanced iron delivery by HIF activators could support kidney growth thereby permitting efficient nutritional repletion even with limited iron dosing.^78,146^

In summary, both NTBI and transferrin iron contribute to organogenesis before midgestation, but transferrin iron is a prerequisite for postnatal growth and subsequently for epithelial maturation. TfD defeats this developmental patterning and results in cystic hypodysplasia and kidney failure (Supplemental Figure 15).

## Supplementary Material

## Data Availability

Original data generated for the study are or will be made available in a public access repository upon publication. Data Type: Observational Data. Small scale mouse experiments comparing wild type and knockout mice. Repository Name: GEO. Linkable Citation: RNAseq data were deposited in GEO (access number: GSE100254 (reviewer token mtefiwqyjverlet). Mouse models are available upon request including *TfR1*f/f, HIF1af/f and Cre drivers, Six2EGFPCre, KspCre, HoxB7Cre, Foxd1Cre, Megalin3â€CreERT. Frozen stocks are stored at Jax Mice https://www.jax.org. Data were deposited in GEO https://www.ncbi.nlm.nih.gov/geo/query/acc.cgi?acc=GSE100254 (reviewer token mtefiwqyjverlet).

## Declarative Statements

All animal experiments were conducted in accordance with the NIH Guide for the Care and Use of Laboratory Animals or an equivalent standard that meets or exceeds the ethical and welfare requirements outlined in the NIH Guide. All protocols were approved by the appropriate institutional animal care and use committee.

## Supplemental Material

Supplemental Figure 1. Deletion of *TfR1* with the *Six2Cre*EGFP driver (E15.5) did not block epithelial induction.

Supplemental Figure 2. Deletion of *TfR1* with the *Hoxb7Cre* driver (E12.5) did not block growth of UB.

Supplemental Figure 3. Generation of *TfR1* floxed mice.

Supplemental Figure 4. *TfR1* deletion in UB and Stroma.

Supplemental Figure 5. Apoptosis in the corticomedullary junction and medulla at P13.

Supplemental Figure 6. Gene regulation in TfD and FeD models.

Supplemental Figure 7. Deletion of *TfR1* in the nephron resulted in cystic hypodysplasia after birth.

Supplemental Figure 8. Defective growth of the TALH.

Supplemental Figure 9. PT growth defects, comparison of FeD and TfD.

Supplemental Figure 10. NTBI transporters are upregulated by in TfD.

Supplemental Figure 11. Conditional deletion of *Hif1* exon 2 by *Six2Cre*; *Hif1*^*f/f*^.

Supplemental Figure 12. HIF1*α* targeted genes were enriched in Six2Cre; *Tfr1*^f/f^ mice.

Supplemental Figure 13. Response of the ciliary system to FeD.

Supplemental Figure 14. Downregulation of *BBSomes* genes.

Supplemental Figure 15. Summary diagram.

Supplemental Table 1. Materials.

Supplemental References
