# Supplementary material for: A Switch in Iron Delivery Is Critical for Postnatal Kidney Development
Source: Kidney360. 2026 Feb 17;7(5):982–1002. doi: 10.34067/KID.0000001064 (PMC13229437; doi:10.34067/KID.0000001064)
Supplement: Supplementary file 1 [file kidney360-7-0982-s001.pdf]

ASN American Society of Nephrology
JASN Journal of the American Society of Nephrology
KIDNEY360 Accessing Our World From Every Angle
CJASN Clinical Journal of the American Society of Nephrology

ASN Journal Disclosure Form

As per ASN journal policy, I have disclosed any financial relationships or commitments I have held in the past 36 months as included below. I have listed my Current Employer below to indicate there is a relationship requiring disclosure. If no relationship exists, my Current Employer is not listed.

There is no minimum financial threshold; individuals must disclose all financial relationships, regardless of the amount, with ineligible companies. Individuals must disclose for every category below, regardless of their view of the relevance of the relationship to the activity. (*"Ineligible companies" are those whose primary business is producing, marketing, selling, re-selling, or distributing health care products used by or on patients.*)

Date: 11/25/2025

Author Name: Jonathan Barasch

Manuscript ID: K360-2025-000777R1

Manuscript Title: A Switch in Iron Delivery Is Critical for Postnatal Kidney Development

Disclosure Statement: *(including all categories below*)*

Current Employer(s) & Affiliation(s): Columbia University
Grants / Contracts / Research Funding: The work was supported by grants supported by grants from the National Institute of Health Diabetes and Digestive and Kidney Diseases (DK-55388, DK092684, R56DK092684, DK073462, DK-106548, DK124667/DK, T32 DK108741/DK, UG3 DK114926/DK, U54 DK104309) and and the March of Dimes for Birth Defects. Additional funding to J.B. was provided by the Glomerular Center of Columbia University.
Consultancy / Advisory Board: None; Honoraria / Speaking Fees: None; Equity / Stock Ownership: None; Patents / Royalties: None; Leadership / Board / Editorial Roles: None; Speakers' Bureau: None; Other Interests / Relationships: None

***Categories:** Employer; Ownership Interest; Consultancy; Research Funding; Honoraria; Patents or Royalties; Advisory or Leadership Role; Speakers Bureau; and Other Interests or Relationships

Author Acknowledgment: I understand that the information above will be published within the journal article, if accepted, and that failure to comply and/or to accurately and completely report the potential financial conflicts of interest could lead to the following: 1) Prior to publication, article rejection, or 2) Post-publication, sanctions ranging from, but not limited to, issuing a correction, reporting the inaccurate information to the authors' institution, banning authors from submitting work to ASN journals for varying lengths of time, and/or retraction of the published work.

Author Name and/or Initials: Jonathan Barasch

ASN American Society of Nephrology
JASN Journal of the American Society of Nephrology
KIDNEY360 Accessing Our World From Every Angle
CJASN Clinical Journal of the American Society of Nephrology

ASN Journal Disclosure Form

As per ASN journal policy, I have disclosed any financial relationships or commitments I have held in the past 36 months as included below. I have listed my Current Employer below to indicate there is a relationship requiring disclosure. If no relationship exists, my Current Employer is not listed.

A. Beenken reports the following:
Employer: New York Presbyterian / Columbia University Vagelos College of Physicians and Surgeons

I understand that the information above will be published within the journal article, if accepted, and that failure to comply and/or to accurately and completely report the potential financial conflicts of interest could lead to the following: 1) Prior to publication, article rejection, or 2) Post-publication, sanctions ranging from, but not limited to, issuing a correction, reporting the inaccurate information to the authors' institution, banning authors from submitting work to ASN journals for varying lengths of time, and/or retraction of the published work.

Name: Andrew Samuel Beenken
Manuscript ID: K360-2025-000777R1
Manuscript Title: A Switch in Iron Delivery Is Critical for Postnatal Kidney Development
Date of Completion: October 16, 2025
Disclosure Updated Date: October 16, 2025

ASN Journal Disclosure Form

As per ASN journal policy, I have disclosed any financial relationships or commitments I have held in the past 36 months as included below. I have listed my Current Employer below to indicate there is a relationship requiring disclosure. If no relationship exists, my Current Employer is not listed.

There is no minimum financial threshold; individuals must disclose all financial relationships, regardless of the amount, with ineligible companies. Individuals must disclose for every category below, regardless of their view of the relevance of the relationship to the activity. ("*Ineligible companies*" *are those whose primary business is producing, marketing, selling, re-selling, or distributing health care products used by or on patients.*)

Date: 11/25/2025

Author Name: Roger Wilson Boles

Manuscript ID: K360-2025-000777R1

Manuscript Title: A Switch in Iron Delivery Is Critical for Postnatal Kidney Development

Disclosure Statement: *(including all categories below*)*

Current Employer(s) & Affiliation(s): University of Southern California, Los Angeles General Medical Center
Grants / Contracts / Research Funding: none
Consultancy / Advisory Board: none
Honoraria / Speaking Fees: none
Equity / Stock Ownership: none
Patents / Royalties: none
Leadership / Board / Editorial Roles: none
Speakers' Bureau: none
Other Interests / Relationships: none
Spouse / Partner Interests: none

***Categories:** Employer; Ownership Interest; Consultancy; Research Funding; Honoraria; Patents or Royalties; Advisory or Leadership Role; Speakers Bureau; and Other Interests or Relationships

Author Acknowledgment: I understand that the information above will be published within the journal article, if accepted, and that failure to comply and/or to accurately and completely report the potential financial conflicts of interest could lead to the following: 1) Prior to publication, article rejection, or 2) Post-publication, sanctions ranging from, but not limited to, issuing a correction, reporting the inaccurate information to the authors' institution, banning authors from submitting work to ASN journals for varying lengths of time, and/or retraction of the published work.

Author Name and/or Initials: Roger Wilson Boles

ASN
American Society of Nephrology
JASN
Journal of the American Society of Nephrology
KIDNEY360
Accessing Our World From Every Angle
CJASN
Clinical Journal of the American Society of Nephrology

ASN Journal Disclosure Form

As per ASN journal policy, I have disclosed any financial relationships or commitments I have held in the past 36 months as included below. I have listed my Current Employer below to indicate there is a relationship requiring disclosure. If no relationship exists, my Current Employer is not listed.

There is no minimum financial threshold; individuals must disclose all financial relationships, regardless of the amount, with ineligible companies. Individuals must disclose for every category below, regardless of their view of the relevance of the relationship to the activity. ("*Ineligible companies*" *are those whose primary business is producing, marketing, selling, re-selling, or distributing health care products used by or on patients.*)

Date: 11/25/2025

Author Name: Efrat Bruck, MD

Manuscript ID: K360-2025-000777R1

Manuscript Title: A Switch in Iron Delivery Is Critical for Postnatal Kidney Development

Disclosure Statement: *(including all categories below*)*

Current Employer(s) & Affiliation(s): G2 Anesthesia, Los Gatos, CA
Grants / Contracts / Research Funding: none
Consultancy / Advisory Board: none
Honoraria / Speaking Fees: none
Equity / Stock Ownership: none
Patents / Royalties: none
Leadership / Board / Editorial Roles: none
Speakers' Bureau: none
Other Interests / Relationships: none
Spouse / Partner Interests: none

***Categories:** Employer; Ownership Interest; Consultancy; Research Funding; Honoraria; Patents or Royalties; Advisory or Leadership Role; Speakers Bureau; and Other Interests or Relationships

Author Acknowledgment: I understand that the information above will be published within the journal article, if accepted, and that failure to comply and/or to accurately and completely report the potential financial conflicts of interest could lead to the following: 1) Prior to publication, article rejection, or 2) Post-publication, sanctions ranging from, but not limited to, issuing a correction, reporting the inaccurate information to the authors' institution, banning authors from submitting work to ASN journals for varying lengths of time, and/or retraction of the published work.

Author Name and/or Initials: Efrat Bruck 10/15/2025

ASN
American Society of Nephrology
JASN
Journal of the American Society of Nephrology
KIDNEY360
Accessing Our World From Every Angle
CJASN
Clinical Journal of the American Society of Nephrology

ASN Journal Disclosure Form

As per ASN journal policy, I have disclosed any financial relationships or commitments I have held in the past 36 months as included below. I have listed my Current Employer below to indicate there is a relationship requiring disclosure. If no relationship exists, my Current Employer is not listed.

T. Carroll reports the following:
Employer: UTSW Medical Center

I understand that the information above will be published within the journal article, if accepted, and that failure to comply and/or to accurately and completely report the potential financial conflicts of interest could lead to the following: 1) Prior to publication, article rejection, or 2) Post-publication, sanctions ranging from, but not limited to, issuing a correction, reporting the inaccurate information to the authors' institution, banning authors from submitting work to ASN journals for varying lengths of time, and/or retraction of the published work.

Name: Thomas J. Carroll
Manuscript ID: K360-2025-000777R1
Manuscript Title: A Switch in Iron Delivery Is Critical for Postnatal Kidney Development
Date of Completion: October 16, 2025
Disclosure Updated Date: October 16, 2025

ASN American Society of Nephrology | JASN Journal of the American Society of Nephrology | KIDNEY360 Accessing Our World From Every Angle | CJASN Clinical Journal of the American Society of Nephrology

ASN Journal Disclosure Form

As per ASN journal policy, I have disclosed any financial relationships or commitments I have held in the past 36 months as included below. I have listed my Current Employer below to indicate there is a relationship requiring disclosure. If no relationship exists, my Current Employer is not listed.

There is no minimum financial threshold; individuals must disclose all financial relationships, regardless of the amount, with ineligible companies. Individuals must disclose for every category below, regardless of their view of the relevance of the relationship to the activity. ("*Ineligible companies" are those whose primary business is producing, marketing, selling, re-selling, or distributing health care products used by or on patients.*)

Date: 11/25/2025

Author Name: Kaitlyn Corbin

Manuscript ID: K360-2025-000777R1

Manuscript Title: A Switch in Iron Delivery Is Critical for Postnatal Kidney Development

Disclosure Statement: *(including all categories below*)*

Current Employer(s) & Affiliation(s): Accenture LLP
Grants / Contracts / Research Funding: none
Consultancy / Advisory Board: Accenture, Employee - Unrelated Field
Honoraria / Speaking Fees: none
Equity / Stock Ownership: none
Patents / Royalties: none
Leadership / Board / Editorial Roles: none
Speakers' Bureau: none
Other Interests / Relationships: none
Spouse / Partner Interests: none

***Categories:** Employer; Ownership Interest; Consultancy; Research Funding; Honoraria; Patents or Royalties; Advisory or Leadership Role; Speakers Bureau; and Other Interests or Relationships

Author Acknowledgment: I understand that the information above will be published within the journal article, if accepted, and that failure to comply and/or to accurately and completely report the potential financial conflicts of interest could lead to the following: 1) Prior to publication, article rejection, or 2) Post-publication, sanctions ranging from, but not limited to, issuing a correction, reporting the inaccurate information to the authors' institution, banning authors from submitting work to ASN journals for varying lengths of time, and/or retraction of the published work.

Author Name and/or Initials: Kaitlyn Corbin

ASN American Society of Nephrology | JASN Journal of the American Society of Nephrology | KIDNEY360 Accessing Our World From Every Angle | CJASN Clinical Journal of the American Society of Nephrology

ASN Journal Disclosure Form

As per ASN journal policy, I have disclosed any financial relationships or commitments I have held in the past 36 months as included below. I have listed my Current Employer below to indicate there is a relationship requiring disclosure. If no relationship exists, my Current Employer is not listed.

There is no minimum financial threshold; individuals must disclose all financial relationships, regardless of the amount, with ineligible companies. Individuals must disclose for every category below, regardless of their view of the relevance of the relationship to the activity. ("*Ineligible companies" are those whose primary business is producing, marketing, selling, re-selling, or distributing health care products used by or on patients.*)

Date: 11/25/2025

Author Name: Beatriz Desanti de Oliveira

Manuscript ID: K360-2025-000777R1

Manuscript Title: A Switch in Iron Delivery Is Critical for Postnatal Kidney Development

Disclosure Statement: *(including all categories below*)*

Current Employer(s) & Affiliation(s): New York Presbyterian-Columbia University
Grants / Contracts / Research Funding: none
Consultancy / Advisory Board: none
Honoraria / Speaking Fees: none
Equity / Stock Ownership: none
Patents / Royalties: none
Leadership / Board / Editorial Roles: none
Speakers' Bureau: none
Other Interests / Relationships: none

***Categories:** Employer; Ownership Interest; Consultancy; Research Funding; Honoraria; Patents or Royalties; Advisory or Leadership Role; Speakers Bureau; and Other Interests or Relationships

Author Acknowledgment: I understand that the information above will be published within the journal article, if accepted, and that failure to comply and/or to accurately and completely report the potential financial conflicts of interest could lead to the following: 1) Prior to publication, article rejection, or 2) Post-publication, sanctions ranging from, but not limited to, issuing a correction, reporting the inaccurate information to the authors' institution, banning authors from submitting work to ASN journals for varying lengths of time, and/or retraction of the published work.

Author Name and/or Initials: Beatriz Desanti de Oliveira

ASN Journal Disclosure Form

As per ASN journal policy, I have disclosed any financial relationships or commitments I have held in the past 36 months as included below. I have listed my Current Employer below to indicate there is a relationship requiring disclosure. If no relationship exists, my Current Employer is not listed.

R. Deng reports the following:
Employer: Columbia University

I understand that the information above will be published within the journal article, if accepted, and that failure to comply and/or to accurately and completely report the potential financial conflicts of interest could lead to the following: 1) Prior to publication, article rejection, or 2) Post-publication, sanctions ranging from, but not limited to, issuing a correction, reporting the inaccurate information to the authors' institution, banning authors from submitting work to ASN journals for varying lengths of time, and/or retraction of the published work.

Name: Rongjia Deng
Manuscript ID: K360-2025-000777R1
Manuscript Title: A Switch in Iron Delivery Is Critical for Postnatal Kidney Development
Date of Completion: October 19, 2025
Disclosure Updated Date: October 19, 2025

ASN American Society of Nephrology
JASN Journal of the American Society of Nephrology
KIDNEY360 Accessing Our World From Every Angle
CJASN Clinical Journal of the American Society of Nephrology

ASN Journal Disclosure Form

As per ASN journal policy, I have disclosed any financial relationships or commitments I have held in the past 36 months as included below. I have listed my Current Employer below to indicate there is a relationship requiring disclosure. If no relationship exists, my Current Employer is not listed.

L. Dionne reports the following:
Employer: Washington University in St Louis

I understand that the information above will be published within the journal article, if accepted, and that failure to comply and/or to accurately and completely report the potential financial conflicts of interest could lead to the following: 1) Prior to publication, article rejection, or 2) Post-publication, sanctions ranging from, but not limited to, issuing a correction, reporting the inaccurate information to the authors' institution, banning authors from submitting work to ASN journals for varying lengths of time, and/or retraction of the published work.

Name: Lai Kuan Dionne
Manuscript ID: K360-2025-000777R1
Manuscript Title: A Switch in Iron Delivery Is Critical for Postnatal Kidney Development
Date of Completion: November 25, 2025
Disclosure Updated Date: November 25, 2025

ASN American Society of Nephrology
JASN Journal of the American Society of Nephrology
KIDNEY360 Accessing Our World From Every Angle
CJASN Clinical Journal of the American Society of Nephrology

ASN Journal Disclosure Form

As per ASN journal policy, I have disclosed any financial relationships or commitments I have held in the past 36 months as included below. I have listed my Current Employer below to indicate there is a relationship requiring disclosure. If no relationship exists, my Current Employer is not listed.

There is no minimum financial threshold; individuals must disclose all financial relationships, regardless of the amount, with ineligible companies. Individuals must disclose for every category below, regardless of their view of the relevance of the relationship to the activity. ("*Ineligible companies" are those whose primary business is producing, marketing, selling, re-selling, or distributing health care products used by or on patients.*)

Date: 11/25/2025

Author Name: Aryan Ghotra

Manuscript ID: K360-2025-000777R1

Manuscript Title: A Switch in Iron Delivery Is Critical for Postnatal Kidney Development

Disclosure Statement: *(including all categories below*)*

Current Employer(s) & Affiliation(s): Stony Brook University
Grants / Contracts / Research Funding: None
Consultancy / Advisory Board: None
Honoraria / Speaking Fees: None
Equity / Stock Ownership: None
Patents / Royalties: None
Leadership / Board / Editorial Roles: None
Speakers' Bureau: None
Other Interests / Relationships: None
Spouse / Partner Interests: None

***Categories:** Employer; Ownership Interest; Consultancy; Research Funding; Honoraria; Patents or Royalties; Advisory or Leadership Role; Speakers Bureau; and Other Interests or Relationships

Author Acknowledgment: I understand that the information above will be published within the journal article, if accepted, and that failure to comply and/or to accurately and completely report the potential financial conflicts of interest could lead to the following: 1) Prior to publication, article rejection, or 2) Post-publication, sanctions ranging from, but not limited to, issuing a correction, reporting the inaccurate information to the authors' institution, banning authors from submitting work to ASN journals for varying lengths of time, and/or retraction of the published work.

Author Name and/or Initials: Aryan Ghotra

ASN American Society of Nephrology JASN Journal of the American Society of Nephrology KIDNEY360 Accessing Our World From Every Angle CJASN Clinical Journal of the American Society of Nephrology

ASN Journal Disclosure Form

As per ASN journal policy, I have disclosed any financial relationships or commitments I have held in the past 36 months as included below. I have listed my Current Employer below to indicate there is a relationship requiring disclosure. If no relationship exists, my Current Employer is not listed.

C. Hinze reports the following:
Employer: Medizinische Hochschule Hannover; and Consultancy: AstraZeneca, Chiesi, Amgen, Boehringer-Ingelheim, Stada, Amgen, Biogen.

I understand that the information above will be published within the journal article, if accepted, and that failure to comply and/or to accurately and completely report the potential financial conflicts of interest could lead to the following: 1) Prior to publication, article rejection, or 2) Post-publication, sanctions ranging from, but not limited to, issuing a correction, reporting the inaccurate information to the authors' institution, banning authors from submitting work to ASN journals for varying lengths of time, and/or retraction of the published work.

Name: Christian Hinze
Manuscript ID: K360-2025-000777R1
Manuscript Title: A Switch in Iron Delivery Is Critical for Postnatal Kidney Development
Date of Completion: November 26, 2025
Disclosure Updated Date: November 26, 2025

ASN American Society of Nephrology | JASN Journal of the American Society of Nephrology | KIDNEY360 Accessing Our World From Every Angle | CJASN Clinical Journal of the American Society of Nephrology

ASN Journal Disclosure Form

As per ASN journal policy, I have disclosed any financial relationships or commitments I have held in the past 36 months as included below. I have listed my Current Employer below to indicate there is a relationship requiring disclosure. If no relationship exists, my Current Employer is not listed.

A. Khan reports the following:
Employer: Columbia University

I understand that the information above will be published within the journal article, if accepted, and that failure to comply and/or to accurately and completely report the potential financial conflicts of interest could lead to the following: 1) Prior to publication, article rejection, or 2) Post-publication, sanctions ranging from, but not limited to, issuing a correction, reporting the inaccurate information to the authors' institution, banning authors from submitting work to ASN journals for varying lengths of time, and/or retraction of the published work.

Name: Atlas Khan
Manuscript ID: K360-2025-000777R1
Manuscript Title: A Switch in Iron Delivery Is Critical for Postnatal Kidney Development
Date of Completion: November 26, 2025
Disclosure Updated Date: January 6, 2025

ASN
American Society of Nephrology
JASN
Journal of the American Society of Nephrology
KIDNEY360
Accessing Our World From Every Angle
CJASN
Clinical Journal of the American Society of Nephrology

ASN Journal Disclosure Form

As per ASN journal policy, I have disclosed any financial relationships or commitments I have held in the past 36 months as included below. I have listed my Current Employer below to indicate there is a relationship requiring disclosure. If no relationship exists, my Current Employer is not listed.

There is no minimum financial threshold; individuals must disclose all financial relationships, regardless of the amount, with ineligible companies. Individuals must disclose for every category below, regardless of their view of the relevance of the relationship to the activity. ("*Ineligible companies" are those whose primary business is producing, marketing, selling, re-selling, or distributing health care products used by or on patients.*)

Date: 11/25/2025

Author Name: Abraham Daniel Levitman

Manuscript ID: K360-2025-000777R1

Manuscript Title: A Switch in Iron Delivery Is Critical for Postnatal Kidney Development

Disclosure Statement: *(including all categories below*)*

Current Employer(s) & Affiliation(s): Albert Einstein College of Medicine/Montefiore Medical Center, Department of Surgery
Grants / Contracts / Research Funding: none
Consultancy / Advisory Board: none
Honoraria / Speaking Fees: none
Equity / Stock Ownership: none
Patents / Royalties: none
Leadership / Board / Editorial Roles: none
Speakers' Bureau: none
Other Interests / Relationships: none

***Categories:** Employer; Ownership Interest; Consultancy; Research Funding; Honoraria; Patents or Royalties; Advisory or Leadership Role; Speakers Bureau; and Other Interests or Relationships

Author Acknowledgment: I understand that the information above will be published within the journal article, if accepted, and that failure to comply and/or to accurately and completely report the potential financial conflicts of interest could lead to the following: 1) Prior to publication, article rejection, or 2) Post-publication, sanctions ranging from, but not limited to, issuing a correction, reporting the inaccurate information to the authors' institution, banning authors from submitting work to ASN journals for varying lengths of time, and/or retraction of the published work.

Author Name and/or Initials: Abraham Daniel Levitman

ASN American Society of Nephrology · JASN Journal of the American Society of Nephrology · KIDNEY360 Accessing Our World From Every Angle · CJASN Clinical Journal of the American Society of Nephrology

ASN Journal Disclosure Form

As per ASN journal policy, I have disclosed any financial relationships or commitments I have held in the past 36 months as included below. I have listed my Current Employer below to indicate there is a relationship requiring disclosure. If no relationship exists, my Current Employer is not listed.

There is no minimum financial threshold; individuals must disclose all financial relationships, regardless of the amount, with ineligible companies. Individuals must disclose for every category below, regardless of their view of the relevance of the relationship to the activity. ("*Ineligible companies" are those whose primary business is producing, marketing, selling, re-selling, or distributing health care products used by or on patients.*)

Date: 11/25/2025

Author Name: Moe R Mahjoub

Manuscript ID: K360-2025-000777R1

Manuscript Title: A Switch in Iron Delivery Is Critical for Postnatal Kidney Development

Disclosure Statement: *(including all categories below*)*

Current Employer(s) & Affiliation(s): Washington University in St Louis
Grants / Contracts / Research Funding: Experiments performed by the Mahjoub lab for this manuscript were supported by funding from NIDDK (R01-DK108005)
Consultancy / Advisory Board: none
Honoraria / Speaking Fees: none
Equity / Stock Ownership: none
Patents / Royalties: none
Leadership / Board / Editorial Roles: none
Speakers' Bureau: none
Other Interests / Relationships: none
Spouse / Partner Interests: none

***Categories:** Employer; Ownership Interest; Consultancy; Research Funding; Honoraria; Patents or Royalties; Advisory or Leadership Role; Speakers Bureau; and Other Interests or Relationships

Author Acknowledgment: I understand that the information above will be published within the journal article, if accepted, and that failure to comply and/or to accurately and completely report the potential financial conflicts of interest could lead to the following: 1) Prior to publication, article rejection, or 2) Post-publication, sanctions ranging from, but not limited to, issuing a correction, reporting the inaccurate information to the authors' institution, banning authors from submitting work to ASN journals for varying lengths of time, and/or retraction of the published work.

Author Name and/or Initials: Moe R Mahjoub

ASN American Society of Nephrology JASN Journal of the American Society of Nephrology KIDNEY360 Accessing Our World From Every Angle CJASN Clinical Journal of the American Society of Nephrology

ASN Journal Disclosure Form

As per ASN journal policy, I have disclosed any financial relationships or commitments I have held in the past 36 months as included below. I have listed my Current Employer below to indicate there is a relationship requiring disclosure. If no relationship exists, my Current Employer is not listed.

There is no minimum financial threshold; individuals must disclose all financial relationships, regardless of the amount, with ineligible companies. Individuals must disclose for every category below, regardless of their view of the relevance of the relationship to the activity. ("*Ineligible companies*" *are those whose primary business is producing, marketing, selling, re-selling, or distributing health care products used by or on patients.*)

Date: 11/29/2025

Author Name: Kristen McNierney

Manuscript ID: K360-2025-000777R1

Manuscript Title: A Switch in Iron Delivery Is Critical for Postnatal Kidney Development

Disclosure Statement: *(including all categories below*)*

Current Employer(s) & Affiliation(s): Stripe
Grants / Contracts / Research Funding: none
Consultancy / Advisory Board: none
Honoraria / Speaking Fees: none
Equity / Stock Ownership: none
Patents / Royalties: none
Leadership / Board / Editorial Roles: none
Speakers' Bureau: none
Other Interests / Relationships: none
Spouse / Partner Interests: none

***Categories:** Employer; Ownership Interest; Consultancy; Research Funding; Honoraria; Patents or Royalties; Advisory or Leadership Role; Speakers Bureau; and Other Interests or Relationships

Author Acknowledgment: I understand that the information above will be published within the journal article, if accepted, and that failure to comply and/or to accurately and completely report the potential financial conflicts of interest could lead to the following: 1) Prior to publication, article rejection, or 2) Post-publication, sanctions ranging from, but not limited to, issuing a correction, reporting the inaccurate information to the authors' institution, banning authors from submitting work to ASN journals for varying lengths of time, and/or retraction of the published work.

Author Name and/or Initials: Kristen McNierney

ASN
American Society of Nephrology
JASN
Journal of the American Society of Nephrology
KIDNEY360
Accessing Our World From Every Angle
CJASN
Clinical Journal of the American Society of Nephrology

ASN Journal Disclosure Form

As per ASN journal policy, I have disclosed any financial relationships or commitments I have held in the past 36 months as included below. I have listed my Current Employer below to indicate there is a relationship requiring disclosure. If no relationship exists, my Current Employer is not listed.

N. Paragas reports the following:
Employer: University of Washington; Ownership Interest: InVivo Analytics, Inc.; Patents or Royalties: InVivo Analytics, Inc.; and Advisory or Leadership Role: InVivo Analytics, Inc.

I understand that the information above will be published within the journal article, if accepted, and that failure to comply and/or to accurately and completely report the potential financial conflicts of interest could lead to the following: 1) Prior to publication, article rejection, or 2) Post-publication, sanctions ranging from, but not limited to, issuing a correction, reporting the inaccurate information to the authors' institution, banning authors from submitting work to ASN journals for varying lengths of time, and/or retraction of the published work.

Name: Neal Paragas
Manuscript ID: K360-2025-000777R1
Manuscript Title: A Switch in Iron Delivery Is Critical for Postnatal Kidney Development
Date of Completion: December 2, 2025
Disclosure Updated Date: December 2, 2025

ASN American Society of Nephrology JASN Journal of the American Society of Nephrology KIDNEY360 Accessing Our World From Every Angle CJASN Clinical Journal of the American Society of Nephrology

ASN Journal Disclosure Form

As per ASN journal policy, I have disclosed any financial relationships or commitments I have held in the past 36 months as included below. I have listed my Current Employer below to indicate there is a relationship requiring disclosure. If no relationship exists, my Current Employer is not listed.

There is no minimum financial threshold; individuals must disclose all financial relationships, regardless of the amount, with ineligible companies. Individuals must disclose for every category below, regardless of their view of the relevance of the relationship to the activity. ("*Ineligible companies" are those whose primary business is producing, marketing, selling, re-selling, or distributing health care products used by or on patients.*)

Date: 11/25/2025

Author Name: Andong Qiu

Manuscript ID: K360-2025-000777R1

Manuscript Title: A Switch in Iron Delivery Is Critical for Postnatal Kidney Development

Disclosure Statement: *(including all categories below*)*

Current Employer(s) & Affiliation(s): Tongji University
Grants / Contracts / Research Funding: None
Consultancy / Advisory Board: None
Honoraria / Speaking Fees: None
Equity / Stock Ownership: None
Patents / Royalties: None
Leadership / Board / Editorial Roles: None
Speakers' Bureau: None
Other Interests / Relationships: None
Spouse / Partner Interests: None

***Categories:** Employer; Ownership Interest; Consultancy; Research Funding; Honoraria; Patents or Royalties; Advisory or Leadership Role; Speakers Bureau; and Other Interests or Relationships

Author Acknowledgment: I understand that the information above will be published within the journal article, if accepted, and that failure to comply and/or to accurately and completely report the potential financial conflicts of interest could lead to the following: 1) Prior to publication, article rejection, or 2) Post-publication, sanctions ranging from, but not limited to, issuing a correction, reporting the inaccurate information to the authors' institution, banning authors from submitting work to ASN journals for varying lengths of time, and/or retraction of the published work.

Author Name and/or Initials: Andong Qiu

ASN American Society of Nephrology | JASN Journal of the American Society of Nephrology | KIDNEY360 Accessing Our World From Every Angle | CJASN Clinical Journal of the American Society of Nephrology

ASN Journal Disclosure Form

As per ASN journal policy, I have disclosed any financial relationships or commitments I have held in the past 36 months as included below. I have listed my Current Employer below to indicate there is a relationship requiring disclosure. If no relationship exists, my Current Employer is not listed.

There is no minimum financial threshold; individuals must disclose all financial relationships, regardless of the amount, with ineligible companies. Individuals must disclose for every category below, regardless of their view of the relevance of the relationship to the activity. ("*Ineligible companies*" *are those whose primary business is producing, marketing, selling, re-selling, or distributing health care products used by or on patients.*)

Date: 10/29/2025

Author Name: Christian Rosenberger

Manuscript ID: K360-2025-000777R1

Manuscript Title: A Switch in Iron Delivery Is Critical for Postnatal Kidney Development

Disclosure Statement: *(including all categories below*)*

Current Employer(s) & Affiliation(s): Kuratorium fur Heimdialyse, Nordhauser Str. 74
94089 Erfurt, Germany
Grants / Contracts / Research Funding: none
Consultancy / Advisory Board: Astra Zeneca, GSK, Medice
Honoraria / Speaking Fees: Astra Zeneca, GSK, Medice
Equity / Stock Ownership: none
Patents / Royalties: none
Leadership / Board / Editorial Roles: none
Speakers' Bureau: none
Other Interests / Relationships: none
Spouse / Partner Interests: none

***Categories:** Employer; Ownership Interest; Consultancy; Research Funding; Honoraria; Patents or Royalties; Advisory or Leadership Role; Speakers Bureau; and Other Interests or Relationships

Author Acknowledgment: I understand that the information above will be published within the journal article, if accepted, and that failure to comply and/or to accurately and completely report the potential financial conflicts of interest could lead to the following: 1) Prior to publication, article rejection, or 2) Post-publication, sanctions ranging from, but not limited to, issuing a correction, reporting the inaccurate information to the authors' institution, banning authors from submitting work to ASN journals for varying lengths of time, and/or retraction of the published work.

Author Name and/or Initials: Christian Rosenberger

ASN American Society of Nephrology JASN Journal of the American Society of Nephrology KIDNEY360 Accessing Our World From Every Angle CJASN Clinical Journal of the American Society of Nephrology

ASN Journal Disclosure Form

As per ASN journal policy, I have disclosed any financial relationships or commitments I have held in the past 36 months as included below. I have listed my Current Employer below to indicate there is a relationship requiring disclosure. If no relationship exists, my Current Employer is not listed.

R. Sampogna has nothing to disclose.

I understand that the information above will be published within the journal article, if accepted, and that failure to comply and/or to accurately and completely report the potential financial conflicts of interest could lead to the following: 1) Prior to publication, article rejection, or 2) Post-publication, sanctions ranging from, but not limited to, issuing a correction, reporting the inaccurate information to the authors' institution, banning authors from submitting work to ASN journals for varying lengths of time, and/or retraction of the published work.

Name: Rosemary V. Sampogna
Manuscript ID: K360-2025-000777R1
Manuscript Title: A Switch in Iron Delivery Is Critical for Postnatal Kidney Development
Date of Completion: November 25, 2025
Disclosure Updated Date: November 25, 2025

ASN American Society of Nephrology | JASN Journal of the American Society of Nephrology | KIDNEY360 Accessing Our World From Every Angle | CJASN Clinical Journal of the American Society of Nephrology

ASN Journal Disclosure Form

As per ASN journal policy, I have disclosed any financial relationships or commitments I have held in the past 36 months as included below. I have listed my Current Employer below to indicate there is a relationship requiring disclosure. If no relationship exists, my Current Employer is not listed.

There is no minimum financial threshold; individuals must disclose all financial relationships, regardless of the amount, with ineligible companies. Individuals must disclose for every category below, regardless of their view of the relevance of the relationship to the activity. ("*Ineligible companies" are those whose primary business is producing, marketing, selling, re-selling, or distributing health care products used by or on patients.*)

Date: 11/25/2025

Author Name: Prof. Dr. med. Kai Schmidt-Ott, M.D.

Manuscript ID: K360-2025-000777R1

Manuscript Title: A Switch in Iron Delivery Is Critical for Postnatal Kidney Development

Disclosure Statement: *(including all categories below*)*

Current Employer(s) & Affiliation(s): Hannover Medical School, Hannover, Germany
Funding: Kai M. Schmidt-Ott (KMSO) reports receiving research funding from Deutsche Forschungsgemeinschaft (CRC 1365, GRK 2318, RU 2841), Urological Research Foundation Berlin, ERA PerMed (OnAKI-ICI), COVID-19-Forschungsnetzwerk Niedersachsen (EXTINCT post COVID), Niedersächsisches Ministerium für Wissenschaft und Kultur, FAST BioMedical, Quark Pharmaceuticals, REATA, Novartis, Boehringer Ingelheim, AstraZeneca, Sanofi, Apellis, Alentis, Roche, Alexion, CSL Behring;
Consultancy: BioPorto Diagnostics, Alexion, Astellas, AstraZeneca; Bayer, Stadapharm, Boehringer Ingelheim, AstraZeneca;
Honoraria: Boehringer Ingelheim, Astrazeneca, Bayer, Vifor, Alexion, Stadapharm, Novartis, StreamedUp;

Additional Disclosures on second page.

***Categories:** Employer; Ownership Interest; Consultancy; Research Funding; Honoraria; Patents or Royalties; Advisory or Leadership Role; Speakers Bureau; and Other Interests or Relationships

Author Acknowledgment: I understand that the information above will be published within the journal article, if accepted, and that failure to comply and/or to accurately and completely report the potential financial conflicts of interest could lead to the following: 1) Prior to publication, article rejection, or 2) Post-publication, sanctions ranging from, but not limited to, issuing a correction, reporting the inaccurate information to the authors' institution, banning authors from submitting work to ASN journals for varying lengths of time, and/or retraction of the published work.

Author Name and/or Initials: Prof. Dr. med. Kai Schmidt-Ott, M.D.

Disclosures Continued.

Patents / Royalties:

1. New composition comprises compounds, e.g. stem cell factor, cytokine like factor-1, or cardiotrophin-like cytokine, useful for modulating growth of metanephric tissue and for treating damaged kidney tissue. Patent Number: US2008090765-A1. Publ. Date: 04/17/2008; Inventors: Kai M. Schmidt-Ott, Jonathan Barasch, Jun Yang (via Columbia University)

2. Determining the temporal phase of acute kidney injury involves obtaining test sample from subject, and determining the expression level of at least one biomarker e.g. cation transport regulator-like protein 1. Patent Number: WO2011157828-A1; EP2582840-A1; US2013165338- A1. Publ. Date: 12/22/2011. Inventors: Kai M. Schmidt-Ott, Anne Wuebken (via Max Delbrueck Center)

3. Diagnosing acute kidney injury in a subject comprises obtaining a urine sample and determining amount of neutrophil gelatinase-associated lipocalin- and kidney injury molecule-protein; Patent Number(s): WO2012068545-A1. Publ. Date: 05/24/2012. Inventors: Jonathan Barasch, Thomas L. Nickolas, Kai M. Schmidt-Ott (via Columbia University)

Leadership / Board / Editorial Roles: editorial board member of Kidney International, Kidney International Reports, Die Innere Medizin Springer Verlag.

Other Interests / Relationships: license revenue related to the use of a neutrophil gelatinase-associated lipocalin assay via Columbia University;

Spouse / Partner Interests: Regeneron GmbH

ASN American Society of Nephrology
JASN Journal of the American Society of Nephrology
KIDNEY360 Accessing Our World From Every Angle
CJASN Clinical Journal of the American Society of Nephrology

ASN Journal Disclosure Form

As per ASN journal policy, I have disclosed any financial relationships or commitments I have held in the past 36 months as included below. I have listed my Current Employer below to indicate there is a relationship requiring disclosure. If no relationship exists, my Current Employer is not listed.

There is no minimum financial threshold; individuals must disclose all financial relationships, regardless of the amount, with ineligible companies. Individuals must disclose for every category below, regardless of their view of the relevance of the relationship to the activity. ("*Ineligible companies" are those whose primary business is producing, marketing, selling, re-selling, or distributing health care products used by or on patients.*)

Date: 11/25/2025

Author Name: Tian H. Shen

Manuscript ID: K360-2025-000777R1

Manuscript Title: A Switch in Iron Delivery Is Critical for Postnatal Kidney Development

Disclosure Statement: *(including all categories below*)*

Current Employer(s) & Affiliation(s): Columbia University
Grants / Contracts / Research Funding: none
Consultancy / Advisory Board: none
Honoraria / Speaking Fees: none
Equity / Stock Ownership: none
Patents / Royalties: none
Leadership / Board / Editorial Roles: none
Speakers' Bureau: none
Other Interests / Relationships: none

***Categories:** Employer; Ownership Interest; Consultancy; Research Funding; Honoraria; Patents or Royalties; Advisory or Leadership Role; Speakers Bureau; and Other Interests or Relationships

Author Acknowledgment: I understand that the information above will be published within the journal article, if accepted, and that failure to comply and/or to accurately and completely report the potential financial conflicts of interest could lead to the following: 1) Prior to publication, article rejection, or 2) Post-publication, sanctions ranging from, but not limited to, issuing a correction, reporting the inaccurate information to the authors' institution, banning authors from submitting work to ASN journals for varying lengths of time, and/or retraction of the published work.

Author Name and/or Initials: Tian H. Shen

ASN
American Society of Nephrology
JASN
Journal of the American Society of Nephrology
KIDNEY360
Accessing Our World From Every Angle
CJASN
Clinical Journal of the American Society of Nephrology

ASN Journal Disclosure Form

As per ASN journal policy, I have disclosed any financial relationships or commitments I have held in the past 36 months as included below. I have listed my Current Employer below to indicate there is a relationship requiring disclosure. If no relationship exists, my Current Employer is not listed.

There is no minimum financial threshold; individuals must disclose all financial relationships, regardless of the amount, with ineligible companies. Individuals must disclose for every category below, regardless of their view of the relevance of the relationship to the activity. ("*Ineligible companies*" *are those whose primary business is producing, marketing, selling, re-selling, or distributing health care products used by or on patients.*)

Date: 11/25/2025

Author Name: Jacob Stauber

Manuscript ID: K360-2025-000777R1

Manuscript Title: A Switch in Iron Delivery Is Critical for Postnatal Kidney Development

Disclosure Statement: *(including all categories below*)*

Current Employer(s) & Affiliation(s): Albert Einstein College of Medicine
Grants / Contracts / Research Funding: None
Consultancy / Advisory Board: None
Honoraria / Speaking Fees: None
Equity / Stock Ownership: None
Patents / Royalties: None
Leadership / Board / Editorial Roles: None
Speakers' Bureau: None
Other Interests / Relationships: None

***Categories:** Employer; Ownership Interest; Consultancy; Research Funding; Honoraria; Patents or Royalties; Advisory or Leadership Role; Speakers Bureau; and Other Interests or Relationships

Author Acknowledgment: I understand that the information above will be published within the journal article, if accepted, and that failure to comply and/or to accurately and completely report the potential financial conflicts of interest could lead to the following: 1) Prior to publication, article rejection, or 2) Post-publication, sanctions ranging from, but not limited to, issuing a correction, reporting the inaccurate information to the authors' institution, banning authors from submitting work to ASN journals for varying lengths of time, and/or retraction of the published work.

Author Name and/or Initials: Jacob Stauber

ASN American Society of Nephrology · JASN Journal of the American Society of Nephrology · KIDNEY360 Accessing Our World From Every Angle · CJASN Clinical Journal of the American Society of Nephrology

ASN Journal Disclosure Form

As per ASN journal policy, I have disclosed any financial relationships or commitments I have held in the past 36 months as included below. I have listed my Current Employer below to indicate there is a relationship requiring disclosure. If no relationship exists, my Current Employer is not listed.

There is no minimum financial threshold; individuals must disclose all financial relationships, regardless of the amount, with ineligible companies. Individuals must disclose for every category below, regardless of their view of the relevance of the relationship to the activity. (*"Ineligible companies" are those whose primary business is producing, marketing, selling, re-selling, or distributing health care products used by or on patients.*)

Date: 11/25/2025

Author Name: Mélanie Viltard

Manuscript ID: K360-2025-000777R1

Manuscript Title: A Switch in Iron Delivery Is Critical for Postnatal Kidney Development

Disclosure Statement: *(including all categories below*)*

Current Employer(s) & Affiliation(s): Fondation pour la Recherche en Physiologie, Brussels, Belgium
Grants / Contracts / Research Funding: none
Consultancy / Advisory Board: none
Honoraria / Speaking Fees: none
Equity / Stock Ownership: none
Patents / Royalties: none
Leadership / Board / Editorial Roles: none
Speakers' Bureau: none
Other Interests / Relationships: none

***Categories:** Employer; Ownership Interest; Consultancy; Research Funding; Honoraria; Patents or Royalties; Advisory or Leadership Role; Speakers Bureau; and Other Interests or Relationships

Author Acknowledgment: I understand that the information above will be published within the journal article, if accepted, and that failure to comply and/or to accurately and completely report the potential financial conflicts of interest could lead to the following: 1) Prior to publication, article rejection, or 2) Post-publication, sanctions ranging from, but not limited to, issuing a correction, reporting the inaccurate information to the authors' institution, banning authors from submitting work to ASN journals for varying lengths of time, and/or retraction of the published work.

Author Name and/or Initials: Mélanie Viltard

ASN
American Society of Nephrology
JASN
Journal of the American Society of Nephrology
KIDNEY360
Accessing Our World From Every Angle
CJASN
Clinical Journal of the American Society of Nephrology

ASN Journal Disclosure Form

As per ASN journal policy, I have disclosed any financial relationships or commitments I have held in the past 36 months as included below. I have listed my Current Employer below to indicate there is a relationship requiring disclosure. If no relationship exists, my Current Employer is not listed.

M. Werth reports the following:
Employer: Macroarray Diagnostics GmbH

I understand that the information above will be published within the journal article, if accepted, and that failure to comply and/or to accurately and completely report the potential financial conflicts of interest could lead to the following: 1) Prior to publication, article rejection, or 2) Post-publication, sanctions ranging from, but not limited to, issuing a correction, reporting the inaccurate information to the authors' institution, banning authors from submitting work to ASN journals for varying lengths of time, and/or retraction of the published work.

Name: Max Werth
Manuscript ID: K360-2025-000777R1
Manuscript Title: A Switch in Iron Delivery Is Critical for Postnatal Kidney Development
Date of Completion: November 25, 2025
Disclosure Updated Date: November 25, 2025

ASN
American Society of Nephrology
JASN
Journal of the American Society of Nephrology
KIDNEY360
Accessing Our World From Every Angle
CJASN
Clinical Journal of the American Society of Nephrology

ASN Journal Disclosure Form

As per ASN journal policy, I have disclosed any financial relationships or commitments I have held in the past 36 months as included below. I have listed my Current Employer below to indicate there is a relationship requiring disclosure. If no relationship exists, my Current Employer is not listed.

There is no minimum financial threshold; individuals must disclose all financial relationships, regardless of the amount, with ineligible companies. Individuals must disclose for every category below, regardless of their view of the relevance of the relationship to the activity. ("*Ineligible companies*" *are those whose primary business is producing, marketing, selling, re-selling, or distributing health care products used by or on patients.*)

Date: 11/25/2025

Author Name: Katherine Xu

Manuscript ID: K360-2025-000777R1

Manuscript Title: A Switch in Iron Delivery Is Critical for Postnatal Kidney Development

Disclosure Statement: *(including all categories below*)*

Current Employer(s) & Affiliation(s): Columbia University Irving Medical Center
Grants / Contracts / Research Funding: NIH/NIDDK K01
Consultancy / Advisory Board: none
Honoraria / Speaking Fees: none
Equity / Stock Ownership: none
Patents / Royalties: none
Leadership / Board / Editorial Roles: none
Speakers' Bureau: none
Other Interests / Relationships: none

***Categories:** Employer; Ownership Interest; Consultancy; Research Funding; Honoraria; Patents or Royalties; Advisory or Leadership Role; Speakers Bureau; and Other Interests or Relationships

Author Acknowledgment: I understand that the information above will be published within the journal article, if accepted, and that failure to comply and/or to accurately and completely report the potential financial conflicts of interest could lead to the following: 1) Prior to publication, article rejection, or 2) Post-publication, sanctions ranging from, but not limited to, issuing a correction, reporting the inaccurate information to the authors' institution, banning authors from submitting work to ASN journals for varying lengths of time, and/or retraction of the published work.

Author Name and/or Initials: Katherine Xu

ASN Journal Disclosure Form

As per ASN journal policy, I have disclosed any financial relationships or commitments I have held in the past 36 months as included below. I have listed my Current Employer below to indicate there is a relationship requiring disclosure. If no relationship exists, my Current Employer is not listed.

There is no minimum financial threshold; individuals must disclose all financial relationships, regardless of the amount, with ineligible companies. Individuals must disclose for every category below, regardless of their view of the relevance of the relationship to the activity. ("*Ineligible companies" are those whose primary business is producing, marketing, selling, re-selling, or distributing health care products used by or on patients.*)

Date: 11/25/2025

Author Name: Andrew Yaeh

Manuscript ID: K360-2025-000777R1

Manuscript Title: A Switch in Iron Delivery Is Critical for Postnatal Kidney Development

Disclosure Statement: *(including all categories below*)*

Current Employer(s) & Affiliation(s): Memorial Sloan Kettering Cancer Center
Grants / Contracts / Research Funding: none
Consultancy / Advisory Board: none
Honoraria / Speaking Fees: none
Equity / Stock Ownership: none
Patents / Royalties: none
Leadership / Board / Editorial Roles: none
Speakers' Bureau: none
Other Interests / Relationships: none

***Categories:** Employer; Ownership Interest; Consultancy; Research Funding; Honoraria; Patents or Royalties; Advisory or Leadership Role; Speakers Bureau; and Other Interests or Relationships

Author Acknowledgment: I understand that the information above will be published within the journal article, if accepted, and that failure to comply and/or to accurately and completely report the potential financial conflicts of interest could lead to the following: 1) Prior to publication, article rejection, or 2) Post-publication, sanctions ranging from, but not limited to, issuing a correction, reporting the inaccurate information to the authors' institution, banning authors from submitting work to ASN journals for varying lengths of time, and/or retraction of the published work.

Author Name and/or Initials: Andrew Yaeh

ASN American Society of Nephrology · JASN Journal of the American Society of Nephrology · KIDNEY360 Accessing Our World From Every Angle · CJASN Clinical Journal of the American Society of Nephrology

ASN Journal Disclosure Form

As per ASN journal policy, I have disclosed any financial relationships or commitments I have held in the past 36 months as included below. I have listed my Current Employer below to indicate there is a relationship requiring disclosure. If no relationship exists, my Current Employer is not listed.

There is no minimum financial threshold; individuals must disclose all financial relationships, regardless of the amount, with ineligible companies. Individuals must disclose for every category below, regardless of their view of the relevance of the relationship to the activity. ("*Ineligible companies*" *are those whose primary business is producing, marketing, selling, re-selling, or distributing health care products used by or on patients.*)

Date: 11/27/2025

Author Name: Yue Yu (Carly)

Manuscript ID: K360-2025-000777R1

Manuscript Title: A Switch in Iron Delivery Is Critical for Postnatal Kidney Development

Disclosure Statement: *(including all categories below*)*

Current Employer(s) & Affiliation(s): Skadden, Arps, Slate, Meagher & Flom
Grants / Contracts / Research Funding: none
Consultancy / Advisory Board: none
Honoraria / Speaking Fees: none
Equity / Stock Ownership: none
Patents / Royalties: none
Leadership / Board / Editorial Roles: none
Speakers' Bureau: none
Other Interests / Relationships: none
Spouse / Partner Interests: none

***Categories:** Employer; Ownership Interest; Consultancy; Research Funding; Honoraria; Patents or Royalties; Advisory or Leadership Role; Speakers Bureau; and Other Interests or Relationships

Author Acknowledgment: I understand that the information above will be published within the journal article, if accepted, and that failure to comply and/or to accurately and completely report the potential financial conflicts of interest could lead to the following: 1) Prior to publication, article rejection, or 2) Post-publication, sanctions ranging from, but not limited to, issuing a correction, reporting the inaccurate information to the authors' institution, banning authors from submitting work to ASN journals for varying lengths of time, and/or retraction of the published work.

Author Name and/or Initials: Yue Yu (Carly)
