# Supplementary material for: A Switch in Iron Delivery Is Critical for Postnatal Kidney Development
Source: Kidney360. 2026 Feb 17;7(5):982–1002. doi: 10.34067/KID.0000001064 (PMC13229437; doi:10.34067/KID.0000001064)
Supplement: Supplementary file 2 [file kidney360-7-0982-s002.pdf]

Table of Contents for Supplemental Files

1. **Supplemental Figures**
2. **Supplemental Figure Legends**
3. **Methods**
4. **Materials**
5. **Supplemental References**

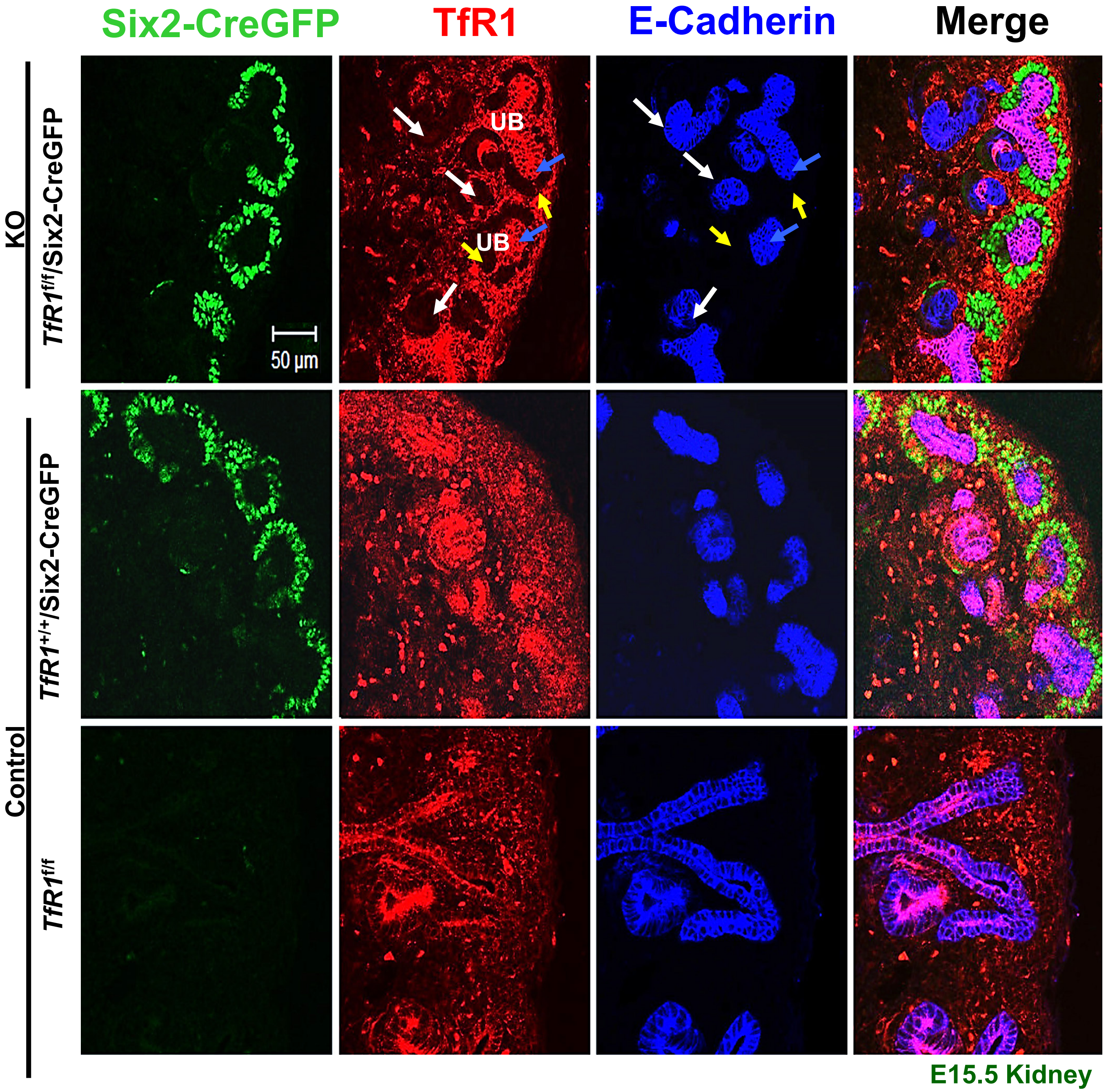

Supplemental Figure 1

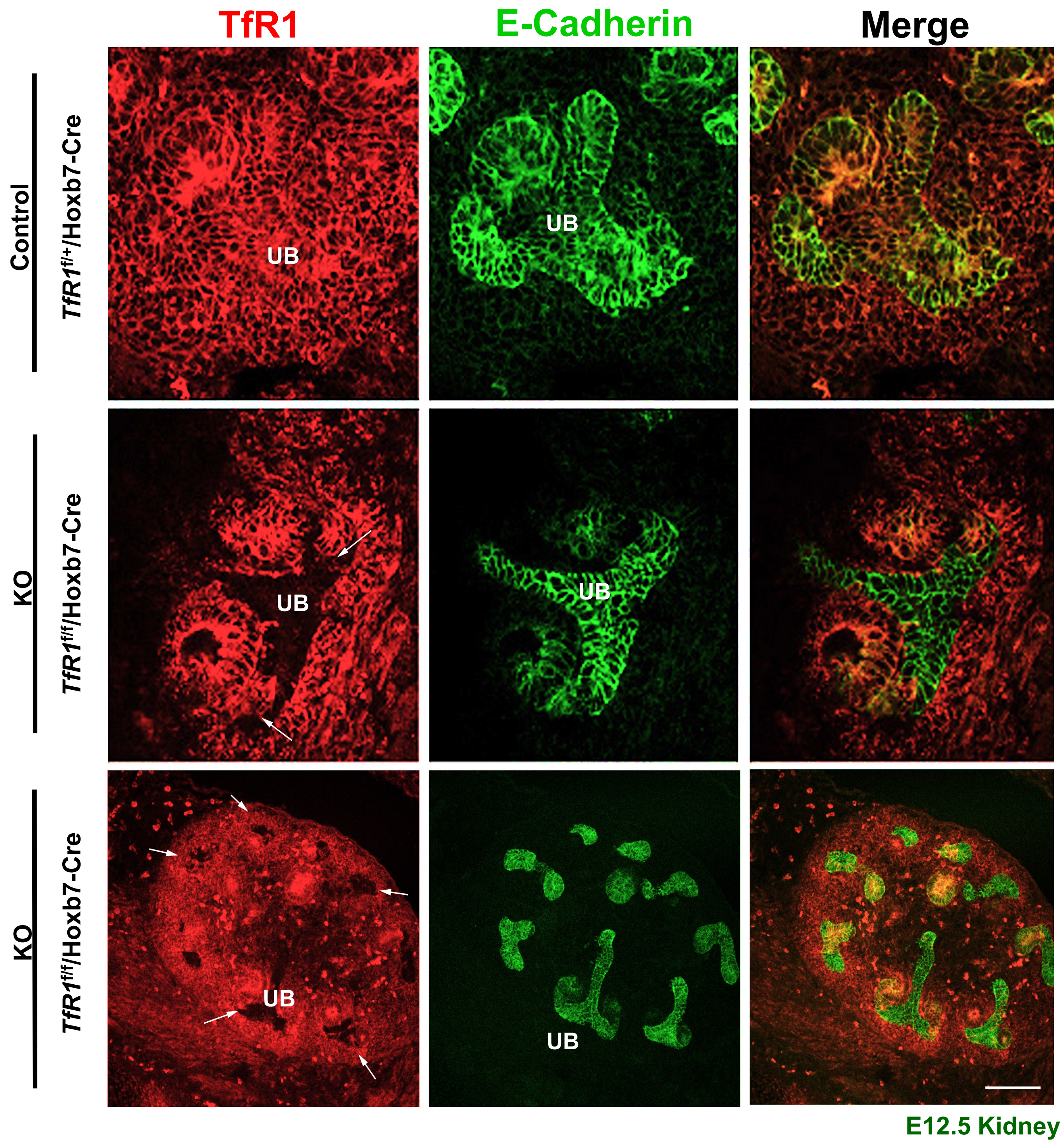

Supplemental Figure 2

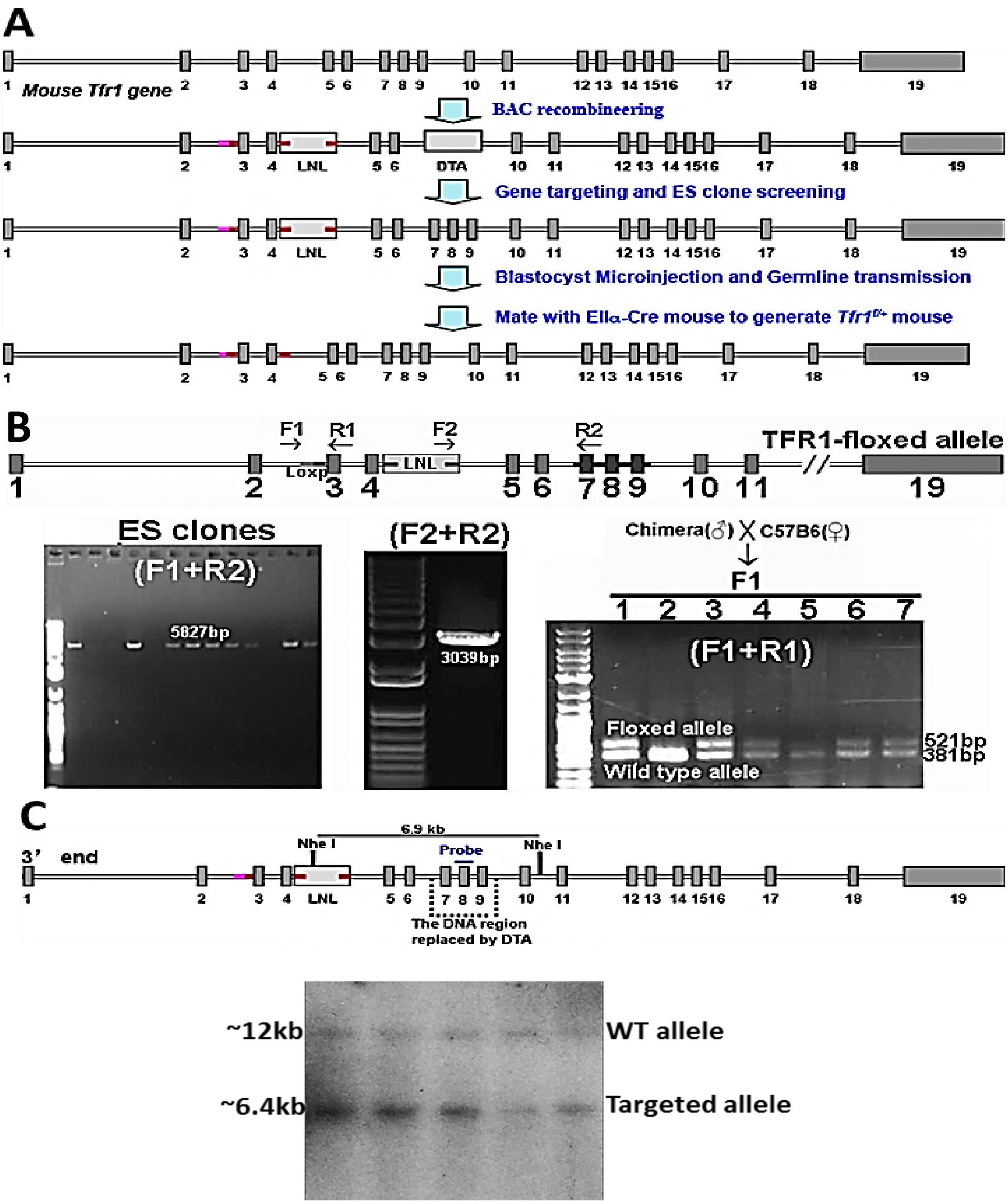

Supplemental Figure 3

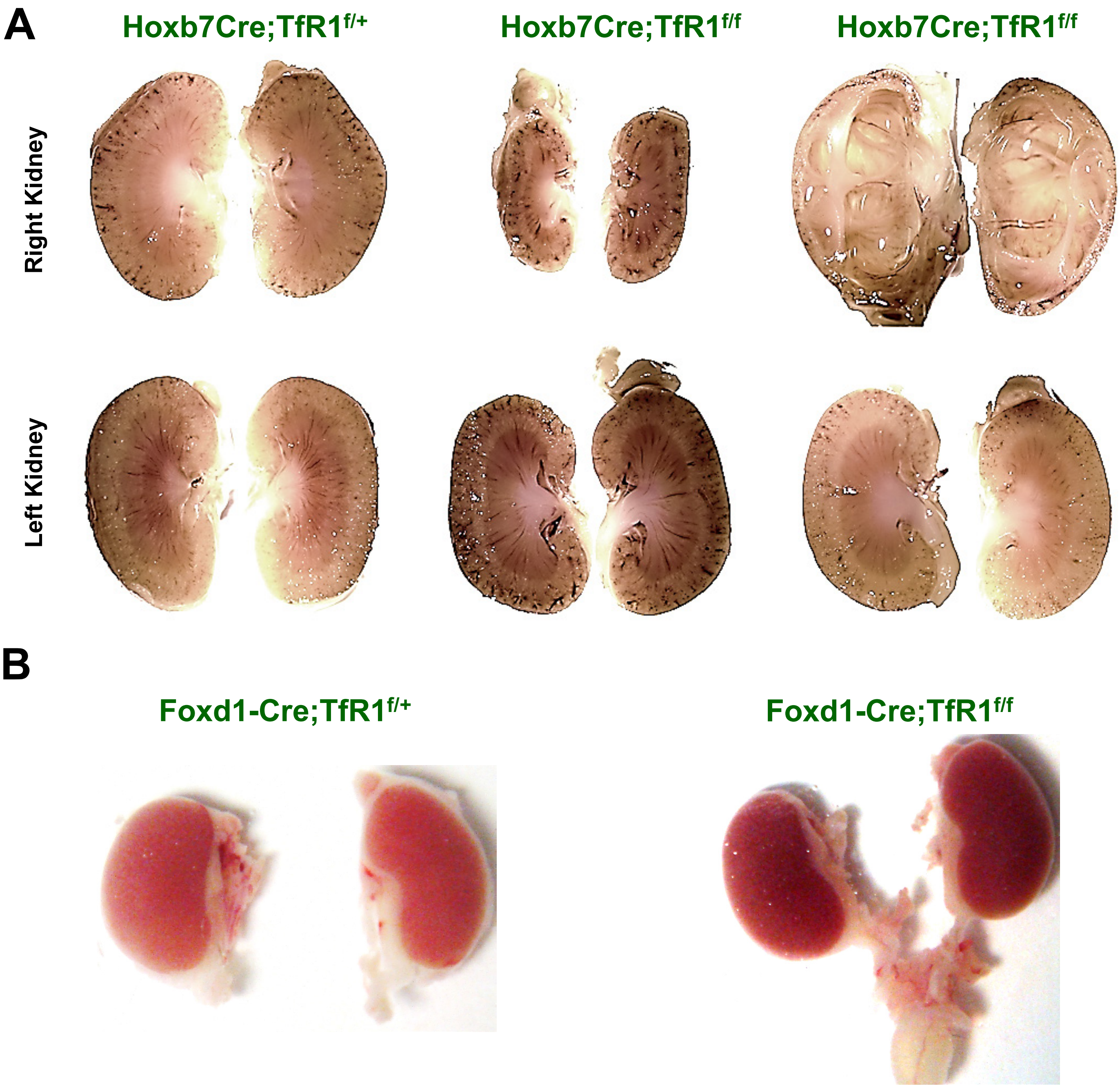

Supplemental Figure 4

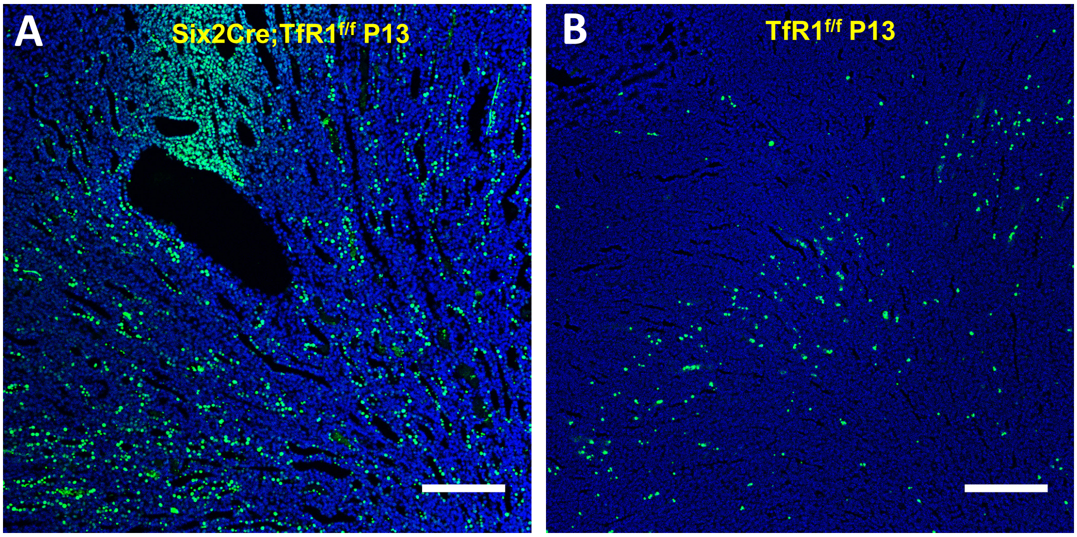

Tunel Tunel

Supplemental Figure 5

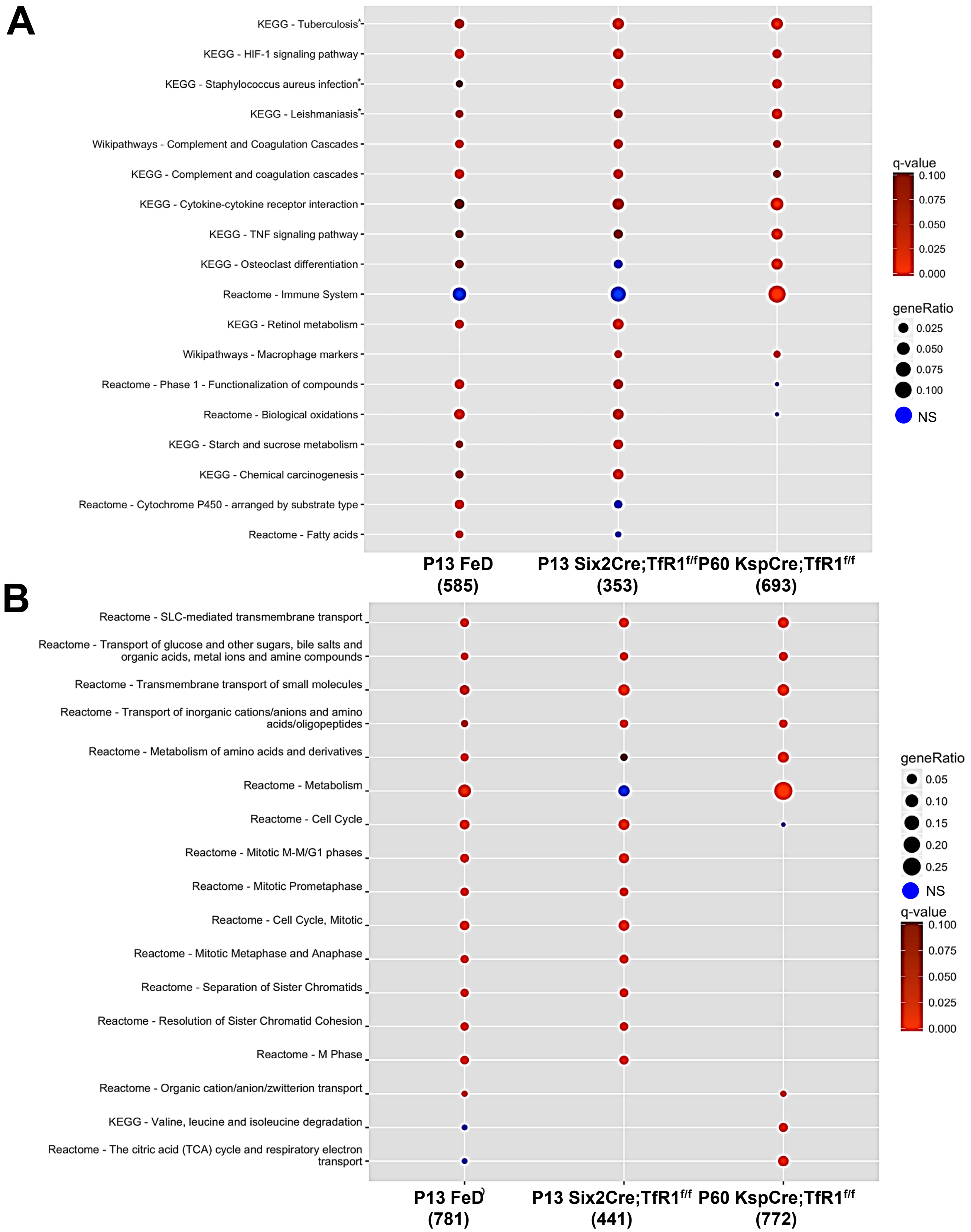

Supplemental Figure 6

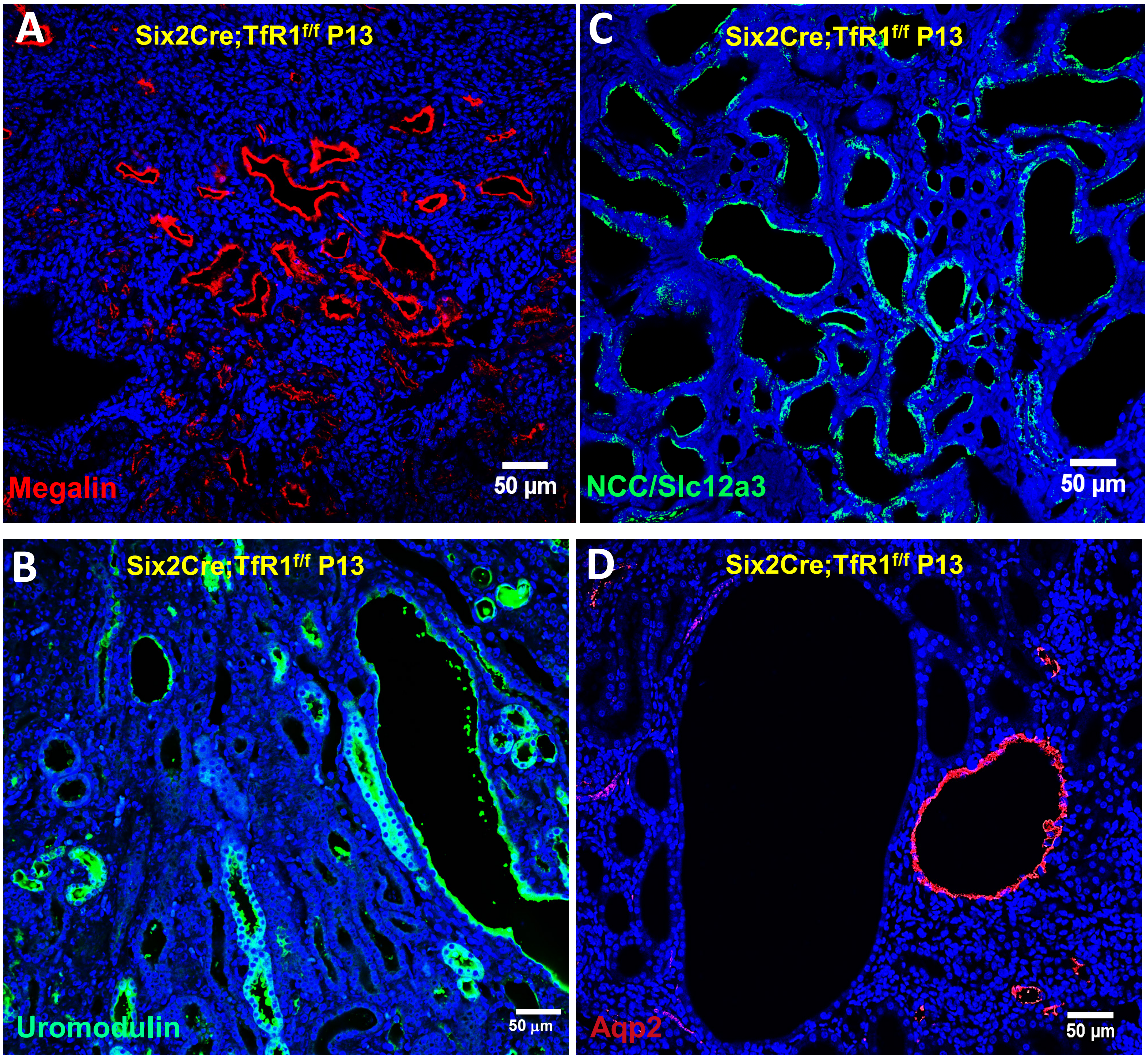

Supplemental Figure 7

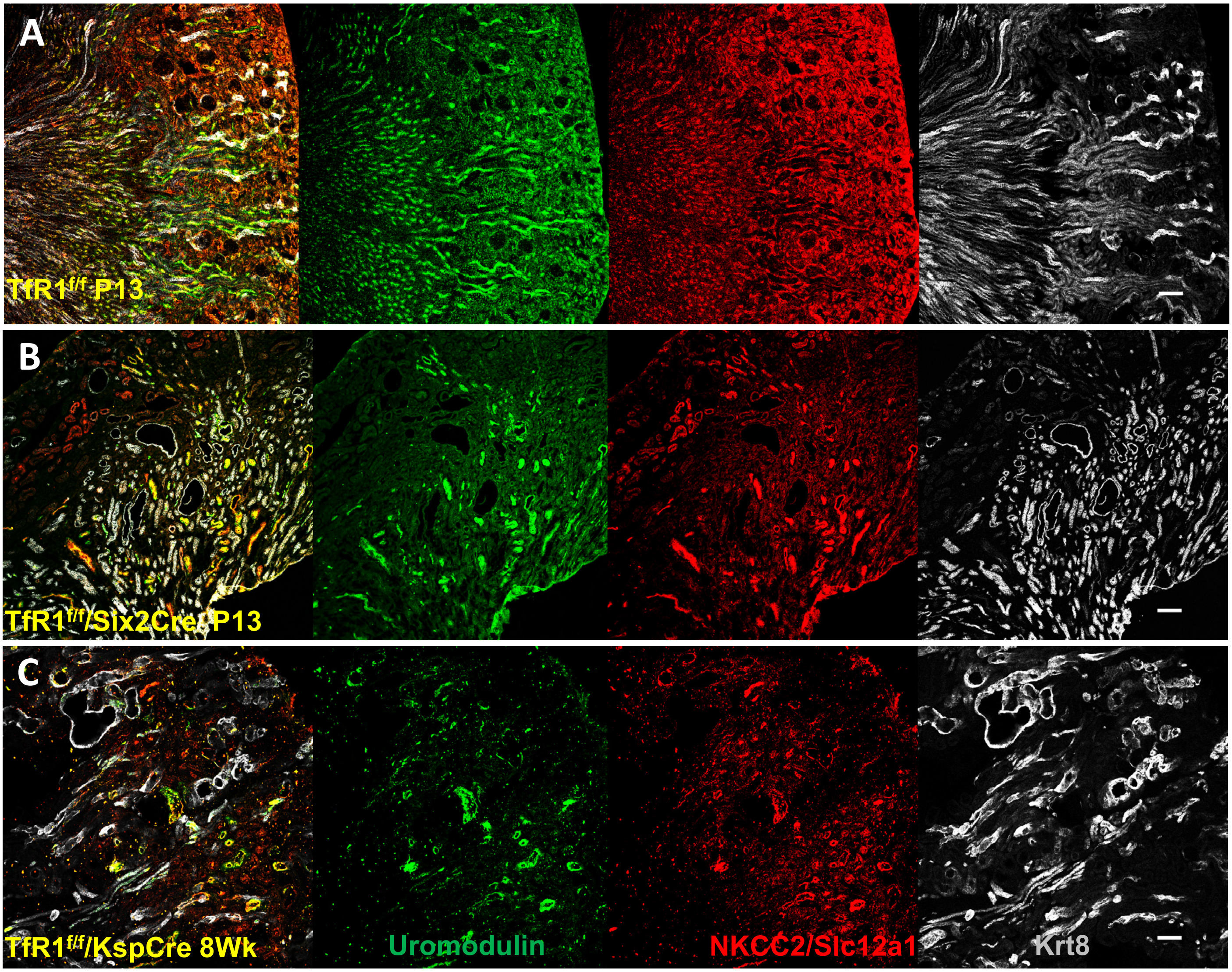

Supplemental Figure 8

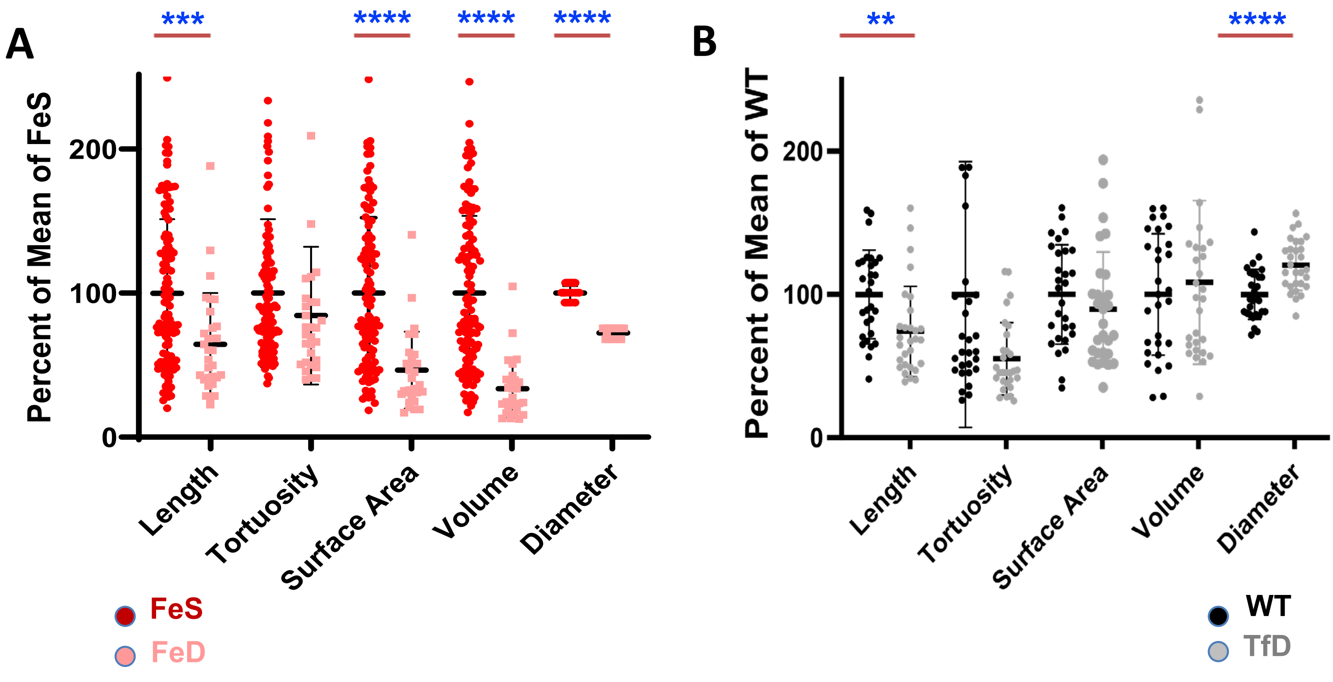

Supplemental Figure 9

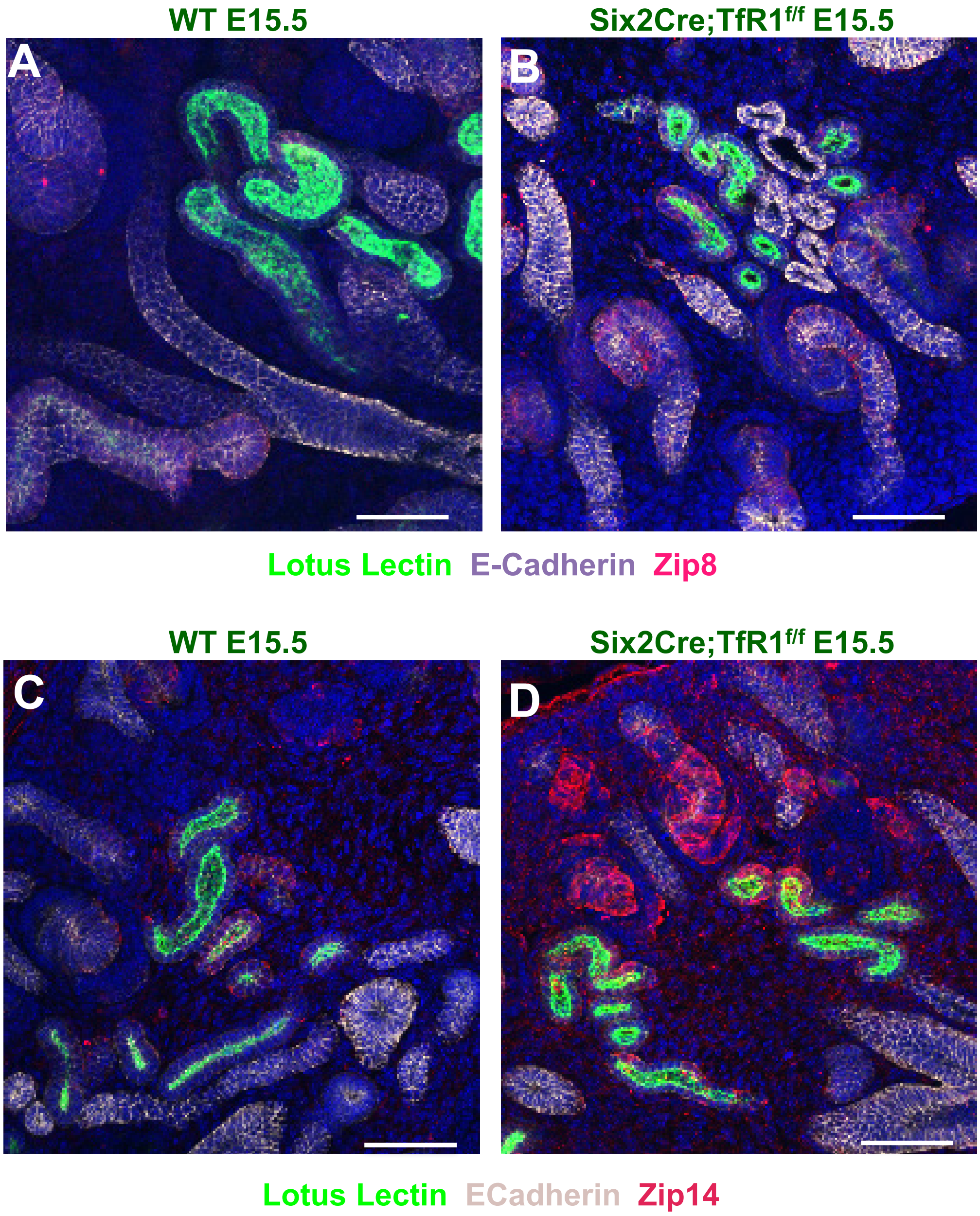

Supplemental Figure 10

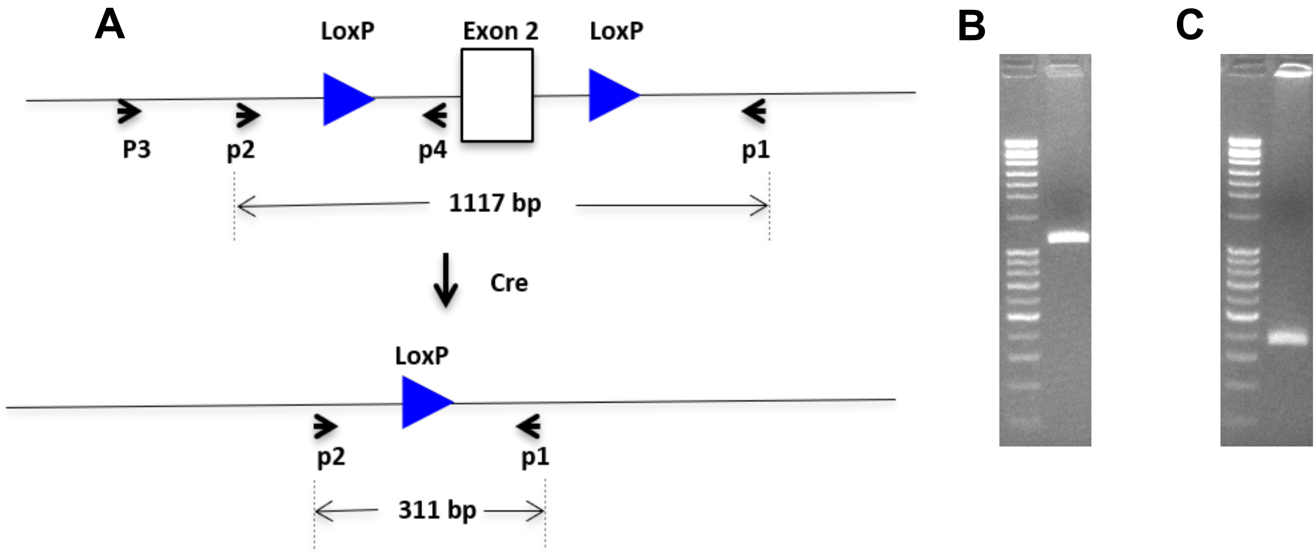

P13 Six2Cre;TfR1$^{f/f}$;Hif1α$^{f/f}$

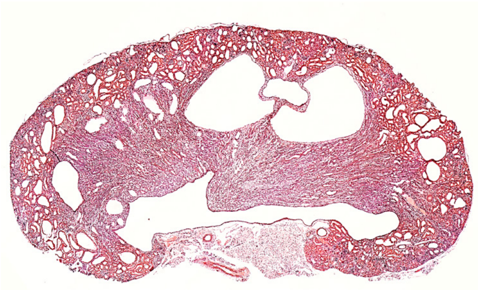

Supplemental Figure 11

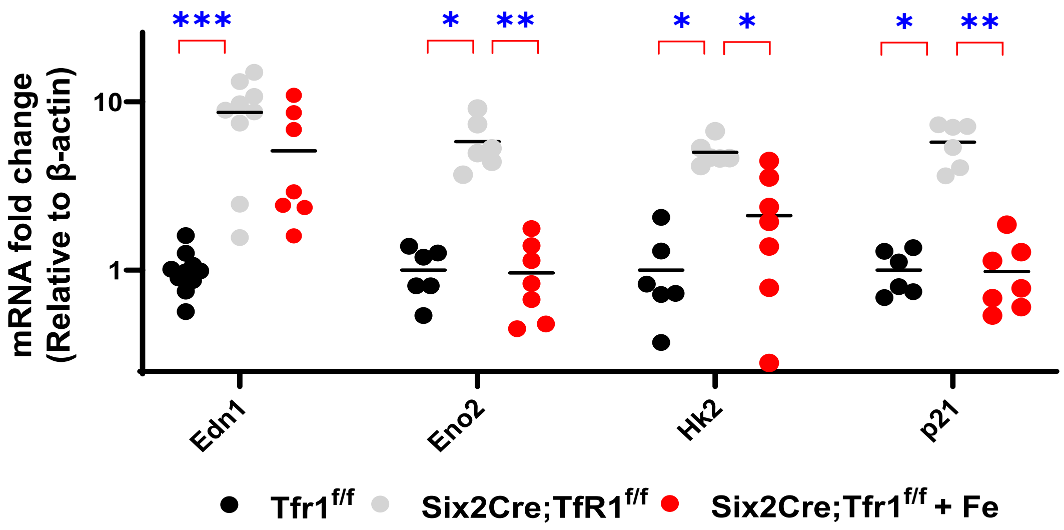

Supplement Figure 12

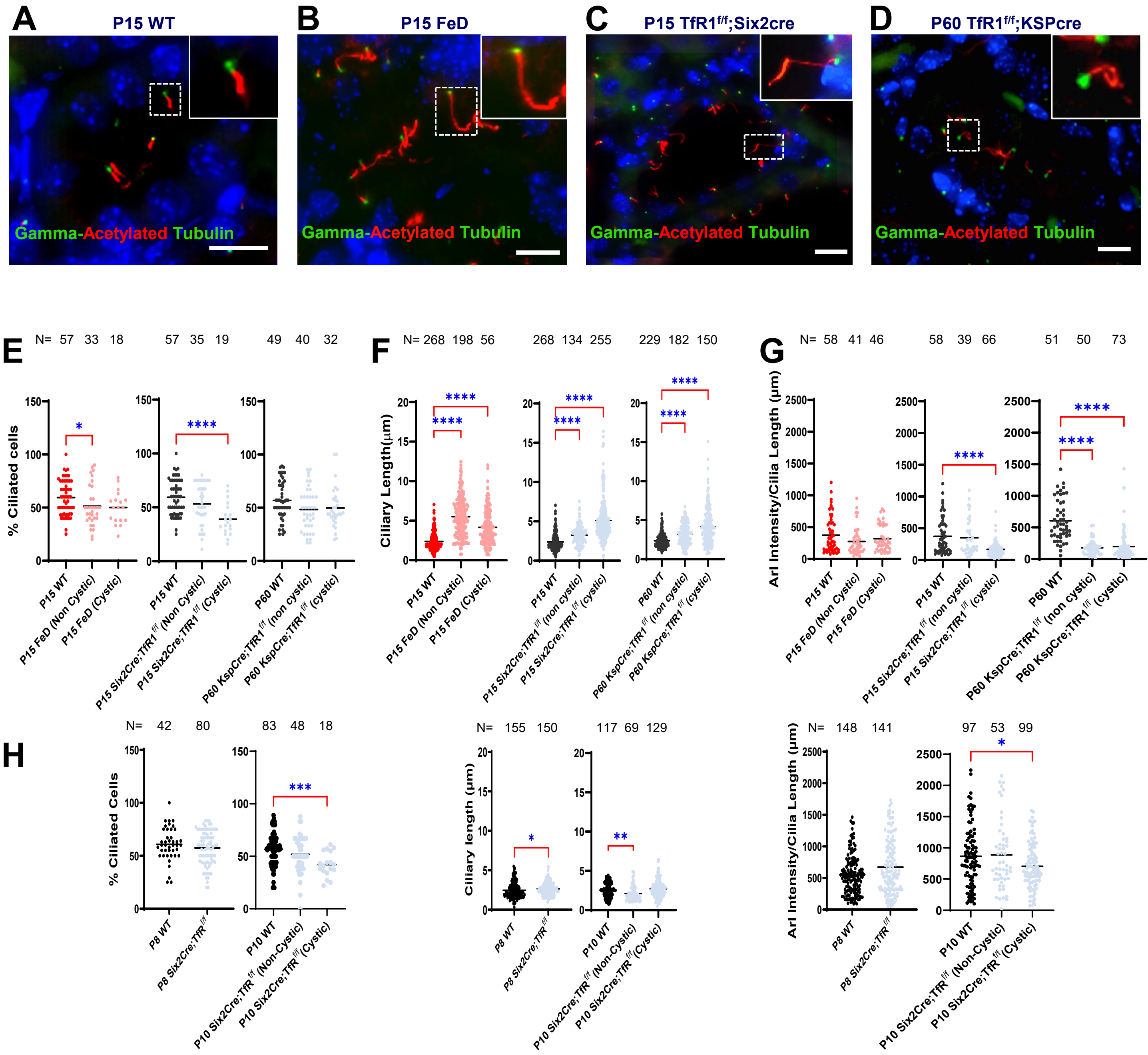

Supplement Figure 13

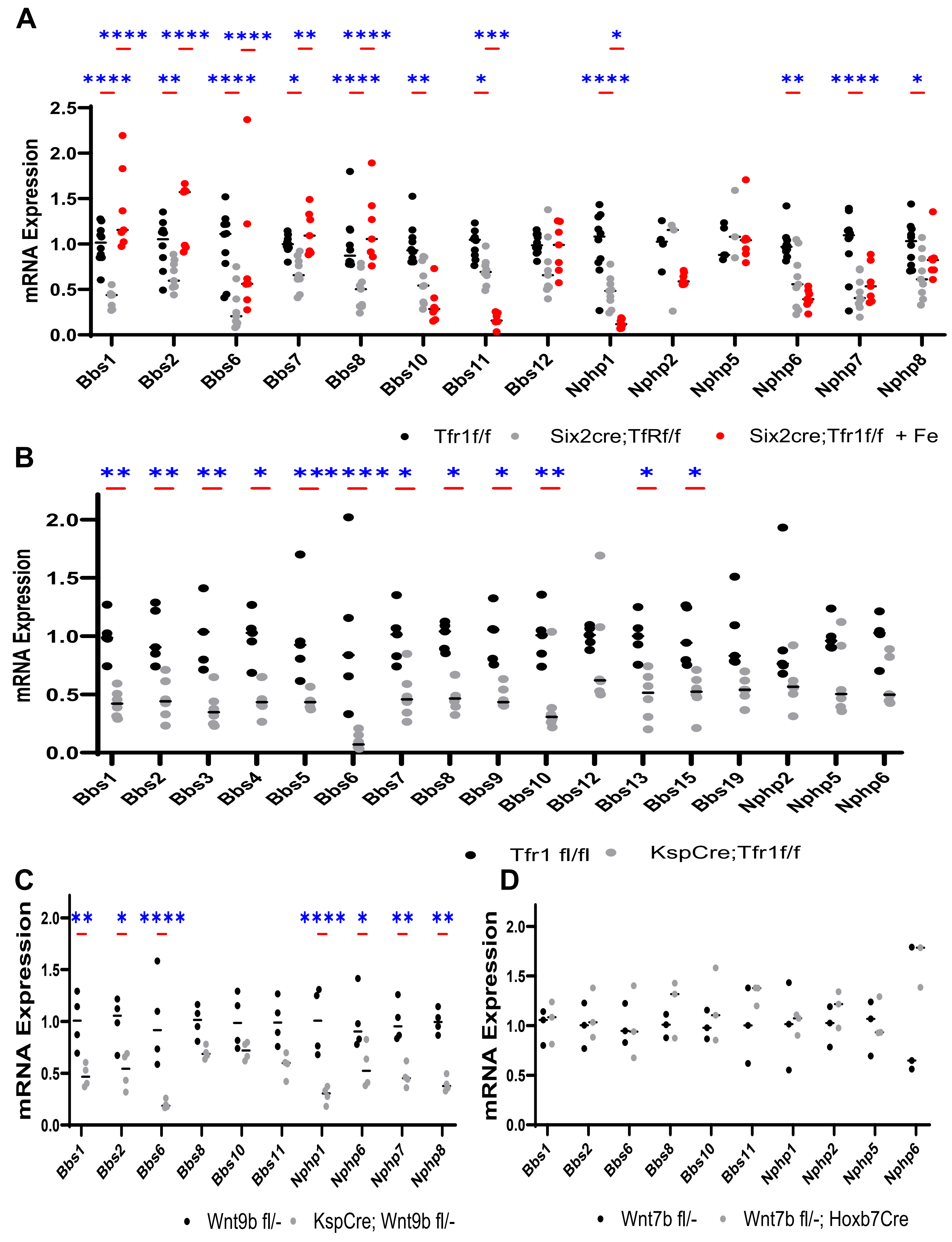

Supplement Figure 14

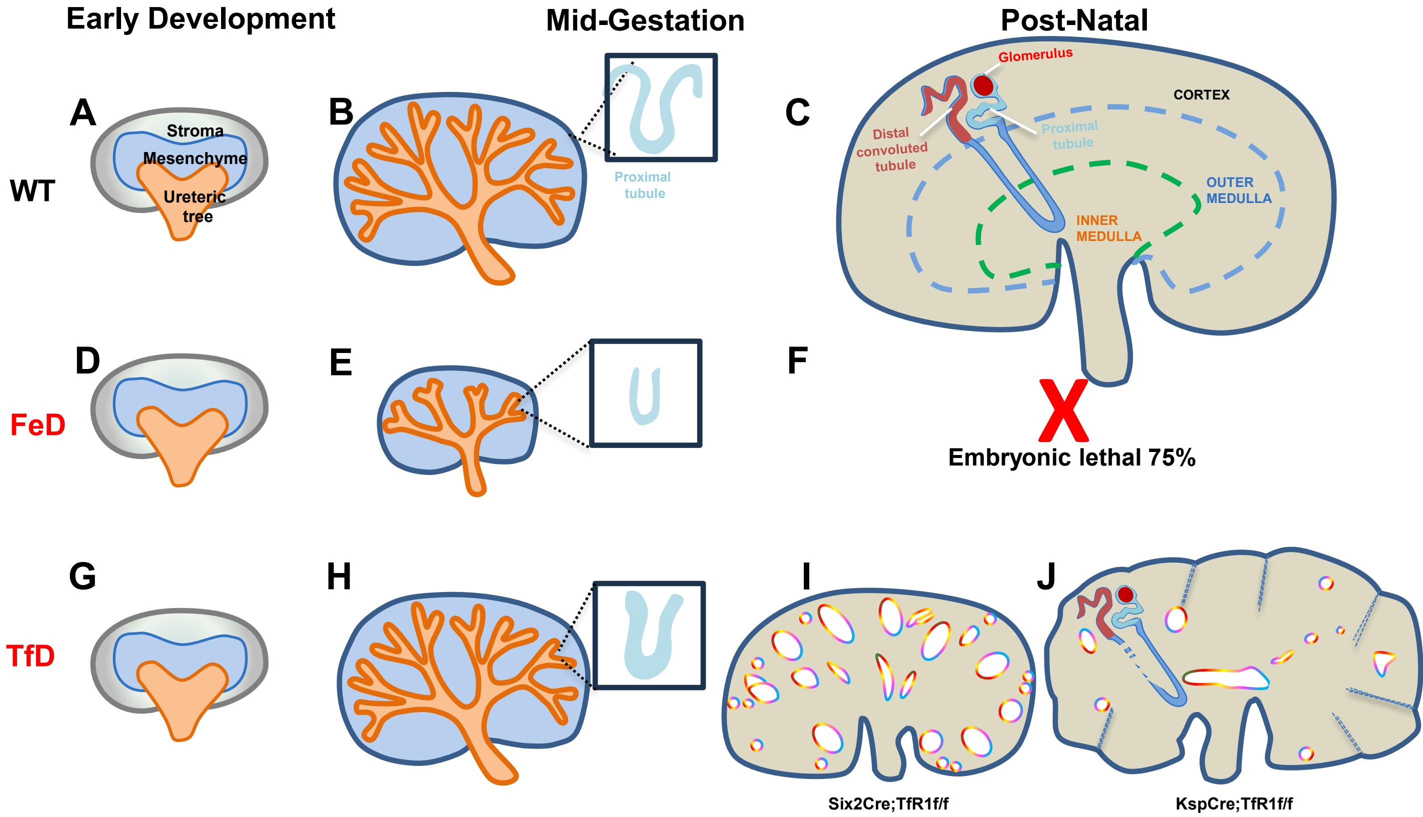

Supplement Figure 15

Supplemental Figure Legends

Supplemental Figure 1 Deletion of *TfR1* with the *Six2Cre*EGFP driver (E15.5) did not block epithelial induction. *TfR1* (red) was expressed throughout the cortex of the kidney. Deletion of *TfR1*: note ribbon of SIX2-GFP$^+$, *TfR1*-null mesenchymal progenitors (yellow arrows), surrounding UB tips (blue arrows). Despite *TfR1* deletion mesenchymal progenitors produced E-Cadherin$^+$ C- and S- shaped epithelial nephrons (white arrows). In contrast to the knockout, wild type littermates demonstrated homogenous *TfR1* staining. Bar= 50μm.

Supplemental Figure 2 Deletion of *TfR1* with the *Hoxb7Cre* driver (E12.5) did not block growth of UB. WT TfR1 (red) was expressed throughout the ureteric bud but deletion of *TfR1* did not abolish morphogenesis of E-Cadherin$^+$ ureteric ducts (white arrows). Bar=100μm.

Supplemental Figure 3 Generation of *TfR1* floxed mice. (**A**) Exons 3 and 4 of *TfR1* were flanked with LoxP sites using BAC recombineering. (**B**) Identification of *TfR1*-floxed ES clones by PCR screening. (**C**) Confirmation of homologous targeting by Southern Blot. DNA was restricted with Nhe I. (**D**) Germline-transmitted F1 female mice were mated with male *EIIa-Cre* mice to remove the neo^r cassette and to generate *TfR1*$^{f/+}$.

Supplemental Figure 4 *TfR1* deletion in Ureteric Bud and Stroma (**A**) Deletion of *TfR1* in the ureteric bud (*Hoxb7Cre*;*TfR1*$^{f/f}$) resulted in grossly normal kidneys at P8. One kidney demonstrated hypoplasia, and one demonstrated hydronephrosis, but most kidneys were grossly unaffected; n=20 kidneys. (**B**) Deletion of *TfR1* in stroma (*Foxd1Cre*;*TfR1*$^{f/f}$) displayed grossly normal kidney development; n=8 kidneys.

Supplemental Figure 5 Apoptosis in the corticomedullary junction and medulla at P13. (**A**) *Six2CreEGFP*;TfR$^{f/f}$ mice (**B**) *TfR*$^{f/f}$ mice. Tunel Stain$^+$. Bars=200μm.

Supplemental Figure 6. Gene regulation in TfD and FeD models. (**A**) TfD and iron deficient FeD kidneys upregulate HIF1 as well as inflammatory and cellular immune responses. Upregulated pathways are listed in order of average q-value. Q-values are FDR corrected. Gene ratio represents the fraction of regulated genes ascribed to a given pathway. (**B**) Downregulated pathways were notable for membrane transporters and cell cycle components. Enrichments of gene expression was identified using DESEQ2 by comparison of our models of iron deficiency with littermate wild type controls. Regulated genes were inputted for enrichment analysis. n= 3 mice per model.

Supplemental Figure 7. Deletion of *TfR1* in the nephron resulted in cystic hypodysplasia after birth. (**A**). Proximal Tubule- Megalin; (**B**). TALH- Uromodulin (**C**). Distal convoluted tubule- NCC/Slc12a3. (**D**). Connecting Tubule- Collecting Duct- AQP2. Bars=50μm

Supplemental Figure 8. Defective growth of the TALH. (**A**) Wild type. (**B**) *Six2CreEGFP*;*TfR1*$^{f/f}$ and (**C**) *KspCre;TfR1*$^{f/f}$ abolish the Limb of Henle (Uromodulin$^+$ and NKCC2$^+$/Slc12a1). Bars=100μm.

Supplement Figure 9. Proximal Tubule growth defects, comparison of FeD and TfD: (**A**) FeD severely restricted the growth of Proximal Tubules (length, diameter, volume, surface area, and tortuosity). 118 wild type and 29 TfD Proximal Tubules were measured in 5 mice each. Data from Supplemental Figure 10Q. (**B**) TfD demonstrated less severe effect. Linear growth of Proximal Tubules was suppressed, whereas volume, surface area and tortuosity were unchanged and circumferential growth enhanced in 29 TfD (7 mice) compared with 31 wild type (8 mice) tubules. Data from Figure 1H. **A, B**. Two tailed Mann-Whitney Test;

Bonferroni corrected for multiple testing. Data are displayed as the percentage of means of wild type kidneys ± Std. $**p < 0.01$; $****p < 0.0001$.

Supplement Figure 10 **NTBI Transporters are upregulated by in TfD.** (**A**, **B**). ZIP8 expression is increased in UB and Proximal Tubule; (**C**, **D**). ZIP14 expression is increased in S-shaped bodies and Proximal Tubules. A-D Bars=100μm.

Supplement Figure 11 **Conditional deletion of *Hif1* exon 2 by *Six2Cre;Hif1$^{f/f}$*** (**A**) The location of the loxp site in the intron 1 was confirmed by Sanger sequencing of DNA from *HIF1$^{f/f}$* mice, and primer pairs flanking floxed-exon2 were identified. (**B**) Primer pair p1-p2 amplified a 1117bp fragment of DNA from *HIF1$^{f/f}$* kidney cortex (**C**) and a 311bp fragment from *Six2Cre;HIF1$^{f/f}$* cortex. (**D**). Deletion of both *HIF1α* and *TfR1* did not mitigate TfD cystogenesis at P13, n=3 mice.

Supplement Figure 12 **HIF1α targeted genes were enriched in Six2Cre;Tfr1$^{f/f}$ mice** (*Edn1*, padj<0.001; *Eno2*, padj=0.017; *HK2*, padj=0.017; *p21/Cdkn1a*, padj=0.017); n=6-9 vs n=10-6 WT mice. Treatment with iron suppressed some of these genes (e.g. *Eno2*, padj=0.009; *HK2*, padj=0.019; *p21/Cdkn1a*, padj=0.009); n=7. Untreated, age matched TfR1$^{f/f}$ littermates (WT) served as controls, normalized to 1. Kidneys were harvested at P13. *p<0.05; **p<0.01; ***p<0.001. Mean ± Std. Mann-Whitney with Bonferroni Correction for multiple testing.

Supplement Figure 13 **Response of the Ciliary System to Iron Deficiency.** (**A-D**) Ciliation was measured with anti-γ-Tubulin and anti-Acetylated α-Tubulin. (**E-G**) Ciliary length was increased in FeD and TfD kidneys. (**E**) Ciliated cells are slightly reduced; padj<0.001 (**F**) but cilia were prominently longer in FeD: Cystic padj<0.001; Non-Cystic tubules, padj<0.001; P15 TfD: Cystic p<0.001; Non-Cystic p<0.001 and P60 TfD: Cystic padj<0.001, Non Cystic tubules padj<0.001. (**G**) ARL13b labeling density was decreased in P15 TfD: Cystic padj<0.001 and P60 TfD: Cystic p<0.001, Non-Cystic: p<0.001. (**H**). Note P8-P10 depict limited disruption. (**E-H**) Kruskal Wallis corrected by Dunn's test; two-way comparisons use Mann-Whitney. n= 6-10 mice were used for microscopic analysis. Mean±SD. $*p < 0.05$, $**p < 0.01$.

Supplement Figure 14 (**A**) **Downregulation of *BB*somes Genes**: *BB*1,2,6,7, 8 (eg. *BB1* FeD p=0.002 ~50%) (qRT-PCR) by *TfR1;Six2Cre*. Inoculated or iron at P8 (red), normalized *BB*some 1,2,6,7,8 but was ineffective for other *BB*some and *Nphp* genes. 2-way Anova, corrected with Bonferroni n= 6-10 mice. (**B**) Similar data were obtained by deletion of *TfR1;KspCre*. 1-way Anova, corrected with Bonferroni n= 4-6 mice. (**C**) Downregulation of a similar cohort of *BB*somes and *Nphp* Genes by deletion of *Wnt9b* using *KspCre*. 1-way Anova, corrected with Bonferroni n= 4 mice. (**D**) Failure to regulate *BBsomes* and *Nphp* genes by *Wnt7b* deletion. 1-way Anova, n= 3 mice. $*p < 0.05$, $**p < 0.01$, $***p < 0.001$, $****p < 0.0001$

Supplement Figure 15 **Summary Diagram.** Differential responses to iron deprivation according to model of deficiency (FeD *vs* TfD) and stage of development.

Supplemental Methods

Animals

Animal use was approved by the Columbia Institutional Animal Care and Use Committee (IACUC). C57BL/6 mice (8 weeks, Charles River, USA) and engineered strains were maintained in an AAALAC-accredited facility accordance with the Guide for the Care and Use of Laboratory Animals. Mice were free of viruses (e.g. hepatitis, parvovirus, rotavirus, norovirus, encephalomyelitis, adenovirus, polyoma), bacteria (e.g. Mycoplasma, Campylobacter, Salmonella, Staphylococcus, Streptococcus) and ecto- and endoparasites (e.g. Pinworm, giardia, cryptosporidium). Mice were housed at a maximum of 5 mice per cage on autoclaved bedding (ALPHA-dri/Cob Blend, Shepherd Specialty Papers, WF Fisher and Son, Somerville, NJ) in 12 hour-light dark cycle[1]. Male and female mice were used in equal proportion.

Mice had access ad libitum to an irradiated synthetic iron-deficient diet (2-6ppm iron, TD.80396, Harlan, USA, "FeD" group) or an iron sufficient diet (220ppm iron, PicoLab 5053, "FeS" group) for 3 weeks prior to mating and throughout pregnancy. We chose this schedule because: (1) inadequate Fe consumption is responsible for the majority of the Fe deficient pregnancies[2,3] and conversely iron supplementation before conception prevents iron deficiency; (2) iron deficient diets are effective when initiated two-three weeks prior to conception[1,4]; (3) the kidney demonstrates its full range of developmental programs by mid gestation, E15.5-E16. Plug date was designated as E0.5. Kidney size was analyzed using ImageJ Software. Statistical analysis was performed with the Student's t-test. See Supplemental Table 1 for mouse sources.

Deletion of *TfR1*

Exons 3 and 4, encoding the sole transmembrane domain of *TfR1* were floxed using a standard BAC recombineering techniques. Briefly, C57BL/6 mouse BAC DNA clone (RP23159G11) in DH10 E. *coli* was obtained from BACPAC Resources (http://bacpac.chori.org, Oakland, CA), and transferred to SW105 competent E. *coli*. A Frt-Neo^r-Frt-Loxp (FNFL) cassette was introduced 205bp upstream of exon 3 followed by Frt-based recombination resulting in a single Loxp sequence. Next, a Loxp-Neo^r-Loxp cassette and a Diphtheria Toxin A (DTA) gene were introduced 206bp and 2267bp downstream of exon 4, respectively. The Loxp sites were functionally verified in SW106 E. *coli* cells by activating Cre recombinase with 0.1% arabinose followed by PCR to confirm deletion of the DNA fragment between the flanking Loxp sites. *TfR1*-Loxp1-LNL-DTA BAC DNA was verified by DNA sequencing.

TfR1-targeted BAC DNA was electroporated into KV1 ES cells (HICCC Transgenic Shared Resource, Columbia). PCR at the 5' and 3' arms flanking the knockin site documented that 8% of embryonic stem cell clones were correctly targeted by homologous recombination. Southern Blotting verified the site-specific recombination and integration of the first Loxp site and the LNL cassette into the *TfR1* gene in ES cells (Supplemental Figure 3). The Neo^r cassette was removed by mating the germline-transmitted heterozygous

TfR1$^{f/+}$ female mice with male EIIa-Cre mice (Jackson Laboratory); Neo^{r} deletion was identified by PCR-screening. Both *TfR1*$^{f/+}$ and *TfR1*$^{f/f}$ mice developed normally.

Tissue specific deletions of *TfR1* utilized *Foxd1-Cre*[5], *Six2-CreEGFP*[6], and *Hoxb7-Cre*[7]. We authenticated our Cre colonies by mating with Rosa-Tomato-GFP mice and demonstrating segment specific fluorescence[8]. As an alternative approach to study the role of *TfR1*, we previously generated *Rosa26-Gfp-TfR1*$^{-/-}$ ES, introduced six of these cells into wildtype blastocysts, and analyzed their distribution in chimeric embryos[9].

To determine the role of HIF1α in the development of *TfR1* mediated cysts, *Six2CreEGFP*;*TfR1*$^{f/f}$ mice were crossed with *HIF*$^{f/f}$ mice (www.jax.org/strain/007561). Deletion of HIF1α was confirmed by Sanger sequencing followed by the development of flanking PCR primers.

To rescue iron deficiency, *Six2CreEGFP*;*TfR1*$^{f/f}$ mice were inoculated (*i.p.*) iron sucrose (1mg, Venofer) at P8. FG-4592 (2mg/kg; Roxadustat; Cayman Chemical in 1% DMSO) was introduced (*i.p.*) for three days P8-P10.

Blood and Plasma Measurements

Whole blood (50μl) was collected in heparinized vials from tail vein, and hematocrit measured by I-STAT EC-8+ cartridges (Abbott Point-of Care, USA). Fetal plasma NTBI and transferrin saturation were measured in independent pooled samples. NTBI was measured by mixing plasma (30μL) with nitrilotriacetic acid (150μL; 80mM, pH7.0; Sigma-Aldrich) for 30 min at room temperature[10,11] and measuring the ultrafiltrate (Amicon, 10-kDa cutoff, Millipore; 10,620 x g, 15°C for 45 min) by Graphite Furnace Atomic Absorption Spectrophotometer (Analyst 800; PerkinElmer) at the Trace Metals Core Facility of Columbia University (Mailman School of Public Health). The iron-nitrilotriacetic acid (150μL; 100mM, pH7.0; Sigma-Aldrich) was quantified and subtracted as a blank.

Determination of Tissue Iron

Total organ iron was determined using a wet washing procedure[12]. In brief, kidneys were dissected, desiccated and solubilized in a solution with 3 M Hydrochloric and 10% Trichloracetic acid. Absorbance was measured at 535 nm after addition of chromogen (1.6 mM bathophenanthroline, 2 M sodium acetate, and 11.5 mM thioglycolic acid) to determine non-heme iron.

Quantitative RT-PCR

Total RNA was extracted (Tri-Reagent, Sigma) from adult and embryonic organs, treated with DNase I (Promega) and reverse-transcribed using the High-Capacity cDNA Reverse Transcription Kit (Applied Biosystems, USA). Expression of target genes was quantified by a 7500 fast real-time PCR system (Applied

Biosystems, USA) and normalized to ß-actin message. Gene-specific Q-PCR amplification was verified by both melting curve analysis and agarose gel electrophoresis. Primers are listed in Supplemental Table 1.

RNA-Seq

Total RNA was isolated using Ambion TRIzol Reagent (Life Technologies, Carlsbad, CA). Integrity was assessed on RNA 6000 Chips using an Agilent 2100 Bioanalyzer (Agilent Technologies, Santa Clara, CA). Poly-A pull-down was used to enrich mRNAs (~400ng per sample, sample RIN was >6.0) and libraries were prepared using single-end 100bp reads with Illumina TruSeq RNA prep kits (Illumina, San Diego, CA). Libraries were sequenced using Illumina HiSeq2000 at Columbia Genome Center.

The quality of our samples was assessed by mapping quality, which reported: a median of 95.8% mapped reads per sample (IQR: 95.3-96.3%) of which 89.8% were uniquely mappable across all samples (IQR: 88.8-90.3%). Subsequently, all downstream analyses were performed exclusively on uniquely mapped reads, ensuring that multimapping reads did not influence the results. Second, we assessed 3′ bias using Picard CollectRnaSeqMetrics. Across all samples, the median 5′–3′ bias ratio was 0.50 (IQR 0.43–0.55), indicating balanced transcript coverage without evidence of strong RNA degradation or systematic 3′ bias. Moreover, downstream analyses were performed at the gene level, where even modest 3′ coverage bias would not materially affect read assignment or differential expression results, as counts are aggregated across all annotated exons.

Illumina RTA was used for base calling and CASAVA (version 1.8.2) was used for converting base call files (.BCL) to FASTQ format and for sequence adaptor trimming. After demultiplexing, fastq files were checked for quality using FASTQC (http://www.bioinformatics.babraham.ac.uk/projects/fastqc). Reads were then trimmed using TRIMMOMATIC[13] with parameters LEADING:10, TRAILING:10, MINLEN:30. Alignment was done against mouse mm9 genome using TOPHAT v2.0.11.[14]. Raw reads per gene were counted using HTSEQ[15] and differential expression was done using DESEQ2[16]. For pathway enrichment, we used the CONSENSUSPATHDB online tool[17]. Pathway enrichment analysis was limited to genes with Log2 Fold Change > 1 and padj<0.05; up- and down-regulated genes were treated separately. Data were deposited in GEO (https://www.ncbi.nlm.nih.gov/geo/query/acc.cgi?acc=GSE100254 (reviewer token mtefiwqyjverlet).

Immunofluorescence, Immunoblots and 3-D Analysis

E15.5 embryos and postnatal kidneys were fixed for 1 hour in 4% paraformaldehyde (PFA), embedded in OCT (Fisher Scientific), cryo-sectioned (15μm) and probed with a variety of antibodies (Supplemental Table 1). HIF staining was performed according to[18,19].

For proliferation assays, EDU (5-ethynyl-2'-deoxyuridine; 50 mg/g) was introduced 4 hours prior to sacrifice and detected using Click-iT™ EdU Alexa Fluor™ according to the Thermo Fischer Scientific protocol. For cell death, Terminal Deoxynucleotidyl Transferase-mediated dUTP nick end labeling (TUNEL) was performed according to Roche Diagnostics.

Glomeruli, Proximal Tubules, and EdU labelling were quantified with 20X objectives. For tubule areas, fluorescent images were binarized using the default method in ImageJ Auto Threshold and the tubule area was compared to the total area of cortex or medulla. To quantify Proximal Tubule diameters, the outer diameter of megalin$^+$ immunofluorescence was measured. The cystic index was defined in H&E-stained kidney sections as the sum of the areas of individual cysts >50µm in diameter (i.e. area ~2000 µm^2) compared to total kidney area in 4 different sections per kidney.

Thick cryo-sections (150µm) or whole permeabilized and cleared kidneys (Focus Clear; Cedarlane Laboratories) were utilized for 3D analysis of glomeruli, Proximal Tubules and collecting ducts. Image stacks were aligned, piled and concatenated with the ImageJ Image Layering Toolkit[20]. Reconstructed images were imported into Neurolucida (MBF Bioscience) and manually traced from the ureter, as described[21,22]. The number, shape, thickness and volume of glomeruli and Proximal Tubules were assayed with anti-podocalyxin and anti-AQP1 staining, respectively. UB branch generation was identified with anti-Krt8 and characterized by (1) stalk thickness, (2) stalk length, (3) branch angle, (4) branching nodes, (5) endpoints and finally (6) volume. Whole kidney pictures were obtained with a Zeiss MicroBeam IV Laser Capture/Histology/Fluorescence microscope.

Ferritin and transferrin were detected with anti-FTL and anti-transferrin immunoblots. Please see Key Resource Table for Catalogue numbers and RRIDs for immunofluorescence and immunoblots.

Analysis of Cilia

Techniques to analyze cilia were developed by Mahjoub and colleagues[23]. Paraffin-embedded sections were boiled in antigen-retrieval buffer (10mM Tris Base, 1mM EDTA, 0.05% Tween-20, pH 9.0; 30 min), then pre-extracted with 0.5% Triton X-100 in PBS (10 min, room temperature), blocked (3.0% BSA, 0.5% Triton X-100 in PBS; 1 hour), and incubated with primary antibodies overnight at 4°C. Antibodies included: mouse anti-γ-tubulin (clone GTU-88; 1:500; Sigma-Aldrich), mouse anti-acetylated tubulin (clone 6-11b-1; 1:5000; Sigma-Aldrich), rabbit anti-Arl13b (1:1000; Proteintech) and 4',6-diamidino-2-phenylindole (DAPI). Specimens were mounted in Mowiol containing n-propyl gallate (Sigma-Aldrich) and photographed using a Nikon Eclipse Ti-E inverted confocal microscope equipped with 10x Plan Fluor (0.30 NA), 20x Plan Apo air (0.75 NA), 60x Plan Fluor oil immersion (1.4 NA), or 100x Plan Fluor oil immersion (1.45 NA) objectives (Nikon). Digital optical sections (z-stacks) were reconstructed as three-dimensional images using Nikon Elements AR 4.20 (Nikon) and Photoshop software.

The percentage of ciliated cells per tubule or cyst were counted. Ciliary length was measured from three-dimensional reconstructions of z-stack images, using integrated image analysis software (Nikon Elements AR 4.20). The Arl13b fluorescence intensity per unit length cilium was determined by measuring the total fluorescence intensity of Arl13b in a selected region encompassing each cilium, divided by the ciliary length using acetylated-tubulin as marker. Background subtraction was performed by quantifying the fluorescence intensity of a region of equal dimensions in an adjacent area. The ratio of Aril13b/Cilium was determined using Excel software (Microsoft).

Statistical analysis

See figure legends for sample size and p or q-values. All data that compared two groups were tested by either Welch 2- tailed test (for parametric data) or by Mann Whitney T-test (for non-parametric). Comparison of multiple experimental groups with one independent variable were tested using Kruskal-Wallis analysis of variance (ANOVA) followed by Dunn's post hoc test. 2-way Anova was used for comparison of two independent variables on one outcome corrected with either Bonferroni or a two-stage false discovery rate (FDR) with Benjamini-Krieger-Yukutiel correction for multiple testing. To compare metrics of tubular growth in different models of iron deficiency, Proximal Tubule length, diameter, surface area, and tortuosity were normalized to their own control mean values identified in littermates. All measurements were considered continuous variables and compared between knockouts and wild types. Power Analysis: Assuming an independent sample of 5 knockouts and 5 controls, we have 80% power to detect at least a 2-fold difference in group means at alpha=0.05. This large effect size is expected[24]. Statistics were calculated with Graph Pad Prism 10.0.3.

Supplemental Table 1: Materials

Reagent type (species) or resource	Designation	Source or reference	Identifiers	Additional information
**Chemicals**				
Chemical	Roxadustat (2mg/kg)	Cayman	Cat#: 15294	HIF activation
Chemical compound	^{55}Fe citrate	Perkin Elmer	Cat#: NEZ43001	Iron delivery
Chemical compound	Iron Sucrose (Venofer 1mg)	Venofer	NDC #: 0517-2310-05	Iron delivery
Chemical compound	Nitrilotriacetic Acid	Sigma-Aldrich	Cat#: N877	Iron delivery
Chemical compound	EDU (5-ethynyl-2'-deoxy-uridine; 50mg/g); Click-iT™ EdU AlexaFluor™	Thermo Fischer Scientific	Cat#: C10337	Proliferation
Chemical compound	TRIzol Reagent	Ambion Life Technologies, Carlsbad, CA	Cat#: 15596026 and 15596018	RNA-seq
Commercial assay or kit	Cell Death: Terminal Deoxynucleotidyl Transferase mediated dUTP nick end labeling (TUNEL Kit)	Roche Diagnostics	Cat#: 12156792910	Cell Death
Commercial assay or kit	High-Capacity cDNA Reverse Transcription Kit	Applied Biosystems	Cat#: USA 4374966	Q-PCR
Commercial assay or kit	RNaqueous Micro kit extraction	Ambion	Cat#: AM1931	Screening MicroArray
Commercial assay or kit	Ovation™ RNA Amplification system V2.	Nugent/Tecan	Cat#: M01206v9	Screening MicroArray
Commercial assay or kit	FL-Ovation™ cDNA Biotin Module V2.	Nugent/Tecan	Cat#: 4200-12, -6-A01	Screening MicroArray
Commercial assay or kit	Mouse Genome 430 2.0 Arrays (Affymetrix)	Affymetrix	Cat#: 900497	Screening MicroArray
Commercial assay or kit	Bioanalyzer, RNA 6000 Chips.	Agilent 2100 with RNA Chip		RNA-seq
Commercial assay or kit	Illumina TruSeq RNA prep kits	Illumina, San Diego, CA		RNA-seq
**Mice**				
Strain, strain background (*M. musculus, C57BL/6*)	C57BL/6	Charles River, USA		Breeding
Genetic reagent (*M. musculus*)	TfR1-Loxp1-LNL-DTA BAC DNA	BACPAC Resources (http://bacpac.chori.org, Oakland, CA) and Generated by the authors		Used to Generate TfR1$^{f/f}$
Transfected construct (ES cells)	KV1 ES Cells	Generated by HICCC Transgenic Shared Resource at Columbia		Used to Generate TfR1$^{f/f}$
Strain, strain background (*M. musculus, C57BL/6*)	C57BL/6, TfR1$^{f/f}$	Generated by the authors- Cryopreserved at JaxMice: Sperm 404746		Used to Generate TfR1$^{f/f}$
Transfected construct (ES cells)	TfR1$^{-/-}$ ES cells	A very kind gift from NC Andrews: Levy et al., Nat. Genet. 1999; 21: 396-399.		Generate Ubiquitous GFP-TfR1$^{-/-}$ ES cells
Transfected construct (ES cells)	Rosa26-GFP-TfR1$^{-/-}$ ES cells	Generated by the authors: Li et al., Dev Cell 2009; 16:35-46 and Ned et al. Epub 2003 Jul 24. PMID: 12881306.		Generate Ubiquitous GFP-TfR1$^{-/-}$ ES cells
Strain, strain background (*M. musculus, C57BL/6*)	Lrp2 (Megalin) 3' CreERT	Generated by the authors Shen, T. H., Stauber, J., Xu, K., Jacunski, A., Paragas, N., Callahan, M., Banlengchit, R., Levitman, A., Desanti De Oliveira, B., Beenken, A., Grau, M. S., Mathieu, E., Zhang, Q., Li, Y., Gopal, T., Askanase, N., Arumugam, S., Mohan, S., Good, P., Stevens, J., Barasch, J. (2022). Snapshots of nascent RNA reveal cell- and stimulus-specific responses to acute kidney injury. *JCI Insight*, *7*(6), e146374. https://doi.org/10.1172/jci.insight.146374		Conditional deletion of TfR1 without reducing expression of megalin

Strain, strain background (*M. musculus, C57BL/6*)	Foxd1-Cre mice.	A very kind gift from Cathy Mendelsohn Levinson et al., 2005 Development 132(3):529-39.		Conditional deletion of TfR1
Strain, strain background (*M. musculus, C57BL/6*)	Six2-CreEGFP mice.	Kobayashi et al., 2008; Cell Stem Cell 3(2):169-81 https://www.jax.org/strain/009606		Conditional deletion of TfR1
Strain, strain background (*M. musculus, C57BL/6*)	Hoxb7-Cre mice.	A very kind gift from F Costantini: Srinivas et al., 1999; Dev Genet. 24(3-4):241-51.		Conditional deletion of TfR1
Strain, strain background (*M. musculus, C57BL/6*)	HIF1□$^{f/f}$ mice.	Ryan et al Cancer Res. 2000 Aug 1;60(15):4010-5. www.jax.org/strain/007561		Generate TfR1$^{f/f}$; HIF1□$^{f/f}$
**Antibodies**				
Antibody	Anti-aqp1 (rabbit polyclonal)	Affinity Biosciences	Cat# AF5231, RRID:AB_2837717	IF 1:200
Antibody	Anti-aqp2 (Rabbit polyclonal)	Sigma-Aldrich	Cat# A7310, RRID:AB_476762	IF 1:500
Antibody	Anti-Arl13b (rabbit polyclonal)	Proteintech	Cat# 17711-1-AP, RRID:AB_2060867	IF 1:1000
Antibody	Anti-Calb1 (mouse monoclonal)	Sigma-Aldrich	Cat# C9848, RRID:AB_2314067	IF 1:200
Antibody	Anti-Cytokeratin 8 (rat monoclonal)	Millipore	Cat# MABT329, RRID:AB_2891089	IF 1:50
Antibody	Anti-E-Cadherin (rat monoclonal)	Thermo-Fisher Scientific	Cat# 13-1900, RRID:AB_2533005	IF 1:200
Antibody	Anti-FTH1 (rabbit polyclonal)	Sigma-Aldrich	Cat# SAB2100860, RRID:AB_10604609	IF 1:200, Western Blot 1:1000
Antibody	Anti-FTL (goat polyclonal)	Sigma-Aldrich	Cat# SAB2500431, RRID:AB_10603855	IF 1:200
Antibody	Anti-Fpn1 (rabbit polyclonal)	Alpha Diagnostic International	Cat# MTP11-A, RRID:AB_1619475	IF 1:200
Antibody	Anti-Krt8 (rat monoclonal)	DSHB	Cat# TROMA-I, RRID:AB_531826	IF 1:200
Antibody	Anti-Megalin (goat polyclonal)	Santa Cruz Biotechnology	Cat# sc-16478, RRID:AB_2234897	IF 1:100
Antibody	Anti-NKCC2 (rabbit polyclonal)	Sigma-Aldrich	Cat# AV41388, RRID:AB_1854505	IF 1:200
Antibody	Anti-PAX2 (rabbit polyclonal)	Thermo Fisher Scientific	Cat# 71-6000, RRID:AB_2533990	IF 1:50
Antibody	Anti- PhosphoHistone3 (Ser10)	Cell Signaling Technologies	Cat# 9701, RRID:AB_331535	IF 1:50
Antibody	Anti-Podocalyxin (Goat polyclonal)	R and D Systems	Cat# AF1556, RRID:AB_354858	IF 1:200
Antibody	Anti-SLC12A3 (rabbit polyclonal)	Sigma- Aldrich	Cat# HPA028748, RRID:AB_10603886	IF 1:100

Antibody	Anti-SLC39A8 (rabbit polyclonal)	Proteintech	Cat# 20459-1-AP, RRID:AB_10697830	IF 1:200
Antibody	Anti-SLC39A14 (Rabbit Polyclonal)	MyBioSource	Cat#: MBS151580	IF 1:200
Antibody	Anti-Tenascin (rabbit rabbit)	Millipore	Cat# AB19013, RRID:AB_2256033	IF 1:400
Antibody	Anti-transferrin (goat polyclonal)	Bethyl	Cat# A90-129A, RRID:AB_2208812	Western Blot 1:1000
Antibody	Anti-TfR1 (rat monoclonal)	Bio-Rad	Cat# MCA2396, RRID:AB_905973	IF 1:200
Antibody	Anti-Tubulin, gamma (mouse monoclonal)	Sigma-Aldrich	Cat# T6557, RRID:AB_477584	IF 1:500
Antibody	Anti-Tubulin, acetyl-□ (mouse monoclonal)	Sigma-Aldrich	Cat# MABT868, RRID:AB_2819178	IF 1:5000
Antibody	Anti-Umod (sheep polyclonal)	Meridian Life Science	Cat# K90071C, RRID:AB 153128	IF 1:200
Antibody	Anti-WT1	Abcam	Cat# AB89901, RRID:AB_2043201	IF 1:200
**Lectin**				
Lectin	LTL	Vector Laboratories	Cat# FL-1321, RRID:AB_2336559	IF 1:50
**Primers-Genotyping**				
Sequence-based reagent	Foxd1-Cre-F	Sigma-Aldrich	Genotyping Foxd1Cre locus, forward primer	GGA CCA CAG TGG AAA AGG T C
Sequence-based reagent	Foxd1-Cre-R	Sigma-Aldrich	Genotyping Foxd1Cre locus, reverse primer	ATG TTT AGC TGG CCC AAA TG
Sequence-based reagent	Hif1-P2	Sigma-Aldrich	Genotyping Hif-1 floxed locus, forward primer	TAG TGT CTT AAC CTG CCC ATA AAT G
Sequence-based reagent	Hif1-P3	Sigma-Aldrich	Genotyping Hif-1 floxed locus, forward primer	AAA GGA AGA CAG CTC TCC TTG
Sequence-based reagent	Hif1-P1	Sigma-Aldrich	Genotyping Hif-1 floxed locus, reverse primer	GAA AAC TGT CTG TAA CTT CAT TTC C
Sequence-based reagent	Hif1-P4	Sigma-Aldrich	Genotyping Hif-1 floxed locus, reverse primer	TTA TTT AAC ATT TCC AAA GCA GAT G
Sequence-based reagent	HoxB7-Cre-F	Sigma-Aldrich	Genotyping HoxB7Cre locus, forward primer	GGT CAC GTG GTC AGA AGA GG
Sequence-based reagent	HoxB7-Cre-R	Sigma-Aldrich	Genotyping HoxB7Cre locus, reverse primer	CTC ATC ACT CGT TGC ATC GA
Sequence-based reagent	ProPax3-Cre-F	Sigma-Aldrich	Genotyping ProPax3Cre locus, forward primer	CTT TTT CGT CTC GCC TTC AC
Sequence-based reagent	ProPax3-Cre-R	Sigma-Aldrich	Genotyping ProPax3Cre locus, reverse primer	TAA GCA ATC CCC AGA AAT GC
Sequence-based reagent	Six2-CreGFP-F	Sigma-Aldrich	Genotyping Six2Cre locus, forward primer	GCG GTC TGG CAG TAA AAA CTA TC
Sequence-based reagent	Six2-CreGFP-R	Sigma-Aldrich	Genotyping Six2Cre locus, reverse primer	GTG AAA CAG CAT TGC TGT CAC TT
Sequence-based reagent	TfR1- loxp#1- F	Sigma-Aldrich	Genotyping TfR1 floxed locus, forward primer	ATG GTT CAG CGG TTA AGA GC
Sequence-based reagent	TfR1- loxp#2- F	Sigma-Aldrich	Genotyping TfR1 floxed locus, forward primer	CGT GAT ATT GCT GAA GAG CTT G
Sequence-based reagent	TfR1- loxp#1- R	Sigma-Aldrich	Genotyping TfR1 floxed locus, reverse primer	CCA TCT ACT TGC CGA GCA AG
Sequence-based reagent	TfR1- loxp#2- R	Sigma-Aldrich	Genotyping TfR1 floxed locus, reverse primer	ATC CAA GAG GTA CTG CAG TG
**PrimersRT-PCR**				

Sequence-based reagent	Adm-F	Sigma-Aldrich	qPCR	AGC ATC CAG CAG CTA CCC TA
Sequence-based reagent	Adm-R	Sigma-Aldrich	qPCR	ATG CCG TCC TTG TCT TTG TC
Sequence-based reagent	Aqp1-F	Sigma-Aldrich	qPCR	TGC TGG CGA TTG ACT ACA CTG GC
Sequence-based reagent	Aqp1-R	Sigma-Aldrich	qPCR	GGC ACC CCC AAT GAA CGG CC
Sequence-based reagent	Aqp2-F	Sigma-Aldrich	qPCR	TTG CCT CCA CTG ATG AGC GCC
Sequence-based reagent	Aqp2-R	Sigma-Aldrich	qPCR	GGG GTC CGA TCC AGA AGA CCC AGT G
Sequence-based reagent	Atp6v1b1-F	Sigma-Aldrich	qPCR	GTC TTT GCA GCC ATG GGG GTG A
Sequence-based reagent	Atp6v1b1-R	Sigma-Aldrich	qPCR	CCG TGG TCA GTG CCA GAC GT
Sequence-based reagent	BBS10-F	Sigma-Aldrich	qPCR	CAG AAA TCC TCC AGC CTC AG
Sequence-based reagent	BBS10-R	Sigma-Aldrich	qPCR	TGT TTT TGT CCT GTC CGT GA
Sequence-based reagent	BBS11-F	Sigma-Aldrich	qPCR	ATA CCA TGG ATG GCC ACT GT
Sequence-based reagent	BBS11-R	Sigma-Aldrich	qPCR	CCA CGT CAG TCA CCA CAA AC
Sequence-based reagent	BBS12-F	Sigma-Aldrich	qPCR	AAG CCG ACA TGA TGG ATT TC
Sequence-based reagent	BBS12-R	Sigma-Aldrich	qPCR	GGC TCA AGC CAA CTG CTA AC
Sequence-based reagent	BBS13-F	Sigma-Aldrich	qPCR	TCG AAG CCT TCT TTC TGC AT
Sequence-based reagent	BBS13-R	Sigma-Aldrich	qPCR	GCC AGA AGT CCA GTG AGA GG
Sequence-based reagent	BBS15-F	Sigma-Aldrich	qPCR	TTG ACA TTG GAG CTC GTG AC
Sequence-based reagent	BBS15-R	Sigma-Aldrich	qPCR	CAA TAT CAT GCG CCC TTC TT
Sequence-based reagent	BBS17-F	Sigma-Aldrich	qPCR	GGT GGA GGA GAC TTT CAC CA
Sequence-based reagent	BBS17-R	Sigma-Aldrich	qPCR	CTC GGA CTC CAC CTC ACT GT
Sequence-based reagent	BBS18-F	Sigma-Aldrich	qPCR	TGG CAG AAG TGA AGT CGA TG
Sequence-based reagent	BBS18-R	Sigma-Aldrich	qPCR	GGC AAG AGC TTG GGT TTA CA
Sequence-based reagent	BBS19-F	Sigma-Aldrich	qPCR	CAG TCC TTC ATC AGC TGC AC
Sequence-based reagent	BBS19-R	Sigma-Aldrich	qPCR	GCC AGC CAG GTC TGT CTT AG
Sequence-based reagent	BBS1-F	Sigma-Aldrich	qPCR	TTG TCT GTG CAG TCA CTC AG
Sequence-based reagent	BBS1-R	Sigma-Aldrich	qPCR	CTG CCA GGT TCT TCT TCA GG
Sequence-based reagent	BBS2-F	Sigma-Aldrich	qPCR	GGG TGG TCT AAT GGG AAG GT
Sequence-based reagent	BBS2-R	Sigma-Aldrich	qPCR	TCT ACC ACAC CAG CAA CAG C
Sequence-based reagent	BBS3-F	Sigma-Aldrich	qPCR	CCG TCG AAT TCC AAT CTT GT
Sequence-based reagent	BBS3-R	Sigma-Aldrich	qPCR	AAT ATG CCA CGG CTT GTC TT
Sequence-based reagent	BBS4-F	Sigma-Aldrich	qPCR	CTG TAC AAC CTG GGC CTT GT
Sequence-based reagent	BBS4-R	Sigma-Aldrich	qPCR	GAA GTT GAT GGC TGC ACT GA
Sequence-based reagent	BBS5-F	Sigma-Aldrich	qPCR	CTC TGA TGA CCA CAC GGA TG
Sequence-based reagent	BBS5-R	Sigma-Aldrich	qPCR	TCT CTA TTG CAA GCC CCA GT

Sequence-based reagent	BBS6-F	Sigma-Aldrich	qPCR	AGA TAC GCT GTC ACG GTG TG
Sequence-based reagent	BBS6-R	Sigma-Aldrich	qPCR	TTT GTG GTG CAA AAG GAT GA
Sequence-based reagent	BBS7-F	Sigma-Aldrich	qPCR	CAA GCC AAG CAA AGT CAA CA
Sequence-based reagent	BBS7-R	Sigma-Aldrich	qPCR	AGT CCG CAC CTC TAA GAC GA
Sequence-based reagent	BBS8-F	Sigma-Aldrich	qPCR	AGA GGC AGC TGA TGT CTG GT
Sequence-based reagent	BBS8-R	Sigma-Aldrich	qPCR	TTC AGC ATG GTG GTT GTT GT
Sequence-based reagent	BBS9-F	Sigma-Aldrich	qPCR	GAC GGG ACA TAC AAG CAG GT
Sequence-based reagent	BBS9-R	Sigma-Aldrich	qPCR	GCC TGA GAA TGA CTG CAA CA
Sequence-based reagent	Cdh16-F	Sigma-Aldrich	qPCR	AGC CTC TCA ACG ATT CCC ACG C
Sequence-based reagent	Cdh16-R	Sigma-Aldrich	qPCR	ATG CGA CCC ACC TTG CGC AT
Sequence-based reagent	Cubn-F	Sigma-Aldrich	qPCR	TGG GAT CTC CTG GAA ATG AG
Sequence-based reagent	Cubn-R	Sigma-Aldrich	qPCR	ACC GCT TGG GTA GAC ATT TG
Sequence-based reagent	Dcytb-F	Sigma-Aldrich	qPCR	TGC AGA ATG CAG AAT GGA AG
Sequence-based reagent	Dcytb-R	Sigma-Aldrich	qPCR	GCT CCC TCA CTG CTT GAC TC
Sequence-based reagent	Defb1s21-F	Sigma-Aldrich	qPCR	TTC AAG CCT CAT CTG TCA GC
Sequence-based reagent	Defb1a175-R	Sigma-Aldrich	qPCR	TGT GAG AAT GCC AAC ACC TG
Sequence-based reagent	DMT1 IRE-F	Sigma-Aldrich	qPCR	TGT TTG ATT GCA TTG GGT CTG
Sequence-based reagent	DMT1 IRE-R	Sigma-Aldrich	qPCR	CGC TCA GCA GGA CTT TCG AG
Sequence-based reagent	DMT1no1RE-F	Sigma-Aldrich	qPCR	GCT GAC TCA GTG GTG TCC AG
Sequence-based reagent	DMT1no1RE-R	Sigma-Aldrich	qPCR	GAA CAA GCT CAC CTC CGA AC
Sequence-based reagent	Edn1-F	Sigma-Aldrich	qPCR	CCA AGG AGC TCC AGA AAC AG
Sequence-based reagent	Edn1-R	Sigma-Aldrich	qPCR	GGT GAG CGC ACT GAC ATC TA
Sequence-based reagent	Eno2-F	Sigma-Aldrich	qPCR	AGC CCT CAT CAG CTC AGG
Sequence-based reagent	Eno2-R	Sigma-Aldrich	qPCR	CTT CCT TCA CCG CTC CA
Sequence-based reagent	Epo-F	Sigma-Aldrich	qPCR	AGG AGG CAG AAA ATG TCA CG
Sequence-based reagent	Epo-R	Sigma-Aldrich	qPCR	CCA CCT CCA TTC TTT TCC AA
Sequence-based reagent	Fbp1-F	Sigma-Aldrich	qPCR	TGA GGG TTA TGC CAA GGA CT
Sequence-based reagent	Fbp1-R	Sigma-Aldrich	qPCR	AGC TAT GGG GTT GCA CTC AT
Sequence-based reagent	Fth1-F	Sigma-Aldrich	qPCR	CGA GAT GAT GTG GCT CTG AA
Sequence-based reagent	Fth1-R	Sigma-Aldrich	qPCR	GTG CAC ACT CCA TTG CAT TC
Sequence-based reagent	Hamp-F	Sigma-Aldrich	qPCR	CTG CCT GTC TCC TGC TTC TC
Sequence-based reagent	Hamp-R	Sigma-Aldrich	qPCR	AGA TGC AGA TGG GGA AGT TG
Sequence-based reagent	Hamp-F	Sigma-Aldrich	qPCR	CAA CTT CCC CAT CTG CAT CT
Sequence-based reagent	Hamp-R	Sigma-Aldrich	qPCR	GGA TGT GGC TCT AGG CTA TG

Sequence-based reagent	Hba-F	Sigma-Aldrich	qPCR	ATG CGG TTA AGA GCA TCG AC
Sequence-based reagent	Hba-R	Sigma-Aldrich	qPCR	ATG AAC TTG TCC CAG GCT TC
Sequence-based reagent	Hbb-F	Sigma-Aldrich	qPCR	ATG GCA AGA AGG TGC TGA CT
Sequence-based reagent	Hbb-R	Sigma-Aldrich	qPCR	CAC CAG CAC ATT ACC CAA GA
Sequence-based reagent	Hfe-F	Sigma-Aldrich	qPCR	GAA TGG GAC GAG CAC AAG AT
Sequence-based reagent	Hfe-R	Sigma-Aldrich	qPCR	TGA TGT TCT GGG GGA AGA AG
Sequence-based reagent	HK2-F	Sigma-Aldrich	qPCR	GAA AGA TCA TCA GCG GGA T
Sequence-based reagent	HK2-R	Sigma-Aldrich	qPCR	GCC AGT GGT AAG GAG CTC TG
Sequence-based reagent	HNF1B-F	Sigma-Aldrich	qPCR	AG CCC ACC AAC AAG ATG
Sequence-based reagent	HNF1B-R	Sigma-Aldrich	qPCR	TCC TTG CTG GGA TTC TTT TG
Sequence-based reagent	Kl-F	Sigma-Aldrich	qPCR	GCA AAG TGC TCAACT GGC TA
Sequence-based reagent	Kl-R	Sigma-Aldrich	qPCR	GCC ACA AAG GTT GAT GTC GT
Sequence-based reagent	Krt8-F	Sigma-Aldrich	qPCR	GAG AGC AGG CTG GAG TCT GGG ATG C
Sequence-based reagent	Krt8-R	Sigma-Aldrich	qPCR	GTG TTG GAT CCC CCG GCA GAA C
Sequence-based reagent	Lhx1-F	Sigma-Aldrich	qPCR	AGC TCT CCA CCG GCG AGG AG
Sequence-based reagent	Lhx1-R	Sigma-Aldrich	qPCR	TCA CTG CCT GTG GTG GCC GA
Sequence-based reagent	Megalin-F	Sigma-Aldrich	qPCR	AAA CCT AGG GGA CTC GCA TT
Sequence-based reagent	Megalin-R	Sigma-Aldrich	qPCR	CCT TCG GTT CTG TCC ATC AT
Sequence-based reagent	Mep1-F	Sigma-Aldrich	qPCR	TTC AAG GGG ACA TCC TTC TG
Sequence-based reagent	Mep1-R	Sigma-Aldrich	qPCR	TCG TAG GGC TTG AAA TCC AC
Sequence-based reagent	Ndrg1 F	Sigma-Aldrich	qPCR	CGA GAG CTA CAT GAC GTG GA
Sequence-based reagent	Ndrg1 R	Sigma-Aldrich	qPCR	AAG AGG GGG TTG TAG CAG GT
Sequence-based reagent	Notch1-F	Sigma-Aldrich	qPCR	CTG CGA ATG TCC GCA TGG GC
Sequence-based reagent	Notch1-R	Sigma-Aldrich	qPCR	CAA GGG TTG GCA CCC AGA GC AC
Sequence-based reagent	NPHP1-F	Sigma-Aldrich	qPCR	GAG GCA CAC AGT GAC AAG GA
Sequence-based reagent	NPHP1-R	Sigma-Aldrich	qPCR	TTC CTT CTC CAT CCC CTT CT
Sequence-based reagent	NPHP2-F	Sigma-Aldrich	qPCR	CAC CCA TCA GCT CAG AGA CA
Sequence-based reagent	NPHP2-R	Sigma-Aldrich	qPCR	TGA TCA GCA CAG GCA ACT TC
Sequence-based reagent	NPHP3-F	Sigma-Aldrich	qPCR	ACC CTT TGC AAA CAT CAA CC
Sequence-based reagent	NPHP3-R	Sigma-Aldrich	qPCR	ACC TGA CAG GGT GAC TCC AC
Sequence-based reagent	NPHP4-F	Sigma-Aldrich	qPCR	GAC TCC TTG CTC CTC ATT GG
Sequence-based reagent	NPHP4-R	Sigma-Aldrich	qPCR	TCG TGT GAG ACC TGA ACA GC
Sequence-based reagent	NPHP5-F	Sigma-Aldrich	qPCR	GGA GGC AGA AGG AAA GGA AC
Sequence-based reagent	NPHP5-R	Sigma-Aldrich	qPCR	CAC CAG GGT GCT TGT ATG TG

Sequence-based reagent	NPHP6-F	Sigma-Aldrich	qPCR	GCA GAA GGA ATC TTC GAT GC
Sequence-based reagent	NPHP6-R	Sigma-Aldrich	qPCR	TCC GAG ATT GCC TGA GTT CT
Sequence-based reagent	NPHP7-F	Sigma-Aldrich	qPCR	CTG TGG ATT TCC AGC CAC TT
Sequence-based reagent	NPHP7-R	Sigma-Aldrich	qPCR	GGA AGC AGG TAG GTG CAG AG
Sequence-based reagent	NPHP8-F	Sigma-Aldrich	qPCR	AGC GGT GTG GCT AAG GTA GA
Sequence-based reagent	NPHP8-R	Sigma-Aldrich	qPCR	CAA GCT GGC CCT CAG ATA AG
Sequence-based reagent	NPHP9-F	Sigma-Aldrich	qPCR	GAG TCC CAT AAC TGC CCT CA
Sequence-based reagent	NPHP9-R	Sigma-Aldrich	qPCR	GGA GAG GTG AGG ATC ATG GA
Sequence-based reagent	P21-F	Sigma-Aldrich	qPCR	CGG TGG AAC TTT GAC TTC GT
Sequence-based reagent	P21-R	Sigma-Aldrich	qPCR	CAG GGC AGA GGA AGT ACT GG
Sequence-based reagent	PKD1- F	Sigma-Aldrich	qPCR	CTT CAG GTT GGT GGG TCT GT
Sequence-based reagent	PKD1- R	Sigma-Aldrich	qPCR	TTC TCA GCC TGC ACA TTC AC
Sequence-based reagent	PKD2- F	Sigma-Aldrich	qPCR	AAG GTG GTG GCA AAC TGA AC
Sequence-based reagent	PKD2- R	Sigma-Aldrich	qPCR	ATG GCC TCA ATC TCT GCA TC
Sequence-based reagent	Pkdh1- F	Sigma-Aldrich	qPCR	CTC AGT CAG CCA ACT GCA
Sequence-based reagent	Pkdh1- R	Sigma-Aldrich	qPCR	AAA CAG GCC ACA GAT AC
Sequence-based reagent	Pou3f3-F	Sigma-Aldrich	qPCR	CTAACC CCT ACCTGC CGG GG A
Sequence-based reagent	Pou3f3-R	Sigma-Aldrich	qPCR	ACC ATC TTG ACC GAG GAC GGG TCC
Sequence-based reagent	Ret-F	Sigma-Aldrich	qPCR	CCC CGA CGG CCA CTG TGA TG
Sequence-based reagent	Ret-R	Sigma-Aldrich	qPCR	AGT GGG CCC TGG CTG TCC TC
Sequence-based reagent	Slc16a3-F	Sigma-Aldrich	qPCR	ACG GCT GGT TTC ATA ACA GG
Sequence-based reagent	Slc16a3-F	Sigma-Aldrich	qPCR	CCA ATG GCA CTG GAG AAC TT
Sequence-based reagent	Slc2a1-F	Sigma-Aldrich	qPCR	GCT GTG CTT ATG GGC TTC TC
Sequence-based reagent	Slc2a1-R	Sigma-Aldrich	qPCR	CAC ATA CAT GGG CAC AAA GC
Sequence-based reagent	Slc2a3-F	Sigma-Aldrich	qPCR	GAA CCG ATC TAT GCC ACG AT
Sequence-based reagent	Slc2a3-R	Sigma-Aldrich	qPCR	GCC AGG TCC AAT CTC AAA GA
Sequence-based reagent	Slc34a1-F	Sigma-Aldrich	qPCR	CAA TGC CAT CCT ATC CAA CC
Sequence-based reagent	Slc34a1-R	Sigma-Aldrich	qPCR	GAC CAT GCT GAC AAT GAT GG
Sequence-based reagent	Slc40a1-F	Sigma-Aldrich	qPCR	T AA TGG GAA CTG TGG CCT TC
Sequence-based reagent	Slc40a1-R	Sigma-Aldrich	qPCR	GGC TCC ACA TTC ACA AAC CT
Sequence-based reagent	Spp1-F	Sigma-Aldrich	qPCR	CCC GGT GAA AGT GAC TGA TT
Sequence-based reagent	Spp1-R	Sigma-Aldrich	qPCR	CCA TCG TCA TCA TCA TCG TC
Sequence-based reagent	Tf-F	Sigma-Aldrich	qPCR	TAG GCG CAT TCA AGT GTC TG
Sequence-based reagent	Tf-R	Sigma-Aldrich	qPCR	GAG CCA CAA CAG CAT GAG AA

Sequence-based reagent	TfR1-F	Sigma-Aldrich	qPCR	GCT GCA TAT CCT TTC CTT GC
Sequence-based reagent	TfR1-R	Sigma-Aldrich	qPCR	ACC ATT TGG TTG AGC TGA GG
Sequence-based reagent	TfR2-F	Sigma-Aldrich	qPCR	CCA TCA GTG CTG ACA TTG CT
Sequence-based reagent	TfR2-R	Sigma-Aldrich	qPCR	TGT TGA CCA CAA GGC GTA AG
Sequence-based reagent	TSC1-F	Sigma-Aldrich	qPCR	CCT GAC ACC ACC AAG GAA GT
Sequence-based reagent	TSC1-R	Sigma-Aldrich	qPCR	TGG AGA AGG TGG CTT CTG TT
Sequence-based reagent	TSC2-F	Sigma-Aldrich	qPCR	TGC TGA TCA ACA GGC AGT TC
Sequence-based reagent	TSC2-R	Sigma-Aldrich	qPCR	AGC TGA GTA GCC AGC TTT CG
Sequence-based reagent	Umod-F	Sigma-Aldrich	qPCR	CCG GTT TGC AGG AAA CTA CGA CC
Sequence-based reagent	Umod-R	Sigma-Aldrich	qPCR	AGG CCT GGA CAC CTT GTC GTG
Sequence-based reagent	VHL-F	Sigma-Aldrich	qPCR	CAG GAG ACT GGA CAT CGT CA

Supplemental References

[1] Hubbard AC, Bandyopadhyay S, Wojczyk BS, Spitalnik SL, Hod EA, Prestia KA. Effect of dietary iron on fetal growth in pregnant mice. *Comp Med*. 2013;63(2):127-135.
[2] Gambling L, McArdle HJ. Iron, copper and fetal development. *Proc Nutr Soc*. 2004;63(4):553-562. doi:10.1079/pns2004385
[3] Bothwell TH. Iron requirements in pregnancy and strategies to meet them. *Am J Clin Nutr*. 2000;72(1 Suppl):257S-264S. doi:10.1093/ajcn/72.1.257S
[4] Bourque SL, Iqbal U, Reynolds JN, Adams MA, Nakatsu K. Perinatal iron deficiency affects locomotor behavior and water maze performance in adult male and female rats. *J Nutr*. 2008;138(5):931-937. doi:10.1093/jn/138.5.931
[5] Levinson RS, Batourina E, Choi C, Vorontchikhina M, Kitajewski J, Mendelsohn CL. Foxd1-dependent signals control cellularity in the renal capsule, a structure required for normal renal development. *Development*. 2005;132(3):529-539. doi:10.1242/dev.01604
[6] Kobayashi A, Valerius MT, Mugford JW, et al. Six2 defines and regulates a multipotent self-renewing nephron progenitor population throughout mammalian kidney development. *Cell Stem Cell*. 2008;3(2):169-181. doi:10.1016/j.stem.2008.05.020
[7] Srinivas S, Goldberg MR, Watanabe T, D'Agati V, al-Awqati Q, Costantini F. Expression of green fluorescent protein in the ureteric bud of transgenic mice: a new tool for the analysis of ureteric bud morphogenesis. *Dev Genet*. 1999;24(3-4):241-251. doi:10.1002/(SICI)1520-6408(1999)24:3/4<241::AID-DVG7>3.0.CO;2-R
[8] Shen TH, Stauber J, Xu K, et al. Snapshots of nascent RNA reveal cell- and stimulus-specific responses to acute kidney injury. *JCI Insight*. 2022;7(6):e146374. Published 2022 Mar 22. doi:10.1172/jci.insight.146374
[9] Li JY, Paragas N, Ned RM, et al. Scara5 is a ferritin receptor mediating non-transferrin iron delivery. *Dev Cell*. 2009;16(1):35-46. doi:10.1016/j.devcel.2008.12.002
[10] Zhang D, Okada S, Kawabata T, Yasuda T. An improved simple colorimetric method for quantitation of non-transferrin-bound iron in serum. *Biochem Mol Biol Int*. 1995;35(3):635-641.
[11] Gosriwatana I, Loreal O, Lu S, Brissot P, Porter J, Hider RC. Quantification of non-transferrin-bound iron in the presence of unsaturated transferrin. *Anal Biochem*. 1999;273(2):212-220. doi:10.1006/abio.1999.4216
[12] Hod EA, Zhang N, Sokol SA, et al. Transfusion of red blood cells after prolonged storage produces harmful effects that are mediated by iron and inflammation. *Blood*. 2010;115(21):4284-4292. doi:10.1182/blood-2009-10-245001
[13] Bolger AM, Lohse M, Usadel B. Trimmomatic: a flexible trimmer for Illumina sequence data. *Bioinformatics*. 2014;30(15):2114-2120. doi:10.1093/bioinformatics/btu170
[14] Kim D, Pertea G, Trapnell C, Pimentel H, Kelley R, Salzberg SL. TopHat2: accurate alignment of transcriptomes in the presence of insertions, deletions and gene fusions. *Genome Biol*. 2013;14(4):R36. Published 2013 Apr 25. doi:10.1186/gb-2013-14-4-r36
[15] Anders S, Pyl PT, Huber W. HTSeq--a Python framework to work with high-throughput sequencing data. *Bioinformatics*. 2015;31(2):166-169. doi:10.1093/bioinformatics/btu638
[16] Love MI, Huber W, Anders S. Moderated estimation of fold change and dispersion for RNA-seq data with DESeq2. *Genome Biol*. 2014;15(12):550. doi:10.1186/s13059-014-0550-8
[17] Kamburov A, Pentchev K, Galicka H, Wierling C, Lehrach H, Herwig R. ConsensusPathDB: toward a more complete picture of cell biology. *Nucleic Acids Res*. 2011;39(Database issue):D712-D717. doi:10.1093/nar/gkq1156
[18] Vainio S, Lin Y. Coordinating early kidney development: lessons from gene targeting. *Nat Rev Genet*. 2002;3(7):533-543. doi:10.1038/nrg842
[19] Fähling M, Mathia S, Scheidl J, et al. Cyclosporin a induces renal episodic hypoxia. *Acta Physiol (Oxf)*. 2017;219(3):625-639. doi:10.1111/apha.12811
[20] Schneider CA, Rasband WS, Eliceiri KW. NIH Image to ImageJ: 25 years of image analysis. *Nat Methods*. 2012;9(7):671-675. doi:10.1038/nmeth.2089

[21] Sampogna RV, Schneider L, Al-Awqati Q. Developmental Programming of Branching Morphogenesis in the Kidney. *J Am Soc Nephrol*. 2015;26(10):2414-2422. doi:10.1681/ASN.2014090886
[22] Dan H, Ruan T, Sampogna RV. Circadian Clock Regulation of Developmental Time in the Kidney. *Cell Rep*. 2020;31(7):107661. doi:10.1016/j.celrep.2020.107661
[23] Hoshi M, Wang J, Jain S, Mahjoub MR. Imaging centrosomes and cilia in the mouse kidney. *Methods Cell Biol*. 2015;127:1-17. doi:10.1016/bs.mcb.2014.12.008
[24] Werth M, Schmidt-Ott KM, Leete T, et al. Transcription factor TFCP2L1 patterns cells in the mouse kidney collecting ducts. *Elife*. 2017;6:e24265. Published 2017 Jun 3. doi:10.7554/eLife.24265
